# Oxidative stress in cancer: from tumor and microenvironment remodeling to therapeutic frontiers

**DOI:** 10.1186/s12943-025-02375-x

**Published:** 2025-08-22

**Authors:** Xisong Liang, Jiadi Weng, Zhongyi You, Yang Wang, Jie Wen, Zhiwei Xia, Shaorong Huang, Peng Luo, Quan Cheng

**Affiliations:** 1 Department of Neurosurgery, Xiangya Hospital, Central South University, Changsha, 410008 P. R. China; 2 Hypothalamic Pituitary Research Centre, Xiangya Hospital, Central South University, Changsha, Hunan 410008 P. R. China; 3 National Clinical Research Center for Geriatric Disorders, Changsha, 410008 P. R. China; 4 Xiangya School of Medicine, Central South University, Changsha, 410013 P. R. China; 5 Department of Neurology, Hunan Aerospace Hospital, Hunan Normal University, Changsha, Hunan 410205 P. R. China; 6 Institute of Geriatrics, Jiangxi Provincial People’s Hospital, The First Affiliated Hospital of Nanchang Medical College, Nanchang, 330006 P. R. China; 7 Department of Oncology, Zhujiang Hospital, Southern Medical University, Guangzhou, 510280 P. R. China

**Keywords:** ROS, Antioxidants, Immunocytes, Immunotherapy, Nanomedicine

## Abstract

Oxidative stress is a pathological condition of redox signaling dysregulation and macromolecular oxidative damage arising from elevated ROS levels. Oxidative stress interacts with tumor cell growth regulation and tumor microenvironment remodeling, and has been a critical hallmark of cancer. Targeting oxidative stress has garnered great attention in cancer therapy development. However, it is still challenging due to the complexity and heterogeneity of oxidative stress regulation across different cancers, and this encourages a comprehensive understanding of the oxidative stress network in cancers to overcome this obstacle. Therefore, we introduced the oxidative stress generation and regulatory network within tumor cells and discussed their roles in both tumor cells and the tumor microenvironment. Subsequently, we summarized the current therapeutic strategies and highlighted emerging clinical applications, providing an up-to-date overview of oxidative stress-based approaches. Particularly, their cross-application with immunotherapy and nanomedicine has provided an excellent opportunity to integrate multiple effects, exhibiting surpassing advantages. This review elaborates on oxidative stress in cancer biology and its therapeutic implications. By integrating current knowledge and the emerging coordination with immunotherapy and nanomedicine, we underscore the potential of oxidative stress-targeting approaches. Future research on overcoming therapeutic resistance and developing compatible platforms to combine multiple approaches will pave the way to cancer elimination.

## Introduction

Oxidative stress is a pathological imbalance of redox signaling, accompanied by cellular macromolecular oxidative damage arising from excessive ROS (reactive oxygen species), which will lead to dysregulation of cellular signaling pathways and cell death. Elevated oxygen metabolism and mutations in related proto-oncogenes cause ROS accumulation during the early stages of tumor development, resulting in excessive ROS production in tumor cells. This cytotoxic process can lead to a series of dysregulated redox signaling pathways and molecular damage and participate in various pathological processes, including the occurrence of tumors [1], thereby forming a vicious cycle. Tumor metabolic reprogramming, along with the increased metabolic flux required for rapid cell division, is accompanied by excess ROS production. The corresponding antioxidative response is activated to protect cells from oxidative damage and promote their survival [2]. This cell-initiated series of protective antioxidation reactions that reduce ROS damage is called the adaptive responses to oxidative stress [3]. ROS also exerts bidirectional effects on cell growth, and the dynamic and complex regulation of the oxidative stress system contributes significantly to cancer progression. The O_2_•^−^ (superoxide anion) is an important source of ROS, primarily derived from the respiratory chain and some oxidases in cells that can damage macromolecules and contribute to disease onset [4, 5]. Various antioxidant enzymes can convert O_2_•^−^ into H_2_O_2_ (hydrogen peroxide) with a concentration-dependent duality. The H_2_O_2_ modulates the cell signals at physiological levels while triggering cell damage under pathological accumulation [6]. The complex crosstalk between ROS generation and scavenging system significantly impacts tumor development. Targeting the key nodes that regulate oxidative stress represents a promising therapeutic strategy.

The TME (tumor microenvironment) is a specialized niche comprising abundant non-tumor cells, extracellular matrix, and aberrant vasculature, which are embedded within various biophysical gradients. Non-tumor cells in the TME mainly include fibroblasts, lymphocytes, and macrophages [7]. Tumor cells exert complex interactions with non-tumor cells to promote the stromal barrier remodeling, aberrant angiogenesis, and immunosuppressive cytokines release to survive and invade [8, 9]. Therefore, the TME cells play a pivotal role in tumor development [10]. CAFs (cancer-associated fibroblasts) are the key stromal cells in the TME and can secrete extracellular matrix components to form the physical structural scaffold and perform many other biological functions, such as promoting tumor cell proliferation and supporting angiogenesis [11]. It has been proven that CAFs located in both the tumor infiltration area and normal tissue, and fibroblasts outside the tumor margin, can promote tumor proliferation and angiogenesis [12, 13]. Macrophages represent a predominant immunocyte population within the TME, constituting a significant proportion of the immunocyte compartment in some solid tumors [14, 15]. TAMs (tumor-associated macrophages) exhibit high heterogeneity across different tumors and populations, reflecting their strong adaptability to environmental changes [16]. They play a bidirectional role in tumor development, which stems from their adaptability to signals within the TME [17]. Undifferentiated macrophages can polarize into pro-inflammatory and anti-tumor M1 macrophages upon stimulation by factors such as IFN (interferon)-γ and lipopolysaccharide [18]. Conversely, IL-4 and IL-13 drive macrophage M2 polarization with anti-inflammatory and pro-tumor roles. In addition, various tumor-derived factors regulate TAM M1 and M2 polarization, affecting TME intercellular crosstalk and anti-tumor immunity [18]. Other cells, including MDSCs (myeloid-derived suppressor cells) and endothelial cells, are recruited by tumor cells to participate in immunosuppression and angiogenesis, thereby promoting tumor progression [19, 20]. Therefore, therapies targeting the high plasticity of microenvironmental cells can block their pro-tumor functions and reverse the local immunosuppressive status, thereby controlling tumor progression. ROS also plays an important role in the TME. ROS serves as a signaling messenger and is required for functional maintenance in anti-tumor immunocytes. For instance, T-cell activation relies on the transient generation of a physiological level of ROS and the ROS-dependent NF-κB- and AP-1-related pathways [21]. CD8 + T-cells’ 3D motility and infiltration into solid cancers require a sustained mitochondrial ROS level [22]. However, abnormal ROS elevation in TME impeded T-cell response [23] and remodels the TME through mechanisms such as TAM polarization and Treg recruitment. ROS can drive the polarization of TAMs toward the M2 phenotype, enhancing immunosuppression and tumor progression [24–26]. Additionally, TME ROS recruits and activates Tregs, further suppressing anti-tumor immunity and facilitating tumor growth [27].

Given the pivotal role of ROS in tumor progression, oxidative stress targeting has been developed as an effective therapeutic strategy to remodel the TME and inhibit tumor cells, thereby enhancing the efficacy of conventional treatments and immunotherapy in controlling tumors [28]. In this review, we summarized the mechanisms of ROS generation and regulation and their effects on tumor cells and the TME. We focused on presenting a series of therapeutic strategies and clinical trials and developing emerging clinical application fields based on ROS and tumor adaptive responses to oxidative stress.

## The mechanism of endogenous ROS generation

The concept of oxidative stress was initially proposed in 1985 and has since been widely discussed. Its concept and connotation have also been updated [1] due to the complexity and the increasing understanding of oxidative stress. Some viewpoints suggest that oxidative stress could be reckoned as the sudden or prolonged increase in ROS that were originally at steady­state levels, disrupting cellular metabolism and other signaling pathways, which may eventually lead to macromolecular damage or cell death [29]. It has also been proposed that oxidative stress should include macromolecular oxidative damage with redox signaling and control disruption [30]. Meanwhile, oxidative stress should be emphasized as a pathological condition, while the physiological oxidant generation is considered as oxidative eustress [31]. Synthesizing these perspectives, oxidative stress could thus be comprehensively summarized as a pathological condition of either compromised redox signaling or macromolecular oxidative damage arising from dysregulated ROS elevation, leading to disruption of cellular signaling pathways and cell death. Mammals can generate ROS through various pathways [32]. One of the major sources of ROS is the mitochondrial respiratory chain [33]. The mitochondrial respiratory chain is primarily composed of four enzyme complexes, including ​​Complex I, II, III, and IV [34]. Mitochondrial complex I contains an FMN (flavin mononucleotide) cofactor that can bind NADH (nicotinamide adenine dinucleotide, reduced form) and accept electrons from NADH. Additionally, it incorporates iron-sulfur (Fe-S) clusters within its protein subunits to facilitate electron transfer to Q (ubiquinone) by interconverting ferrous (Fe^2+^) and ferric (Fe^3+^) ions, thereby generating QH2 (ubiquinol). Consequently, the mechanism underlying O_2_•^−^ generation through complex I mainly involves two processes, with FMN serving as the mediator of electron transfer [35]. The first process generates O_2_•^−^ by electrons from NADH oxidation at the FMN site. In contrast, the second O_2_•^−^-generating pathway involves RET (reverse electron transfer), which requires two critical conditions to drive electron flow in the reverse direction: an elevated proton motive force (Δp, composed of both proton concentration gradient and membrane potential) and a highly reduced ubiquinone pool (QH2/Q ratio) [36–38]. In mitochondrial complex II, the electrons from succinate oxidation are accepted by FAD (flavin adenine dinucleotide) and subsequently transferred to Q via the Fe-S cluster [39]. The Q is then reduced to QH_2_ once it receives electrons and protons. After the electrons flow from mitochondrial complex I- and II-derived QH_2_ to cytochrome C in mitochondrial complex III, an unstable Q•^−^ species is generated and reacts with oxygen to produce O_2_•^−^ [39]. The produced O_2_•^−^ from mitochondrial complexes I and III is generated in the mitochondrial matrix and intermembrane space, respectively [40].

**Fig. 1:**
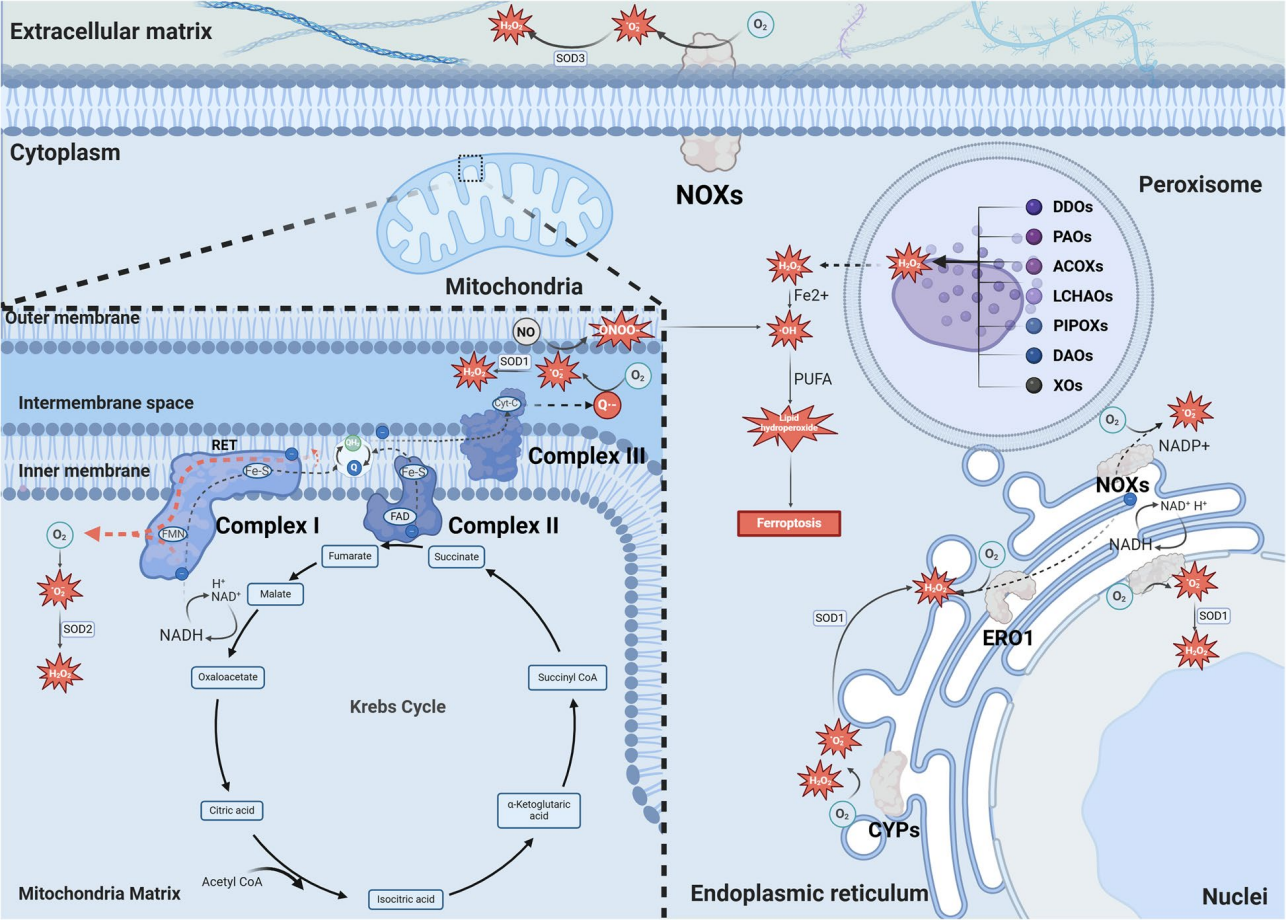
Main processes of endogenous ROS production in eukaryotes. The diagrammatic sketch exhibits ROS production via the mitochondrial electron transport chain, the ER, the peroxisome, the nucleus, and the cytoplasm. The arrows and dashed arrows represent the activating effects and particle flow, respectively. The red dashed arrow refers to RET. ROS, reactive oxygen species. ER, endoplasmic reticulum. RET, reverse electron transfer. The figure was created with BioRender.com

In addition to the mitochondrial electron transport chain, the NOXs (nicotinamide adenine dinucleotide phosphate oxidases) are also critical in ROS generation [41]. The transmembrane NOXs are located in the nucleus, cell membrane, and ER (endoplasmic reticulum), generating O_2_•^−^ directly in the nucleus compartment, mitochondrial matrix, and extracellular space [41]. The O_2_•^−^ is less diffusible and has difficulty crossing the lipid cell membrane. Instead, they are transported via anion channels [42, 43]. Compared to O_2_•^−^, NO (nitric oxide) is more stable and highly diffusible [44]. NO could react with O_2_•^−^ close to the microdomains where the O_2_•^−^ is generated. The reaction between these two species approaches the diffusion-controlled limit, rapidly producing ONOO^−^ (peroxynitrite) [45]. Thus, ONOO^−^ may directly damage macromolecules [10]. To neutralize cytotoxic ROS, the SOD (superoxide dismutase) isoforms are utilized for O_2_•^−^ elimination that catalyze its dismutation into H_2_O_2_. The SOD family exhibits distinct subcellular location: SOD1 widely localizes to the mitochondrial intermembrane space, cytosol, and nucleus compartment; SOD2 predominantly resides in the mitochondrial matrix; while SOD3 functions as a secretory isoform that is transported to extracellular space [41, 46]. In addition, H_2_O_2_ could also be generated in the ER and peroxisomes [33, 34]. Unlike other NOXs, the NOX4 in the ER can directly sense pO_2_ and may generate H_2_O_2_ independent of internal O_2_•^−^ dismutation [47]. Beyond the well-characterized NOX family, the ER harbors additional redox-active enzymatic systems contributing to ROS generation, including CYP (cytochrome P450) and ERO1 (endoplasmic reticulum oxidoreductin 1). CYP belongs to a family of heme monooxygenases capable of self-oxidation and is mostly present in the ER [48]. During the CYP reaction cycle, the uncoupling occurs with the incomplete substrate oxidation, which could generate O_2_•^−^ and H_2_O_2_ in the ER [49, 50]. ERO1 can transfer electrons from reduced protein disulfide isomerase to oxygen, thereby generating H_2_O_2_ [51]. Peroxisome is involved in distinct metabolic pathways, and the metabolic enzymes, including acyl-CoA oxidases, D-aspartate oxidase, D-amino acid oxidase, polyamine oxidase, xanthine oxidase, L-α-hydroxy acid oxidase, L-pipecolic oxidase, and sarcosine oxidase (Fig. 1), are closely intertwined with ROS generation [52]. Specifically, acyl-CoA oxidases are responsible for initiating the β-oxidation of fatty acids. In contrast, D-amino acid oxidase and D-aspartate oxidase facilitate the catabolism of non-proteinogenic amino acids. Polyamine oxidase is involved in the metabolic processing of polyamines, whereas xanthine oxidase catalyzes the conversion of hypoxanthine to xanthine and subsequently to uric acid. These enzymatic reactions generate ROS as a byproduct [41, 53]. In contrast to NOXs, which require assembly with regulatory subunits such as p22phox and Rac GTPases for activation, peroxisomal oxidases are mainly flavoproteins [52] with FAD or FMN cofactors intrinsically incorporated to enable direct reduction of molecular oxygen to generate H_2_O_2_.

H_2_O_2_ can traverse cell membranes via simple diffusion and peroxiporin channel transportation. A notable example of its simple diffusion is that H_2_O_2_ penetrates human red blood cell membranes primarily through the lipid fraction independent of peroxiporins [54, 55]. However, peroxiporin-facilitated H_2_O_2_ transmembrane diffusion has been recognized as its primary transmembrane pathway [56]. The peroxiporins are a subgroup of AQPs (aquaporins) and contain AQP0, 1, 3, 5, 6, 7, 8, 9, and 11 [57]. They facilitate the transport of H_2_O_2_ across membranes in addition to H_2_O or glycerol transport. These channels participate in redox signaling [58] and demonstrate elevated expression in human malignancies, promoting tumor cell proliferation and metastasis [59–61]. Notably, lower concentrations of H_2_O_2_ can selectively impact protein oxidation, thereby playing a pivotal role in signal transduction. Conversely, elevated H_2_O_2_ concentrations can cause oxidative stress-induced damage [62]. H_2_O_2_ can be converted to •OH (hydroxyl radicals). •OH is produced via the Fenton reaction of H_2_O_2_ with Fe^2+^ and the decomposition of ONOO^−^ [63]. In the presence of PUFAs (polyunsaturated fatty acids), •OH can react with them to form distinct lipid peroxides, which induce various cell processes in a concentration- and composition-dependent manner, including ferroptosis, apoptosis, inflammation, and autophagy [64, 65]. These different forms of ROS may exhibit different functions and targets that contribute to cell fate control.

## The network of intracellular ROS regulation

Excessive levels of O_2_•^−^, ONOO^−^, H_2_O_2_, •OH, and other ROS can damage cellular macromolecules, such as DNA, proteins, and lipids. However, tumor cells activate adaptive antioxidant pathways to maintain redox homeostasis under oxidative stress conditions [66].

### The ROS-responsive transcription factors trigger antioxidant enzyme gene expression

The initiation of antioxidant defense ​​primarily relies on​​ transcriptional activation of antioxidant enzyme genes. A critical pathway for cells to erase ROS accumulation is the NRF2-ARE (antioxidant response elements) -antioxidant axis, which is activated in response to low and moderate levels of ROS. NRF2 is a well-known transcription factor for antioxidant reaction [67]. Under physiological conditions, the Neh2 domain of the NRF2 binds to the Kelch domain of the KEAP1 (Kelch-like ECH-associated protein 1) in the cytoplasm. KEAP1 is the substrate adaptor of Cullin3-based E3 ubiquitin ligase complex, and KEAP1’s interaction with NRF2 to facilitates its ubiquitination and proteasomal degradation, thereby limiting its nuclear translocation and transcriptional activity through enhancer binding [68]. NRF2 activation upregulates various antioxidant enzymes, including HO-1 (heme oxygenase 1). The oxidative stress-controlled HO-1 could promote the displacement of BACH1 (BTB domain and CNC homolog 1) from the enhancer [69]. BACH1 is a competitor of NRF2, and they share small Maf proteins to form the heterodimer for ARE binding, thus repressing transcriptional activation of NRF2-mediated antioxidant gene expression [69]. BACH1 remains stable and blocks the binding between NRF2 and AREs under physical conditions, whereas, under oxidative stress, it undergoes E3 ligase HOIL-1-mediated degradation mediated by free heme derived from oxidized heme-containing proteins [70]. Also, heme decreases the DNA binding activity and promotes the nuclear export of BACH1, further suppressing its inhibitory effects on NRF2 [70]. Whereas, increased hemin rapidly induces the HO-1 expression, which degrades heme and releases iron to promote ROS accumulation. ROS could oxidize the reactive cysteine residues in KEAP1 [71], inducing its conformational changes and inactivation, preventing NRF2 ubiquitination and degradation. Newly synthesized NRF2 then translocates to the nucleus and accumulates to activate the transcription of the target genes [72]. Therefore, these complex networks maintain a dynamic regulation of antioxidant response under oxidative stress. In addition to the KEAP1-dependent regulation, the stability of NRF2 is governed by GSK-3β (glycogen synthase kinase-3 beta). The kinase GSK-3β phosphorylates the serine residues of the DSGIS motif located in the Neh6 domain of NRF2. This post-translational of NRF2 enables subsequent recognition by the β-TrCP-CUL1-based E3 ubiquitin ligase complex, targeting NRF2 for proteasomal degradation [73]. Meanwhile, since Akt phosphorylates and inhibits GSK-3β [74], activation of the PI3K/AKT pathway suppresses GSK-3β-mediated NRF2 phosphorylation, whereas PTEN-dependent AKT inhibition enhances GSK-3β activity and subsequent NRF2 modification [73]. For instance, NRF2 inhibitors such as brucein D could block NRF2 activities by activating the PI3K/AKT pathway [75]. When NRF2 translocates into the cell nucleus and heterodimerizes with small Maf proteins, they bind to AREs to initiate the transcription of a series of antioxidant enzyme genes, including *GCLC, GCLM, GSTM1, GPX4, GSR, TXN1, PRDX1, SRXN1,* and *NQO1* [76–80], etc. Therefore, NRF2-mediated antioxidant transcriptional regulation is critical for tumor cells to counteract oxidative stress. Notably, NRF2 not only regulates the redox homeostasis but also directly affects the survival and proliferation of tumor cells [81].

In addition to the NRF2-ARE axis described above, several other antioxidant transcription regulations contribute to redox homeostasis. When oxidative stress surpasses the antioxidant capacity of the NRF2-mediated system, excessive ROS hierarchically activate other antioxidant transcription factors through distinct redox-sensing thresholds [82]. Elevated H_2_O_2_ induced AP-1 activation and it triggers the transcription of SODs. Besides, the antioxidant response can also be initiated through PPARγ (peroxisome proliferator-activated receptor gamma), and NF-κB (nuclear factor-kappa B), activating the transcription of genes such as *GSTs* and *SODs* [1, 20, 83, 84]. The ROS accumulation can also activate the AMPK (AMP-activated protein kinase) pathway to inhibit mTOR (mammalian target of rapamycin) and activate FOXO (forkhead box O) in tumor cells, promoting metabolic reprogramming to alleviate ROS accumulation [85]. Both AMPK and NRF2 pathways can respond to oxidative stress, and their complex interaction regulates ROS levels in tumor cells [86]. Meanwhile, the activated FOXO by upstream signals is also a critical redox transcript factor as already been recently summarized [87, 88]. Once activated and translocated into the nucleus, FOXOs bind to transcription regulators such as acetyl transferases to drive the acetylation of histones and FOXOs themselves, enhancing chromatin remodelling and DNA binding to trigger the transcription of their target genes, including *SOD2*, *SOD3*, *CAT*, *PRDX3*, and *SENP.* Furthermore, ROS-induced IKK (inhibitory kappa B kinase) induces IκB (inhibitor of NF-κB) phosphorylation, enabling NF-κB nuclear translocation via the canonical pathway. This activated NF-κB pathway subsequently increases antioxidant gene expression, and thus alleviates oxidative damage [84]. The well-known tumor-suppressing transcription factor p53 exhibits bidirectional roles in redox homeostasis, demonstrating both antioxidant and pro-oxidant activities. P53 was found to upregulate the *GLS2* expression under oxidative stress or non-stress conditions, and the elevated GLS2 (glutaminase 2) increases reduced GSH levels to enhance cellular antioxidant defence [89]. Meanwhile, p53 exerts a pro-oxidant effect by upregulating PIGs (TP53-induced genes) such as *PIG1–13* under severe oxidative stress conditions. These PIGs facilitate ROS amplification through redox-cycling quinones and p67phox-mediated activation of the NOX2 complex [90–93]. PIGs and antioxidant genes induced by p53 have opposite roles, with the former promoting ROS accumulation and apoptosis under severe stress, while the latter reduce ROS and protect cells under mild stress [90, 91].

### The antioxidant enzymes compose distinct redox systems against oxidative stress

**Fig. 2:**
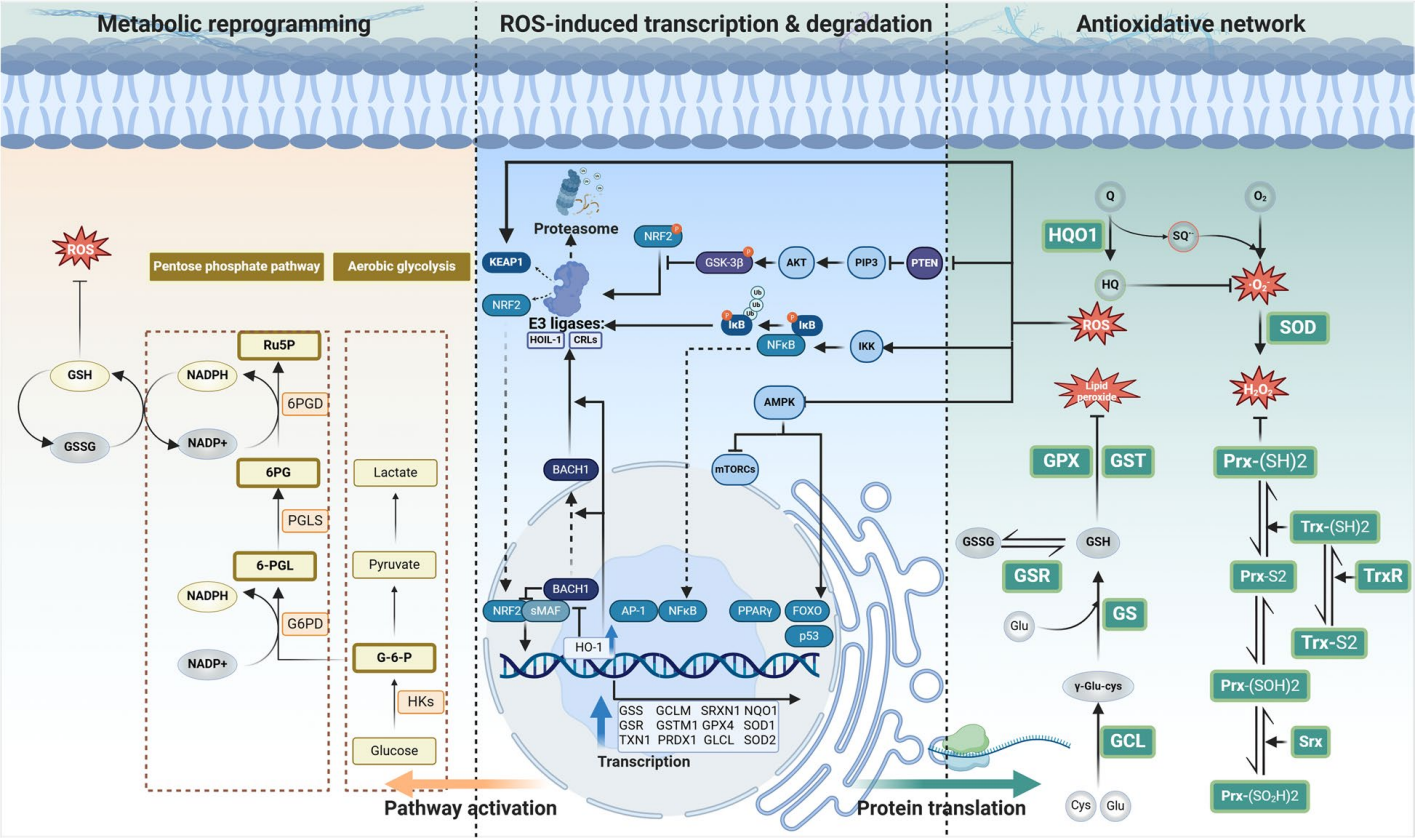
Intercellular antioxidative network for ROS regulation. High ROS pressure triggers the translocation of transcription factors to initiate the expression of various antioxidant enzymes, which scavenge ROS when supplemented with antioxidants. Metabolic reprogramming is triggered to produce antioxidative substrates for ROS resistance. Arrows, inhibitory arrows, and dashed arrows represent activating or transforming, inhibitory effects, and translocation, respectively. The spherical symbols labeled ‘P’ and ‘Ub’ indicate phosphorylation and ubiquitination, respectively. The dual arrows indicate mutual transformations. ROS, reactive oxygen species. The figure was created using BioRender.com

The transcribed mRNA of these genes encodes the corresponding enzyme proteins that participate in multiple antioxidative reactions (Fig. 2). The GSH-associated system is critical in antioxidative defense and contains various redox enzymes. *GCLC* and *GCLM* encode the catalytic and regulatory subunits of GCL (glutamate-cysteine ligase), which synthesizes γ-Glu-Cys from glutamic acid and cysteine. γ-Glu-Cys is converted into GSH (glutathione) by GS (glutathione synthetase), encoded by the *GSS* gene [94]. The generated GSH can directly scavenge ROS through the reductive activity of its thiol group [95] or function as a cofactor of antioxidant enzymes to reduce peroxides. GSH primarily reduces H_2_O_2_ to H_2_O through the enzymatic actions of GPXs (glutathione peroxidases) or PRDX6 (peroxiredoxin 6), the latter being the peroxiredoxin family member exhibiting GPx-like activity [96, 97]. GSTs (Glutathione S-transferases) catalyze the conjugation of GSH via its thiol group to electrophilic centers on diverse substrates, thereby enhancing the compounds’ water solubility. This biochemical mechanism facilitates the elimination of toxic endogenous compounds such as lipid peroxides [98, 99]. During the GSH-dependent reduction of peroxides, GSH is oxidized to its disulfide form (GSSG) [96, 97]. The GSR (glutathione-disulfide reductase), encoded by the *GSR* gene, can then reduce oxidized glutathione (GSSG) back to its reduced form (GSH) using electrons from NADPH (nicotinamide adenine dinucleotide phosphate), replenishing the cellular GSH pool [100]. Tumor cells exhibit the Warburg effect, indicating that they could undergo glycolysis under oxygen-rich conditions [101]. The Warburg effect triggers the PPP (pentose phosphate pathway) pathway for tumor cell nucleotide synthesis, and this process generates NADPH for GSH reduction and maintains redox homeostasis [85].

The Trx-Prx-Srx system is a hierarchically cooperative antioxidant network and is critical for peroxide elimination to maintain redox homeostasis. The Trx (thioredoxin) encoded by the gene *TXN1* is a key protein in the Trx system. It functions together with TrxR (thioredoxin reductase) to inhibit protein disulfide bond formation. TrxR can use NADPH as an electron donor to reduce Trx, thus restoring its ability to reduce oxidized proteins [102, 103]. Human Prx (peroxiredoxin) is encoded by gene *PRDX1* and possesses a conserved enzymatic cysteine known as the Cp (peroxidatic cysteine) at its N-terminus [104]. The Prx protein family catalyzes the reduction of H_2_O_2_. During this catalytic cycle, the thiol group (-SH) of the Cp undergoes oxidation to form sulfenic acid (-SOH) [105]. The reduced Trx could transfer electrons to cytoplasmic Prx [106], facilitating the reduction of oxidized Prx. Srx (sulfiredoxin) is a unique enzyme for Prx reductive capability repair that reduces the sulfinic acid form of 2-Cys Prx [107]. Human Srx contains only a single cysteine residue, necessitating the involvement of external thiol (such as Trx or GSH) to regenerate its active site by reducing the thiosulfinate intermediate formed during catalysis [108]. The Srxs present functional convergence with Trx in regulating Prx redox homeostasis, for it reduces the hyperoxidized Prx, such as Prx-(SO_2_H)2, to Prx-(SOH)2, which are further reduced by Trx [109], forming the Trx/Srx/Prx system to eliminate H_2_O_2_.

NQO1 enzyme belongs to the NAD(P)H quinone oxidoreductase family and is activated by transcription factor NRF2 [80, 110]. It directly reduces quinones to hydroquinone and prevents the generation of SQ•^−^ (semiquinone radicals). SQ•^−^ can reduce oxygen to O_2_•^−^, which can be eliminated by hydroquinone [111]. Some previously proposed viewpoints have classified O_2_•^−^ as oncogenic and H_2_O_2_ as onco-suppressive ROS [112]. Varying the ratio of O_2_•^−^ to H_2_O_2_ has been suggested to impact tumor progression. Although more convincing evidence is required to elucidate their precise roles in the cancer process, this underscores the importance of regulating the O_2_•^−^ -to- H_2_O_2_ ratio, representing a pivotal upstream factor in ROS dynamics to modify tumor fate. The SOD family is a critical antioxidant enzyme family that reduces the cytotoxic O_2_•^−^ burden. SOD expression could be triggered by NF-κB [84]. This family includes three members with different subcellular locations: SOD1 and SOD2 are located in the cytoplasm and mitochondria, respectively, while SOD3 is secreted to the extracellular matrix. These SODs are efficient O_2_•^−^ scavenger due to their capability to dismuate O_2_•^−^ to H_2_O_2_, which is then further eliminated by other enzymes as discussed above (Fig. 2) [82]. All the aforementioned antioxidant enzymes and substances constitute a complex regulatory network of the antioxidant response. Tumor cells utilize the antioxidant response to eliminate cytotoxic ROS and avoid ROS-induced cell injury and death.

## The regulatory role of ROS and adaptive responses to oxidative stress in tumor cells

### ROS elevation increases the risk of tumorigenesis and tumor cell growth

While causing macromolecule damage, ROS also functions as a critical mediator for tumor initiation and progression, and elevated ROS levels in tumors have been widely proven [113]. Genomic instability is commonly regarded as the main driving force of tumorigenesis and a major contributor of tumor heterogeneity [8]. Elevated ROS can cause direct DNA damage, disrupt replication and transcription, and impair the function of DNA repair enzymes. ROS can target nucleic acids to induce oxidized bases, such as 8-hydroxy-2′-deoxyguanosine (8-OH-dG) [114]. •OH directly damages DNA by oxidizing all four DNA bases [115]. These oxidized lesions impair polymerase activity. ROS-induced polymerase impairment as well as the DNA double-strand breaks both contribute to replication fork collapse [116]. Meanwhile, ROS promotes the dissociation of PRDX2 and TIMELESS protein, which also attenuates replication fork progression [117]. Besides, H_2_O_2_ extracts hydrogen atoms from deoxyribose, forming oxidized AP (apurinic/apyrimidinic) sites and DNA strand breaks [114]. ROS’ DNA targeting can also induce DNA-protein crosslinks, potentially leading to chromosomal aberrations or breaks [118]. Current studies demonstrate that ROS can generate single- and double-strand DNA breaks in transcriptionally active regions, inducing R-loop formation and threatening genomic stability [119]. H_2_O_2_ induces significant ubiquitination of Rpb1, the largest subunit of RNA polymerase II. This process may depend on Ser-5 phosphorylation mediated by ERK1/2, consequently impairing transcriptional activity [120]. ROS also inhibits OGG1 (an 8-oxoG DNA glycosylase), blocking the initiation of 8-oxoG lesion repair [121]. These oxidized damages lead to genomic instability with increased risk of oncogenic mutations to promote cancer initiation and development [9, 122–124]. In the early stages of cancer, ROS can induce mutations in the proto-oncogene *RAS* and tumor suppressor gene *TP53*, thereby accelerating tumorigenesis [125]. Meanwhile, these mutations in oncogenes and tumor suppressor genes in tumor cells may alter metabolic signaling pathways, producing ROS accumulation [126–128]. For instance, the oncogenic mutation of MYC and KRAS can enhance glucose utilization and mitochondria-dependent macromolecule biosynthesis. This triggers the anaplerosis to supplement the substrates of the TCA cycle, which provides NADH to promote the mitochondrial electron transmission chain and ROS generation [128]. The inactivation of TP53 can weaken the cell’s antioxidative defense against ROS, further elevating the ROS burden. Additionally, HIF-1 (hypoxia-inducible factor 1) can be activated under hypoxic conditions, regulating glycolysis and mitochondrial function, and thus affecting ROS generation and accumulation [127]. This may create a vicious cycle, maintaining relatively high ROS levels favorable for tumor progression.

**Fig. 3:**
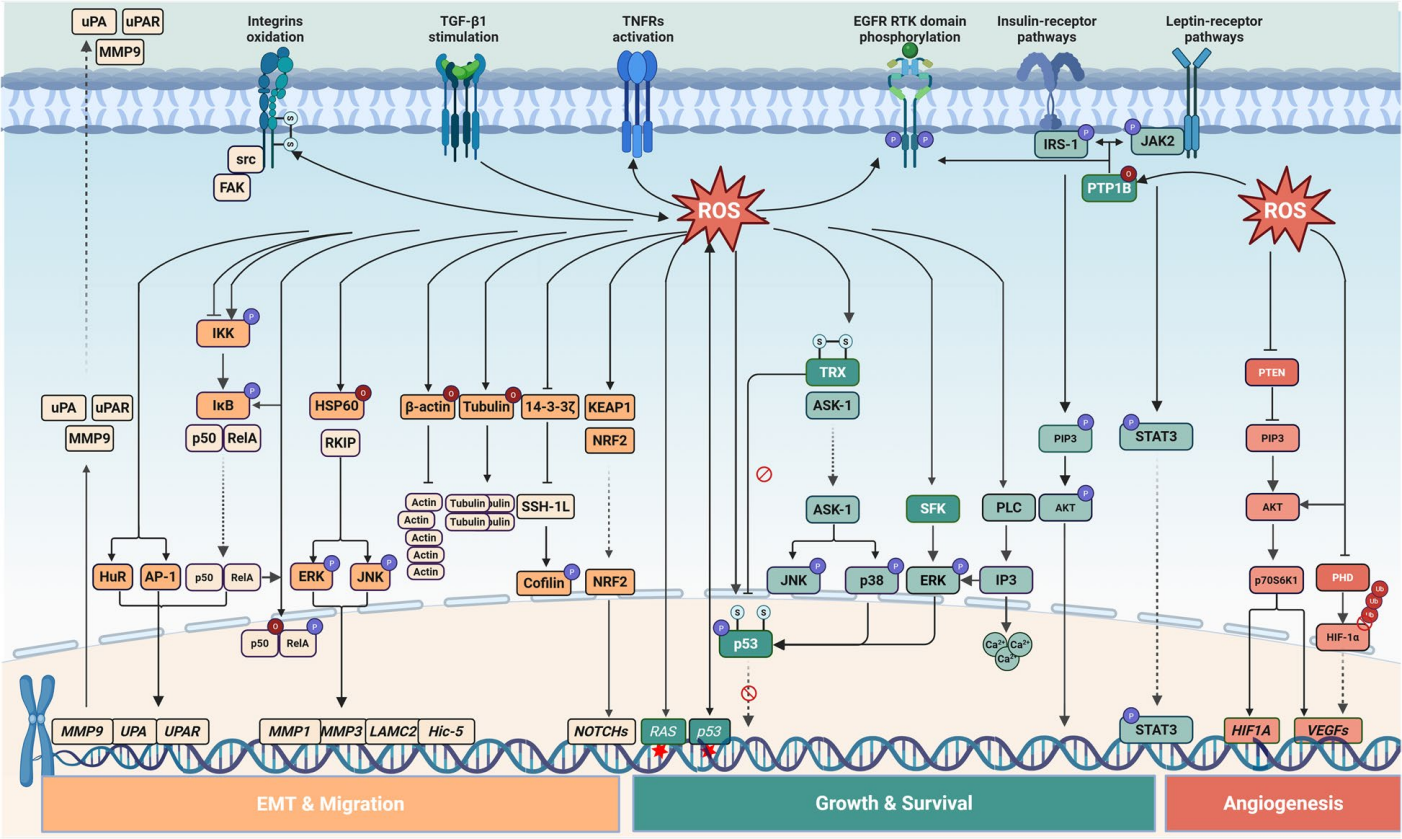
Mechanical map of the role of ROS in affecting the malignant phenotypes of tumor cells. ROS exerts dural interactions with cell receptor activation and regulates the intracellular signaling pathways to control tumor cell EMT, migration, growth, survival, and angiogenesis. Arrows, inhibitory arrows, and dashed arrows represent activating, inhibitory effects, and translocation, respectively. The red forbidden symbol indicates blockage. The spherical symbols labeled ‘P,’ ‘O,’ ‘Ub,’ and ‘S’ indicate phosphorylation, oxidation, ubiquitination, and thiol, respectively. Red star polygons indicate mutations. The figure was created with BioRender.com

The elevated ROS promotes the proliferation and survival of tumor cells (Fig. 3). ROS, a pivotal messenger, can activate the distinct signaling pathway via oxidation of specific amino acid residues, such as cysteine, in signaling proteins. H_2_O_2_ could oxidize the cysteine Cys215 in the catalytic domain of PTP1B (protein tyrosine phosphatase 1B), and the oxidized PTP1B promotes the clonal formation of hepatocellular carcinoma and epidermal cancer cells [129]. Moreover, the oxidized Cys215 in PTP1B has been found to cause significant conformational changes and block its substrate binding [130]. This might prevent the inhibition of the IRS-1-mediated PI3K/PDK1/AKT pathway and leptin-mediated JAK/STAT3, promoting cancer cell survival and proliferation [131, 132]. Similarly, H_2_O_2_-oxidized PTPs also prevent the dephosphorylation of RTK (receptor tyrosine kinase) of EGFR (epidermal growth factor receptor) [133], activating EGFR-mediated signaling transduction. In addition to receptor activation, ROS can activate certain SFKs (src family kinases) and the subsequent ERKs (extracellular signal-regulated kinases) pathway [134–136]. Mechanistically, the redox-dependent SFKs activation is possibly triggered by ROS-mediated polymerization through S–S bond formation or C-terminal cysteine oxidation [137]. Activation of the MAPK/ERK signaling pathway promotes cell proliferation and has an anti-apoptotic effect by influencing the activity of downstream cell cycle regulatory proteins and apoptosis-related proteins [138], and this process is facilitated by Trx-related ASK-1 regulation. ROS can directly activate the TNF (tumor necrosis factor) receptor and oxidize Trx, dissociating ASK-1 (apoptosis signal-regulating kinase 1) from Trx to phosphorylate and activate JNK (c-Jun NH_2_-terminal kinases) and p38 pathways [134–136]. Besides, increased H_2_O_2_ levels were associated with PLC (phospholipase C)-gamma tyrosine phosphorylation, activating PLC-gamma-mediated intracellular Ca^2+^ release and activation of pathways like ERK [135, 139]. Elevated ROS in tumor cells inhibits cancer-suppressive PTEN (phosphatase and tensin homolog) and activates the Akt (protein kinase B). This mechanism involves the PI3K (phosphoinositide 3-kinase)/AKT signaling pathway to alleviate damage and promote tumor growth [140, 141]. ROS could oxidize p50 and phosphorylate RelA (also known as transcription factor p65) to affect their DNA-binding capabilities, resulting in highly complex effects [84]. ROS mediates the phosphorylation of p53 via various protein kinases, including p38α MAPK, ATM (ataxia-telangiectasia mutated protein), and ERKs. These processes disrupt two cysteine clusters within the DNA-binding domain of human p53 that are critical for the specific binding of p53 to its consensus sequence, thereby impairing p53’s DNA-binding activity [91] and enhancing cell proliferation and survival [142, 143]. In summary, ROS has multiple upstream and downstream targets in these pathways. Their crosstalk might enable tumor cells to integrate the stress-adaptive pro-survival and pro-growth signals during tumor progression.

### ROS contributes to tumor cell angiogenesis, EMT, and metastasis

Angiogenesis is a significant hallmark of cancers that supports their progression. In the TME, ROS induces the expression of VEGFs (vascular endothelial growth factors) to activate tumor angiogenesis [143]. Elevated levels of NOX-derived ROS in ovarian cancer cells activate the HIF-1α/VEGF pathway [144]. Similarly, mutant p53 promotes ROS accumulation and activates the HIF-1α/VEGF pathway to induce angiogenesis in HCT116 human colon carcinoma cells [145]. In ovarian cancer, H_2_O_2_ is also essential in the EGF-induced PI3K/AKT/p70S6K1 pathway activation, which induces VEGF mRNA expression through HIF-1α activation [146]. It also oxidizes the ferrous ion of PHD (prolyl hydroxylase) [137] to inhibit its catalytic activity, thus stabilizing HIF-1α [147], indicating the important role of H_2_O_2_ in angiogenesis. Meanwhile, ROS can also stabilize HIF proteins independent of PHD, pVHL (von Hippel-Lindau protein), and p53 in kidney and liver cancer cells, but through neddylation of HIF-1α N-terminal site within amino acids 201 and 400 via NEDD8 [148]. However, increased angiogenesis was also observed in lung cancer cells after antioxidant treatment, for the reduced ROS increases BACH1 expression and promotes angiogenesis independent of HIF-1α, but rather in a BACH1-dependent manner [149].

Furthermore, elevated ROS can promote tumor metastasis. Tumor cell metastasis is a complex process that requires intracellular signaling and extracellular interactions. Tumor cell acquires the invasive capability through the EMT (epithelial-mesenchymal transition) process. EMT shifts tumor cells from epithelial-like to mesenchymal-like morphology and gene expression patterns [150] and interacts with redox homeostasis. TGF-β (transforming growth factor beta) is a classic EMT inducer [151]. TGF-β1 promotes the transcription of Notch4 and the activation of the Notch signaling pathway by activating the ROS/NRF2 pathway. The activated Notch signaling pathway then facilitates the development of EMT by directly activating the transcription of Snai1 [152]. In addition to NFR2/Notch axis, ROS also activates MAPK pathways to regulate tumor EMT [153–155]. The activated MAPK pathway increases the expression of transcription factors such as Snail and Slug, which play a crucial role in modulating the expression of genes associated with EMT, thereby facilitating the conversion of epithelial cells into mesenchymal cells [154].

The tumor cell with EMT could obtain various malignant features, including elevated integrin expression, enhanced cell motility, and increased extracellular degradation capability. The integrin family comprises transmembrane glycoproteins located on the cell membrane, consisting of α and β subunits. Multiple studies have demonstrated that integrins play a vital role in TGF-β-induced cancer cell EMT, migration, and invasion [156]. Recent studies have demonstrated that integrins could trigger the EMT-stimulating capability of both activated TGF-β1 and latent L-TGF-β1 [157, 158]. ROS has been found to upregulate the expression of integrins in TSCC (tongue squamous cell carcinoma), thus facilitating TSCC tumor cell EMT and metastasis [159]. Tumor cell motility relies on the dynamic remodeling of the cytoskeleton. ROS oxidizes cytoskeleton components β-actin and tubulin cysteine to slow actin polymerization and facilitate tubulin tetramer formation, respectively [160]. Meanwhile, high-level ROS alleviates 14-3-3ζ-induced inhibition of SSH-1L to enhance cytoskeletal extension, which includes cofilin phosphorylation and lamellipodia formation [160], increasing cytoskeleton remodeling. Moreover, increased expression of proteases enables tumor cells to degrade the extracellular matrix. The NOX-derived ROS could oxidize the cysteine of HSP60, the oxidized HSP60 could trigger the release of RKIP and subsequent ERK-JNK phosphorylation, activating the MAPK pathway and migration of hepatocellular carcinoma via MMP1, MMP3, LAMC2, and Hic-5 expression [161]. ROS also promotes HuR (human antigen R), NF-κB, and AP-1 nucleus translocation to increase uPA (urokinase-type plasminogen activator) and uPAR (urokinase-type plasminogen activator receptor) expression, degrading extracellular matrix to facilitate tumor cell metastases. Additionally, ROS-induced NF-κB [162], HIF-1α, and TGF-β [163, 164] pathways increased uPA and MMP-9 for extracellular matrix reshaping, affecting the integrity of intercellular connections in tumor cells and activating the PI3K/AKT pathway to promote tumor cell mobility [165, 166]. However, some studies have demonstrated that elevated ROS levels can trigger TP53 activity to inhibit melanoma and glioma cell metastasis by altering the expression of metastasis-related genes such as *MMP2*, *MMP9,* and *TWIST* [167, 168].

Moreover, antioxidants may promote tumor metastasis under certain circumstances, primarily by affecting the interaction between NRF2 and BACH1. Antioxidants such as vitamin C, vitamin E, and NAC (N-acetylcysteine) can alleviate oxidative stress by scavenging ROS within cells. However, this process may lead to a decrease in NRF2 activation, subsequently impacting BACH1 degradation and increasing BACH1 stability. Furthermore, BACH1 activates the transcription of HK2 (Hexokinase 2) and GAPDH (glyceraldehyde-3-phosphate dehydrogenase), enhancing glucose uptake, glycolytic flux, and lactate secretion, thereby promoting lung cancer metastasis through a glycolysis-dependent pathway [169–171]. This accumulating evidence implies that the role of ROS in tumor metastasis is contingent on pathophysiological conditions. ROS can also regulate metastasis by affecting the intercellular crosstalk in the TME [172, 173], which is further elaborated in the next section.

### Oxidative stress in tumor cell death and therapeutic resistance

Excessive ROS in tumor cells can cause DNA damage, lipid peroxidation, mitochondrial protein damage, and induce cell death [174]. For instance, GLS2 increases the generation of lipid ROS by converting glutamine to α-ketoglutarate, thereby inducing ferroptosis in hepatoma cells [175]. H_2_O_2_ treatment in prostate cancer cells activates TRPM2-Ca^2+^-CaMKII-mediated autophagy inhibition, leading to cell death [176]. However, apatinib-induced ROS/NRF2/p62 pathway could also trigger autophagy and apoptosis in lung cancer cells [177]. Similarly, quercetin 3-o-β-d-galactopyranoside induces apoptosis in breast cancer cells by triggering the ROS-mediated NF-κB signaling pathway [178]. These demonstrate that ROS is a critical mediator involved in drug-induced cytotoxic burden. ROS-targeting has been developed as a promising strategy to cause cell death, which will be discussed in detail in the subsequent section.

Tumor cells can counteract high ROS levels under oxidative stress with enhanced antioxidant synthesis or antioxidant enzyme expression [113], enabling tumor cell resistance to ROS-associated chemotherapy. Gastric cancer cells adopt PRDX2 (peroxiredoxin 2) to eliminate ROS and avoid apoptosis, acquiring resistance to cisplatin chemotherapy [179]. Also, overexpressed ROS-scavenger aldehyde dehydrogenase was observed in crizotinib-tolerant gastric cancer cells and was further shown to promote chemotherapeutic resistance in lung, breast, and colon cancer cells [180]. Recent studies have revealed that MFSD12 (major facilitator superfamily domain containing 12) is overexpressed in various cancers and promotes cystine storage in lysosomes, which can effectively buffer GSH depletion and tumor cell damage, leading to chemotherapy resistance in patients with breast cancer [181]. DDRGK1 (DDRGK domain-containing protein 1) [182] and iASPP (inhibitor of Apoptosis Stimulating Protein of p53) [183] can directly competitively bind to KEAP1, inhibiting the ubiquitin–proteasome-mediated degradation of NRF2 to resist ROS, promote cell growth, and inhibit chemotherapy-induced apoptosis. Furthermore, high ROS concentrations can activate the Aryl hydrocarbon receptor, allowing it to bind to PPP1R3C via sulfenylation, thereby enhancing glycogenolysis, PPP, and subsequent NADPH generation to achieve chemotherapy resistance [184]. These studies indicate that tumor cells possess various mechanisms to maintain redox homeostasis under chemotherapy-induced high ROS pressure, strengthening their therapeutic resistance capabilities.

Radiotherapy is also a critical therapeutic approach for many malignant cancers, and increasing evidence suggests that ionizing radiation exerts its antitumor effects by inducing the production of ROS, triggering cell death, such as lipid peroxidation-mediated ferroptosis [185] and immunogenic cell death [186] beyond the DNA damage effects. However, cancer cells have evolved adaptive mechanisms to counteract ROS damage, leading to resistance to radiotherapy. GBM (Glioblastoma) is the most common primary malignant tumor in the central nervous system. However, due to the therapeutic resistance mediated by GSCs (glioma stem cells), the standard non-surgical treatments such as radiotherapy and chemotherapy provide limited benefits [187]. Experiments have shown that the anti-proliferative protein PHB(prohibitin) is upregulated in GSCs, and it binds to the mitochondria-specific PRDX3 (peroxidase peroxiredoxin 3), stabilizing the PRDX3 protein through the ubiquitin–proteasome pathway [187]. PRDX3 then reduces peroxide levels and protects cells from oxidative damage. Therefore, upregulated PHB in GSCs protects them from ionizing radiation-induced oxidative damage and promotes GBM radiotherapeutic resistance through PRDX3-mediated ROS scavenging [187]. Radiotherapy is also critical in hepatocellular carcinoma control, but dysregulation of the redox system can lead to radioresistance. The radiotherapeutic resistance of hepatocellular carcinoma cells could be enhanced by NUPR1 (nuclear protein 1) expression [188]. The interaction between NUPR1 and AhR (aryl hydrocarbon receptor) has been found to facilitate the degradation of AhR. This reduces AhR nuclear translocation via the autophagy-lysosome pathway, thereby attenuating CYPs-mediated ROS generation and promoting radioresistance in HCC (hepatocellular carcinoma) [188]. Additionally, radioresistance is a major obstacle to advanced head and neck squamous cell carcinoma treatment. Studies have shown that UBE2C (ubiquitin-conjugating enzyme E2C) may be related to radioresistance, potentially regulating radioresistance through ROS signaling [189]. However, the mechanisms of UBE2C-mediated radioresistance remain unclear and warrant further experimental investigation [189]. In NSCLC (non-small cell lung cancer), microRNA-139 (miR-139) was found to be a novel radiosensitizer that functions by inhibiting NRF2 signaling [185]. In summary, tumor cells can utilize multiple pathways to reduce ROS burden and thus facilitate radiotherapy resistance. Sensitizing tumor cells to ROS damage is of great significance for overcoming radiotherapy resistance.

The above evidence implies that tumor cells harbor complex crosstalk between ROS and oncogenic signaling pathways, and this contributes to the tumor progression under a favorable ROS-rich condition. Notably, tumor cells also develop an adaptive antioxidant network to defend against cytotoxic ROS damage from either rapid cell metabolism or ROS-inducing therapies.

## The effects of ROS and adaptive responses to oxidative stress on the TME

**Fig. 4:**
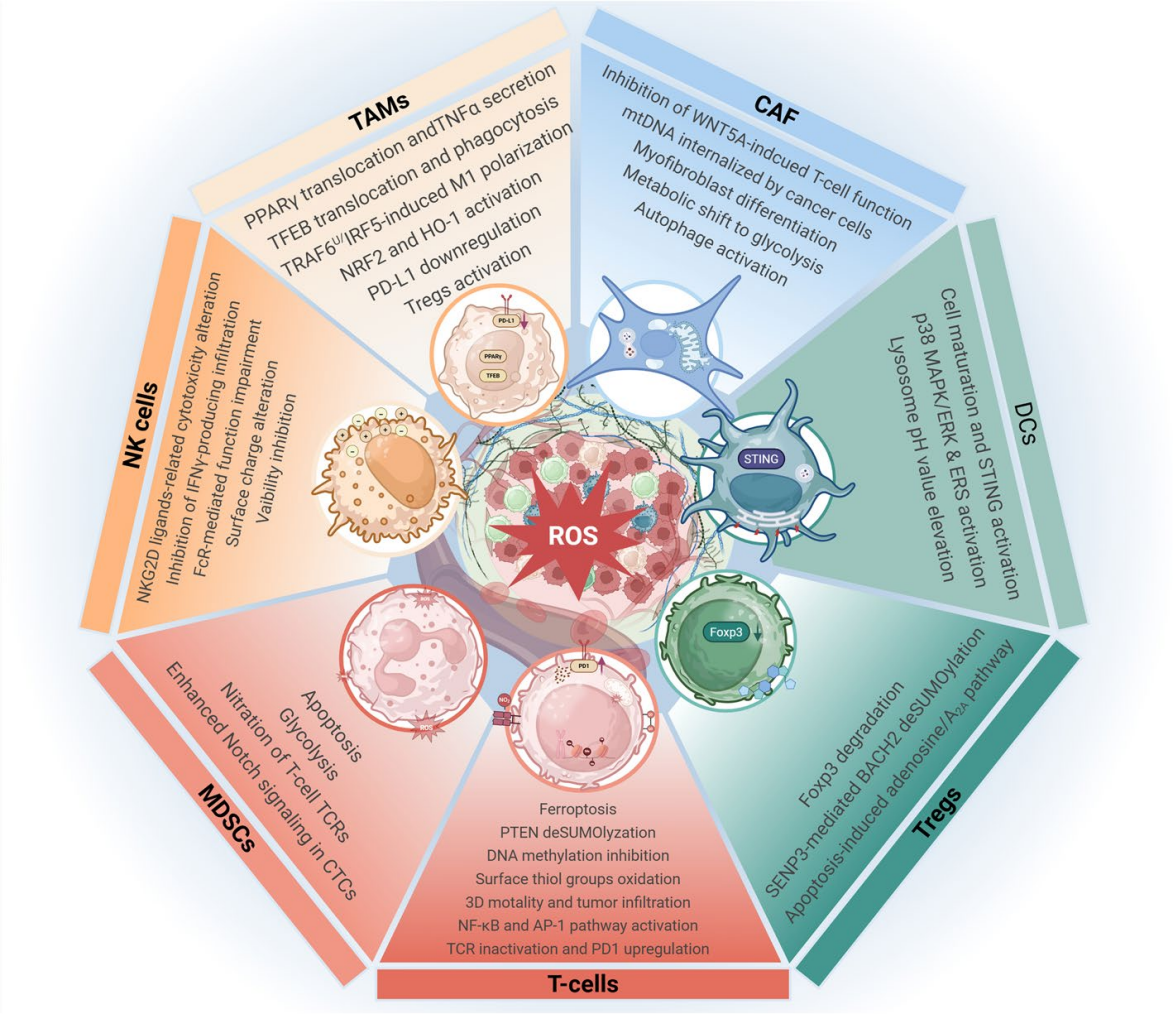
The graphical summary of the effects of ROS on TME cells. ROS generated in the TME exerts multiple roles in regulating DCs, T-cells, Tregs, MDSCs, NK cells, TAMs, and CAFs to affect TME immunosuppression. ROS, reactive oxygen species. TME, tumor microenvironment. DCs, dendritic cells. Treg, regulatory T-cells. MDSCs, myeloid-derived suppressor cells. NK cells, natural killer cells. CAFs, cancer-associated fibroblasts. The figure was created using BioRender.com

In addition to tumor cell regulation, ROS and adaptive responses to oxidative stress play an important role in the interaction between tumor cells and their microenvironment, significantly contributing to tumor progression (Fig. 4) [190].

### Oxidative stress is involved in immunosuppressive cell regulation

Immunosuppression is a critical hallmark in cancers [191]. The main cell components that contribute to TME immunosuppression include CAFs, MDSCs, TAMs, and Treg (regulatory T-cells) [192–194], which were highly plastic during cancer progression and could be recruited in the TME through abnormal ROS levels, causing immunosuppressive phenotype shift of tumors.

CAFs are a phenotypically and functionally heterogeneous population of mesenchymal cells highly engaged in intercellular crosstalk in the TME. They could remodel the ECM (extracellular matrix) to promote tumor growth, angiogenesis, metastasis, invasion, and even therapeutic resistance, and their pro-tumor effects are highly associated with ROS regulation. CAFs exhibit a myofibroblast-like phenotype and constitute critical stromal components of aggressive tumors. JunD-deficiency-derived ROS elevation enhances HIF-1α accumulation and stimulates the CXCL12/CXCR4 pathway, which activates RhoA-GTPase to promote myofibroblast differentiation in TME, facilitating tumor metastasis [195]. A recent study discovered that MAOA (monoamine oxidase A) in stromal cells promotes their myCAF conversion, and MAOA inhibition prevented intracellular ROS accumulation, boosting WNT5A secretion to enhance the Ca^2+^-NFATC1 pathway in CD8 + T-cells, thus suppressing prostate cancer growth. However, the mechanism of ROS-mediated WNT5A secretion remains unclear [196]. Besides, the ROS generated by tumor cells can enhance the autophagy of CAFs, providing various nutrients for tumor cells [197]. ROS can also promote mitophagy and the release of mtDNA (mitochondrial DNA) from CAFs, and the mtDNA could be internalized by lung and breast cancer cells to promote their survival and metastasis [197]. Meanwhile, CAFs also affect tumor cell redox homeostasis. CAFs-secreted metalloproteases can trigger the Rac1b/COX-2-mediated ROS release in tumor cells, leading to tumor cell EMT and stem cell characteristics acquisition [198]. In addition, CAFs can reduce lipid-ROS accumulation in gastric and prostate cancer cells via exosomes, allowing them to acquire chemotherapy resistance characteristics [199]. This suggests that tumor cells can reduce ROS burden by relying not only on their own antioxidant system but also on the involvement of non-tumor cell crosstalk.

MDSCs are a group of immunosuppressive myeloid cells. It is a precursor to DCs (dendritic cells), macrophages, and/or granulocytes, significantly suppressing immune cell function [200, 201]. Pathological expansion of MDSCs can trigger activation of the JAK/STAT3 (janus kinase/signal transducers and activators of transcription 3) pathway and NOXs, increasing ROS generation [201]. Accumulated ROS was also observed in human NSCLC MDSCs with downregulated LAL (Lysosomal acid lipase). LAL deficiency increased MDSC glycolysis and doubled ROS levels compared to those in LAL +/+ MDSCs [202]. ROS overload has been reported to contribute to MDSC apoptosis. The deficiency of NRF2 could increase ROS levels and sensitize the tumor-circulating or bone marrow MDSCs to apoptosis [203]. However, ROS-induced glycolysis in MDSCs appears to function as a feedback brake that curbs excessive ROS accumulation, thereby suppressing apoptosis. Glycolytic intermediates such as phosphoenolpyruvate possess antioxidative properties that mitigate excessive ROS generation. By sustaining optimal ROS levels, MDSCs are shielded from ROS-induced apoptosis, thereby promoting their expansion and accumulation in tumors [204]. Nonetheless, ONOO^−^ produced by MDSCs can nitrate TCRs on T cells, thereby modifying their peptide-binding specificity and resulting in a diminished responsiveness to antigen-specific stimulation [201, 205]. The ROS produced by MDSCs can also promote the proliferation and metastasis of CTCs (circulating tumor cells) via the NRF2/Notch1/Nodal signaling pathway. Specifically, the increased ROS produced by PMN-MDSCs upregulates Notch1 in CTCs through the ROS-NRF2-ARE axis, enabling CTCs to respond to ligand (Jagged1)-mediated and PMN-MDSC-driven Notch activation [206]. In summary, the MDSC-derived ROS signal is critical in promoting tumor cell metastasis and T-cell immunosuppression, contributing to tumor progression. Instead, inhibiting ROS generation can reduce the immunosuppressive effects of MDSCs in vitro [201, 205] and restore the proliferation of CD8 + T-cells [207].

TAMs are infiltrating macrophages in tumor tissues, mainly differentiated from monocytes. Extrinsic induction of ROS overload could promote the pro-inflammatory conversion of TAM. ROS elevation forced by a Cu_2_-_x_Se nanoparticle could induce the ubiquitination-mediated TRAF6 degradation, and this triggered IRF5-mediated increase of CD80 and CD86 and suppression of CD206 and Arg1 expression, converting TMA from TM2-like to M1-like phenotype [208]. Vinblastine was reported to reset TAM towards the M1 phenotype. Vinblastine activated phosphorylated NF-κB and p22^phox^ protein, and the activated p22^phox^-NOX2 complexes induced ROS generation, probably triggering ROS-dependent nuclear transcription factor EB (TFEB) nuclear translocation with enhanced phagocytic capacity [209]. However, the ROS-rich TME has been widely observed with few M1 macrophage infiltrations. The mechanism to explain the decreased M1 macrophages in ROS-rich TME might be attributed to the activated antioxidative response. The upregulated antioxidant gene expressions, including NRF2 and HO-1, are associated with attenuated M1 and enhanced M2 polarization macrophages in colorectal cancer [210]. Given the role of HO-1/BACH1/NRF2 axis in antioxidant response (Fig. 2), the upregulated NRF2 and HO-1 might limit macrophage ROS generation to reach pro-inflammatory levels, blocking M1 phenotype conversion. Besides, the ROS changes in tumor cells contribute to the TAM-mediated immunosuppression in TME. ROS can recruit TAMs into tumor tissues and enhance their M2 polarization [24–26]. Recent studies have indicated that eliminating ROS in ovarian cancer cells promotes the secretion of exosome-derived miR-155-5p, and this downregulates PD-L1 (programmed death-ligand 1) expression in macrophages [211]. Also, ROS can enhance TAM PPARγ nuclear translocation to induce TNF-α release, thereby promoting tumor cell invasion [212]. Meanwhile, macrophage-induced Treg cell is ROS-dependent [27].

In Treg cells, ROS regulate transcription factor activities to affect their immune functions. transcriptional repressor SENP3 (SUMO1/sentrin/SMT3 specific peptidase 3) has been found to positively regulate their suppressive functions. ROS stabilizes SENP3 in Treg cells, thereby triggering BACH2 deSUMOylation. This mechanism modulates the nuclear localization and transcriptional activity of BACH2, ultimately maintaining the stability and suppressive functions of Treg cells [213]. ROS was also involved in TMED4-mediated Treg stability. TMED4 deficiency triggered HRD1/BIP-mediated ERAD (ER-associated degradation) of IRE1α, XBP1 level reduction, and NRF2 inhibition, leading to ROS accumulation. The increased ROS then decreased Foxp3 stability and attenuated Treg suppressive function with boosted anti-tumor immunity in TME [214]. Besides, in the TME, Tregs can secrete large amounts of antioxidants such as Trx, making Tregs less susceptible to ROS-induced apoptosis [215]. However, Treg cells can undergo apoptosis in an excessively high-ROS environment, reducing the efficacy of PD-L1 blockade and producing excessive adenosine, thereby promoting A_2A_-pathway-mediated immunosuppression [216]. These findings suggest that ROS plays critical roles in TMA-associated immunosuppression and malignant transformation.

### Oxidative stress affects the functions of anti-tumor immunocytes

Apart from pro-tumor immunosuppressive cells, multiple anti-tumor immunocytes in the TME rely on a specific ROS level to maintain their anti-tumor immune functions. Importantly, ROS plays a vital role in T-cell activation. T-cell activation is triggered by mitochondria-derived ROS [217]. Transient generation of a physiological level of ROS is indispensable for T-cell activation via ROS-dependent NF-κB- and AP-1-related pathways [21]. Meanwhile, a sustained mitochondrial ROS level is required for CD8 + T-cells’ 3D motility and infiltration into lung cancers [22]. CD8 + T-cells can sense ROS levels via SENP7 (SUMO-specific protease 7). ROS-triggered SENP7 in the cytoplasm can deSUMOylate PTEN protein to promote its degradation, thereby maintaining its metabolic state and anti-tumor function [218]. These discoveries suggest that a physiological ROS level is required to maintain T-cell anti-cancer immunological functions. Meanwhile, it has been found in mice that blocking the generation of extracellular superoxide does not impair T-cell proliferation or other functions [219]. However, the abnormal ROS elevation in the TME can affect the anti-cancer function of T-cells [220]. The increased ROS via primary bile acids accumulation can lead to CD8 + T-cell death [23]. Elevated ROS also promotes T-cell ferroptosis with increased ferrous uptake through the CD36-mediated p38-CEBPB-TfR1 axis [221]. The ferroptosis of CD8 + T-cells could be prevented by lipid ROS elimination via adenosine A2A receptor and GPX4 crosstalk [222]. When Treg cells contact CD8 + T-cells, they can release NOX2-containing microvesicles, increasing ROS levels and inhibiting CD8 + T-cell TCR activation [223]. ROS can also affect the redox status on T-cell surfaces, oxidize the cell surface thiol groups to S–S groups, thereby threatening T-cell survival, which is demonstrated in breast, colorectal cancer, and melanoma [224]. In renal cell carcinoma, CD8 + tumor-infiltrating lymphocytes have been found to produce increased ROS due to mitochondrial polarization and fragmentation, which decrease total DNA methylation and lead to activation defects that inhibit their anti-tumor functions [225]. In melanoma, TAMs-derived H_2_O_2_ reduces the activity of T-cells and NK (natural killer) cells [26]. Moreover, H_2_O_2_-treated microglia induce PD-1 (programmed cell death protein 1) expression in CD8 + T-cells [226], thereby promoting T cell functional inhibition and GBM progression. Since tumor-derived ROS also upregulate TAM PD-L1 expression [227], these results suggest that elevated ROS levels exhibit complex crosstalk with PD-1/PD-L-mediated immunosuppression in TME, escaping tumor cells from immune attack. This might be another reason, in addition to immunogenic death, to explain why oxidative-stress-damage-based nano-acoustic dynamic intervention could achieve positive results when combined with anti-PD-L1 immunotherapy [228]. Nevertheless, the specific role of ROS in T-cell activities was context-dependent and warrants wider validation to confirm its significance to immunotherapy in different cancers.

In NK cells, the elevated ROS in TME could change their surface charge to anionic, and this prevents their adhesion to target tumor cells [229]. Meanwhile, the MDSC-derived NO impaired the FcR-mediated functions of NK cells, which include antibody-dependent cellular cytotoxicity, cytokine release, and signal transduction, thereby attenuating monoclonal antibodies therapeutic efficacy [230]. Similarly, Galectin-3-induced ROS increase derived from neutrophils could decrease NK cell viability and promote high-grade serous carcinoma progression [231]. In melanoma, NOX2 knockout or inhibitor could increase the IFNγ-producing NK cell infiltration into lungs with reduced melanoma metastasis [232]. As previously discussed, GSK-3β could promote NRF2 phosphorylation and ubiquitin-dependent degradation (Fig. 2). The inhibition of GSK-3β has been identified to increase breast cancer cell mitochondrial ROS, and this process decreases NKG2D ligands expression and suppresses NK cell function [233]. However, elevated NKG2D ligand expressions as well as increased susceptibility to NK cells were found to be probably induced by SFN- or IR-mediated ROS elevation in both breast and lung cancer cells [234], demonstrating the complex interaction between ROS and NK cell functioning.

ROS also contributes to DC cell activities. A Mn-LDH nanoparticle was deigned to deplete the intracellular GSH in DCs, and increased ROS promoted DC maturation through activation of PI3K/AKT, NF-κB, and STING (stimulator of interferon genes) pathways with the presence of Mn^2+^ [235]. Similarly, ROS in mouse DCs can promote SENP3 accumulation and deSUMOylate IFI204 (interferon-inducible protein), thereby activating their STING signaling and initiating anti-tumor immune responses [236]. ROS also activates p38 MAPK/ERK pathways and ERS (endoplasmic reticulum stress) to enhance DC maturation and increase lysosomal pH to maintain antigen conservation for antigen cross-presentation [237], thereby facilitating T-cell activation.

## Antioxidative treatment to control cancer progression via ROS scavenging

**Fig. 5:**
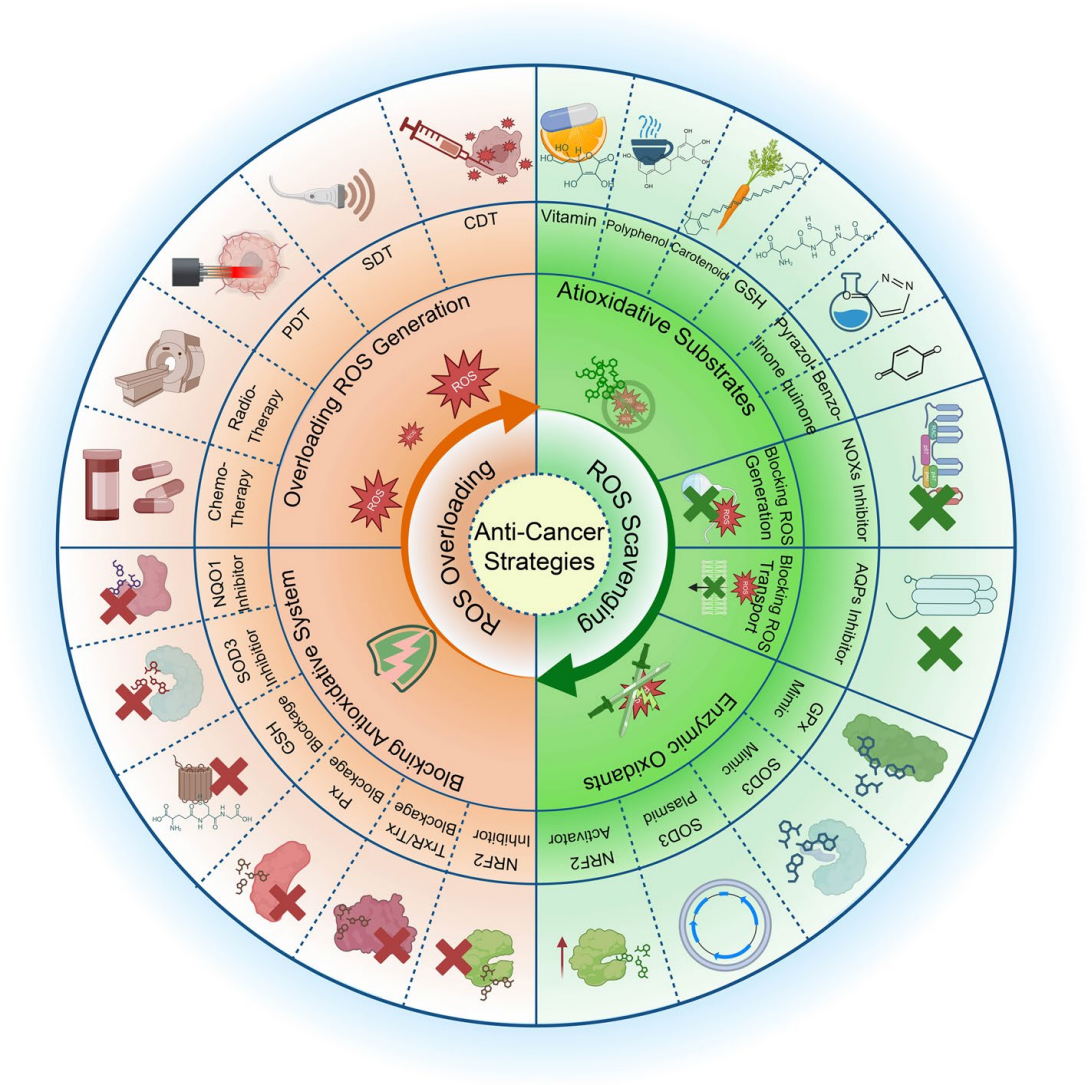
Summary of oxidative stress-based strategies for cancer therapy. Current therapeutic strategies to control ROS can be divided into ROS overloading and scavenging approaches. The ROS-scavenging approach includes the addition of antioxidative substrates, enzymatic oxidants, and blockage of ROS generation. In contrast, the ROS overloading approach involves intracellular antioxidative system blockage and ROS overloading. ROS, reactive oxygen species. PDT, photodynamic therapy. SDT, sonodynamic therapy. CDT, chemodynamic therapy. The figure was created using BioRender.com

Given that ROS plays a significant role in tumorigenesis and progression, applying antioxidants to prevent cancer is appealing [238]. Nevertheless, the cancer-preventive or therapeutic potential of antioxidants remains controversial due to conflicting research outcomes. Though some studies suggest the anti-cancer roles, other research has presented the pro-cancer risk, such as tumor metastasis [239]. Furthermore, their role in chemotherapy remains controversial [240–242]. These discrepancies may stem from various factors, including variability in antioxidant types, dosage regimens, intracellular concentrations, experimental designs, and differential molecular mechanisms across biological systems. We summarize the current experimental evidence and clinical attempts to control tumors using different antioxidant strategies and better understand the involvement of oxidative stress in cancer prevention (Fig. 5).

### Targeting cancers through non-enzymatic antioxidants

Non-enzymatic antioxidants have shown some potential in cancer treatment by influencing oxidative stress levels and regulating the redox homeostasis within cells. Current non-enzymatic antioxidants applied in cancers include vitamins, carotenoids, polyphenols, pyrazolinones, GSH, and benzoquinone derivatives, with distinct antioxidative activities determined by their chemical structures.

Ascorbic acid (vitamin C), an essential water-soluble vitamin, exists in three forms in vivo: reduced form (ascorbic acid, ASA), the radical intermediate (ascorbyl radical), and the oxidized form (dehydroascorbic acid) (Table 1) [243]. ASA directly neutralizes various ROS, converting itself into stable ascorbyl radicals that can be converted back to ASA via enzymatic processes involving NADH/NADPH-dependent reductases [244]. Low ASA concentrations exhibit antioxidant effects, while high concentrations paradoxically promote oxidative stress. High ASA concentrations can be catalyzed by ferric ions (Fe^3+^) and cupric ions (Cu^2+^) into ascorbic acid-free radicals and ROS [245], increasing intracellular ROS levels and causing cancer cell damage [246–249]. Intravenous administration of vitamin C produces plasma concentrations hundreds of times higher than those produced by the maximum tolerated dose (MTD) of oral administration of vitamin C [247]. A pharmacokinetic study of intravenous vitamin C in 21 healthy volunteers and 12 cancer patients showed that vitamin C exhibited first-order kinetics at doses ≤ 75 g. At doses up to 100 g, it was primarily excreted renally within 24 h, with 99% clearance in healthy subjects and 89% in cancer patients [250] (NCT01833351). This trial and many others have shown that high doses of intravenous vitamin C (at millimolar levels) have an excellent safety profile [250, 251]. Other clinical studies have also demonstrated the safety of intravenous vitamin C when used as monotherapy or in combination with chemotherapy drugs [251–255]. In Chen, Q et al. ‘s report, they evaluated the efficacy of ascorbic acid using mouse models of ovarian cancer, pancreatic cancer, and GBM xenografts. Ascorbic acid was found to reduce tumor growth and improve prognosis significantly with pharmacological concentrations of vitamin C achieved through intravenous administration [256]. The peak concentration reached by single dose administration is more than 30 mM, which is a similar plasma concentration easily achieved when human intravenous vitamin C is injected. In vitro experiments showed that its Effective Concentration 50 (EC50) value on tumor cells is usually less than 10 mM, demonstrating a high potency of ascorbic acid [256]. Intravenous vitamin C administration also benefits electrothermic therapy in lung cancer patients. In a randomized Phase II clinical trial for patients with non-small cell lung cancer by Junwen Ou et al., one group (*n* = 49) received intravenous vitamin C combined with modulated electrothermic therapy plus best supportive care (BSC), and the other group (*n* = 48) received BSC alone. After three months of treatment, the disease control rate in the combination treatment group was 42.9%, compared to 16.7% in the control group [257], suggesting a positive result. For more evidence, other phase II clinical trials are underway (NCT02905578 and NCT04033107). However, a double-blind, controlled study conducted by the Mayo Clinic failed to produce positive results for oral ascorbic acid administration [258]. This study used oral vitamin C in 150 patients with advanced cancers, including colorectal cancer, stomach cancer, lung cancer, breast cancer, and pancreatic cancer. All patients were divided into two groups, with one group receiving vitamin C (10 g per day) and the other group receiving a lactose placebo [258]. The results do not suggest a benefit of oral vitamin C therapy [258], likely due to its inability to achieve pharmacologically effective plasma concentrations.

**Table 1:** The ROS scavenging strategies in cancer control

Categories	Drug names	Mechanism	ROS	Effects	Tumors	References
Vitamins	Ascorbic Acid (Oral vitamin C, low concentration)	ROS neutralization, antioxidant activation, and antioxidant gene expression upregulation	Decrease	No impact	Colorectal cancer, stomach cancer, lung cancer, breast cancer, and pancreatic cancer	[258]
				No impact	Lung cancer, liver cancer	[259]
				No impact	Breast cancer	[260]
				Pro-tumor	Lung cancer	[149]
	Ascorbic Acid (Intravenous Vitamin C, high concentration)	Metal-catalyzed free radical generation	Increase	Anti-tumor	Mouse liver cancer, human bladder cancer, prostate cancer, breast cancer, liver cancer, endometrial adenocarcinoma	[261]
					Moue ovarian cancer, pancreatic cancer, glioblastoma	[256]
					Human lymphoma, breast cancer, lung cancer, kidney cancer; mouse lung cancer, kidney cancer, colon cancer, melanoma	[262]
					Prostate cancer	[252]
					Pancreatic cancer	[254, 255, 263] NCT02905578
					Bladder cancer	NCT04046094
					Melanoma	[264]
					Multiple myeloma	[265]
	Vitamins A	Antioxidant gene expression upregulation, ROS neutralization	Decrease	No benefit	Lung cancer	[266]
				Pro-tumor	Non-melanoma skin cancer	[267]
				Anti-tumor	Breast caner	[268]
					Head and neck cancer, lung cancer	[269]
	Vitamins E	ROS neutralization	Decrease	Pro-tumor	Lung Cancer	[270]
				No benefit	Bladder, Breast, Colorectal, Esophagus, Lung, Oral and Pharynx, Ovarian, Pancreatic, Prostate, And Kidney Cancer	[271]
					Prostate Cancer	[272]
			Increase	Anti-tumor	Breast cancer	[273]
Carotenoids	Astaxanthin	Electron donation for free radical neutralization​	Decrease	Anti-tumor	nervous system, breast, and gastrointestinal cancers	[274]
	Lutein	ROS neutralization	Decrease	Anti-tumor	lung cancer and late age-related macular degeneration breast cancer	[275] [276]
	Lycopene	ROS neutralization	Decrease	Anti-tumor	Prostate, gastric, colorectal cancer	[277–279]
Polyphenols		ROS neutralization, ROS-producing enzyme inhibition, metal ions chelation, and NRF2 activation	Decrease	Anti-tumor	Pancreatic Cancer	[280]
	EGCG		Decrease	Anti-tumor	Lung cancer	[281, 282]
					Fibrosarcoma	[283–285]
	Hesperidin				Lung cancer	[286]
	Naringenin				Lung cancer	[287]
	Luteolin				Bladder cancer	[288]
					Colon cancer	[289]
					Lung cancer	[290]
	Apigenin				B-cell lymphoma	[291]
	Anthocyanins	Labile aroxyl radical formation​, NOX activation, mitochondria destruction, GSH depletion	Increase	Anti-tumor	B cell chronic lymphocytic leukaemia	[292]
					Colon cancer	[293]
	Emodin				Lung cancer	[294]
	Gingerol				Human glioblastoma	[295]
	Isoliquiritigenin				Cervical cancer	[296]
	EGCG				Colon cancer	[297]
	Hesperetin				Breast cancer	[298]
	Quercetin				Colon cancer	[299]
					Liver cancer	[300]
	Isoflavone daidzein				Breast cancer	[301]
	7,3′,4′-trihydroxyisoflavone				Cervical cancer	[302]
	Chlorogenic acid				Bcr-Abl(+) chronic myeloid leukemia	[303]
	Oligomeric proanthocyanidins				Ovarian cancer	[304]
	Hydroxychavicol				Prostate cancer	[305]
	Curcumin				Colon cancer	[306]
	Hydroxytyrosol				Prostate cancer	[307]
					Colon cancer	[308]
	Resveratrol				Lymphoma	[309]
GSH supplements	NAC	GSH generation	Decrease	Anti-tumor	Lung cancer	[310, 311]
					Triple-negative breast cancer	[312]
				Pro-tumor	Lung cancer	[270]
					Melanoma	[313]
Benzoquinones	MitoQ	Mitochondria targeting	Decrease	Anti-tumor	Breast and pancreatic cancer	[314]
			Decrease	No benefit	Melanoma and lung cancer	[315]
	SkQ1		Decrease	Anti-tumor	Fibrosarcoma and rhabdomyosarcoma	[316]
			NA	No benefit	Pancreatic cancer	[317]
NOX family Inhibitors	DPI	FAD complexation​	Decrease	Anti-tumor	Epithelial ovarian cancer	[318]
					Colon cancer	[319]
	GKT136901/GKT137831	NOX1,2,4,5 inhibiton	Decrease	Anti-tumor	Mouse melanoma, lewis lung cancer	[320]
		Unclear	Increase	Anti-tumor	Acute myeloid leukemia	[321]
SOD Mimics	MnP	H_2_O_2_ generation	Decrease	Anti-tumor	Breast cancer	[322]
					Clear-cell renal carcinoma	[323]
NRF2 Activators	NDGA	ROS-damaged phenylalanine scavenging	Decrease	NA	Glioblastoma	[324]
		GPx and GR activity improvement	Decrease	Anti-tumor	Skin cancer	[325]
	Enzastaurin	GSK-3 phosphorylation inhibition	NA	Anti-tumor	Colon cancer and glioblastoma	[326]
	Poly-Nitroxide Albumin	H_2_O_2_ generation	Decrease	Anti-tumor	Triple negative breast cancer	[327]
	Omaveloxolone (RTA 408)	KEAP1-binding-mediated NRF2 stabilization​	Decrease	Anti-tumor	Squamous cell carcinomas	[328]
	DMF	Alkylation of KEAP1 cysteine residues and nuclear export of BACH1	Decrease	Anti-tumor	Pancreatic carcinoma Primary effusion lymphoma	[329] [330]
AQP3 Inhibitors	Auphen-derived organogold compounds	H_2_O_2_ influx reduction, intracellular ROS accumulation	Decrease	Anti-tumor	Melanoma	[331]
AQP7 Inhibitors	Z433927330	Endofacial AQP7 binding​	Decrease	Anti-tumor	Acute promyelocytic leukemia	[332]
AQP1 Inhibitors	AqB011	Intracellular loop D of AQP1 binding	Decrease	Anti-tumor	Colon cancer	[333]
	bacopaside II	Cell cycle arrest and apoptosis induction	Decrease	Anti-tumor	Colon cancer	[334]

*ROS* reactive oxygen species, *EGCG* epigallocatechin gallate, *NRF2* nuclear factor erythroid 2-related factor 2, *KEAP1* Kelch-like ECH-associated protein 1, *NOX* NADPH-oxidase, *GSH* glutathione, *NAC* N-acetylcysteine, *DPI* diphenyleneiodonium, *FAD* flavin adenine dinucleotide, *MnP* Mn porphyrin, *SOD* superoxide dismutase, *NDGA* nordihydroguaiaretic acid, *DMF* dimethyl fumarate, *AQP* aquaporin

Vitamin A is enzymatically converted from plant-based provitamin A carotenoids and animal-derived preformed vitamin A. Recent research suggests that preformed vitamin A exhibits direct antioxidant activity [335]. Its metabolite ATRA (all-trans retinoic acid) can activate gene expression, including *TRX* and *GCLC*, after binding to nuclear receptors, thereby influencing the Trx antioxidant system and GSH production [335]. Vitamin E can scavenge lipid peroxyl radicals, resulting in the formation of lipid hydroperoxides and vitamin E radicals. Vitamin E plays a role in protecting PUFAs in cell membranes. An animal study conducted by Pierpaoli et al. revealed that administering 100 mg/kg of annatto-T3 to HER-2/neu transgenic mice resulted in a delayed onset of mammary tumors, a reduction in both the number and volume of these tumors, and a decrease in the size of lung metastases [273]. Although the mechanisms of vitamins A and E are clarified, clinical investigations regarding the association between these vitamins and cancer occurrence are inconsistent [242, 267–269, 271, 272, 336] (NCT00006392). Unlike their theoretically desirable anti-tumor effects demonstrated in preclinical studies, vitamin A and E supplementations may paradoxically increase cancer risk in certain contexts according to several clinical reports [266, 267, 270]. Omenn et al. ‘s trial involved 18,314 smokers, former smokers, and asbestos-exposed workers, with the treatment group receiving a combination of 30 mg of beta-carotene and 25,000 IU of vitamin A per day [266]. The results showed that the relative risk of lung cancer incidence was 1.28 in the treatment group compared with the placebo group, and the overall mortality was 17%, suggesting that the combination of vitamin A and beta-carotene exhibited no preventive effect, but increased lung cancer incidence and death risk [266]. Similarly, in the study by Sayin et al., 0.1 g/kg or 0.5 g/kg of vitamin E was used on mice with lung cancer models induced by KRAS G12D and BRAF V600E mutations. The results showed reduced levels of ROS within tumor cells, but with significantly increased cancer progression and decreased survival [270]. In a randomized controlled trial by Zandwijk et al., no statistically significant difference in overall survival or event-free survival was observed between lung and head and neck cancer patients treated with vitamin A (300,000 IU daily for 1 year followed by 150,000 IU for the 2nd year) and the placebo group [269]. A prospective study by Lippman et al. using 400 IU/day of vitamin E in a relatively healthy male population after approximately five and a half years of intervention showed that this did not prevent prostate cancer and did not improve prognosis [272]. Consequently, a consensus on their preventive utility has not been reached, which could be attributed to their complex interaction with other molecules within the human body.

Carotenoids could facilitate the addition of lipid peroxyl radicals to the carotenoid polyene chain or the electron transfer from radical to the carotenoid polyene chain to scavenge ROS [337]. The carotenoids include alpha- and beta-carotene, lutein, astaxanthin, and lycopene. etc. For some tumors, such as bladder cancer and prostate cancer, the results with no association found between dietary intake of these carotenoids and decreased cancer risk were previously reported [338, 339]. Besides, an RCT (NCT00064298) enrolling 134 head and neck cancer patients with multiple FV concentrates diet showed that patients in the diet group had significantly higher serum lutein as well as α-carotene, β-carotene, but did not differ significantly in biomarkers of risk for developing second primary tumors [340]. However, recent studies have provided positive results regarding carotenoids’ role in cancer control. Astaxanthin is a natural C40 carotenoid and was reported to exhibit higher free radical inhibitory activity than α-tocopherol, α-carotene, β-carotene, lutein, and lycopene [341]. Copat et al. have reviewed the current research of astaxanthin in cancers, and many of the studies demonstrated its efficacy across distinct tumors, including nervous system, breast, and gastrointestinal cancers [274], though human-based evidence is lacking. In addition to astaxanthin, lutein exhibited a lower risk of developing lung cancer and late age-related macular degeneration compared to beta-carotene [275] and has shown inhibitory potential to breast cancer cells [276]. Lycopene is a natural carotenoid that has been discovered to exhibit anti-cancer efficacy, and it harbors higher antiproliferative effects on human cancer cells compared to other carotenoids, including alpha- and beta-carotene [342]. A systematic review summarizing the cancer incidence, improvement in treatment outcomes, and the mechanisms of lycopene action from 72 human and animal studies, and most of the in vivo anti-cancer effects were confirmed, with most of the research focusing on prostate cancers [343]. Previously, 79 patients with non-metastatic prostate cancer enrolled in an RCT (NCT00433797) were tested by nutritional intervention with tomato products containing 30 mg lycopene per day or other components. As a result, patients with the highest elevation in lycopene alone exhibited decreased PSA [277], indicating their inhibitory effects on prostate cancer progression. Meanwhile, a recent phase I trial (NCT0149519) involving 24 cases was conducted to evaluate the MTD, safety, pharmacokinetics, and effects on IGF-1 signaling and angiogenesis of synthetic lycopene in metastatic prostate cancer patients, the results indicate that the synthetic lycopene has significant effects on angiogenesis and IGF-1 signaling with low toxicity (pulmonary embolus in one out of 12 participants) [344]. In addition to prostate cancers, Lycopene also presents therapeutic potential in gastrointestinal tumors, including gastric cancer [278] and colorectal cancer [279], implying its promising potential in cancer control.

Polyphenols harbor distinct chemical structures, but contain at least one aromatic ring and hydroxyl group [345], exhibiting strong antioxidant properties and can be divided into non-flavonoids and flavonoids [346]. A widely reported non-flavonoid polyphenol is resveratrol. Resveratrol is a natural compound that exists in many foods and exerts an antioxidant bioactivity. Its anti-cancer roles have been widely investigated and confirmed in more than ten cancer types [347]. Current clinical evaluation of resveratrol intervention on cancers has obtained periodic results. A phase I RCT (NCT00920803) enrolls 9 participants [348] with colorectal cancer and hepatic metastases, who received oral administration of resveratrol at 5.0 g once daily for 14 days, and has been completed. This study found that resveratrol was well tolerated and it increased cleaved caspase-3 by 39% in malignant tissues compared to placebo-treated patients, suggesting it promotes colorectal cancer cell death. In breast cancers, a pilot phase I trial studied the role in of resveratrol in systemic sex steroid hormones of postmenopausal women with BMI ≥ 25 kg/m2 (NCT01370889), the results showed that resveratrol did not affect serum concentrations of estradiol, estrone, and testosterone but increased the concentrations of sex steroid hormone binding globulin (SHBG) by in 10% average [349], indicating its SHBG-associated anti-tumor potential. However, negative results have also been observed. A phase I pilot clinical trial enrolling 11 colon cancer patients demonstrated that oral intake of resveratrol at 80 mg per day did not inhibit Wnt pathway in colon cancers (NCT00256334) [350]. Currently, the data from more completed or ongoing trials have not been posted, and they are expected to provide a robust evaluation of resveratrol’s real therapeutic effects on cancers in the future. Flavonoids harbor a C6-C3-C6 skeleton. Different substituents on the carbon skeleton exhibit distinct antioxidative properties. The hydroxyl groups on the phenyl ring react with free radicals to generate relatively stable flavonoid radicals [351]. Flavonoids can bind to the amino acid residues of xanthine oxidase via hydrogen bonds and affect NOX enzyme activity via hydroxyl group and double bond, thereby inhibiting ROS-producing enzymes [351]. Besides, flavonoids could chelate metal ions to prevent the Fenton reaction and disrupt the binding of KEAP1 and NRF2 to promote antioxidant gene transcription [351]. Notably, whether polyphenols exhibit anti-oxidant or pro-oxidant activity depends on factors including chemical properties, cell type, and oxidative stress level; in some cases, polyphenols exert pro-oxidative effects. They can react with metal cations to form labile peroxyl radicals, a type of radical with an oxygen atom attached to an aromatic ring [295]. Aroxil radicals can react with oxygen to form O_2_•^−^ [295]. Compounds, such as ferulic acid and apigenin, can also activate NOXs to generate ROS inside the cells [295]**.** These pro-oxidant effects may have anti-cancer properties. In a study by Suganuma et al., (−)-epicatechin administration with 100 μM EGCG ([3H](−)-epigallocatechin gallate) incorporated significantly induced apoptosis of human lung adenocarcinoma cell PC-9, suggesting that EGCG may serve as a potential anti-lung cancer therapeutic agent [281]. Previous studies have indicated that polyphenols can potentially exert protective effects against cancer, while further clinical research is warranted to confirm their pharmacokinetics and efficacy [280, 351–354].

Pyrazolinone-based synthetic small molecules also contribute to ROS scavenging as their antioxidant potential was comprehensively assessed [355]. The pyrazolinones reported in cancer research include antipyrine and edaravone. Antipyrine derivatives have shown antioxidant and anti-tumor effects on Ehrlich ascites carcinoma cells [356]. Edaravone has been approved for the treatment of amyotrophic lateral sclerosis and acute ischemic stroke. The equilibrium between the neutral and anionic forms of edaravone underlies its antioxidant activity in vivo. The edaravone anion transfers an electron to •OH, converting them into corresponding anions and terminating free radical chain reactions [357]. Additionally, edaravone significantly prevents mitochondrial oxidative damage [358] and activates the NRF2-ARE pathway to upregulate the expression of multiple antioxidant enzymes [359]. While some early in vitro and in vivo assays suggested potential anticancer activity, recent studies demonstrate no significant cytotoxicity of edaravone against MCF-7 and HT-29 cancer cell lines [360]. Although exhibiting limited anticancer efficacy, edaravone’s antioxidant and anti-inflammatory properties make it suitable for protecting normal cells from the adverse effects of chemotherapy, radiotherapy, or immunotherapy [361]. Notably, edaravone reacts with pterin derivatives to form a substance that undergoes rapid reaction with NADH, generating ROS and inducing cell death [362].

GSH is an effective antioxidant that plays a bidirectional role in cancer development. It rapidly detoxifies oxidative stress. The clinical applications of GSH are limited due to the difficulty in crossing cell membranes [363]. Cys is a substrate for GSH synthesis, but its toxicity prevents its use in humans [363, 364]. NAC, a precursor of Cys, was synthesized with low toxicity. NAC’s cellular protective effects have been observed in vitro, where NAC serves as a source of Cys for GSH synthesis, thereby increasing intracellular GSH generation [364]. Some studies do suggest that NAC can inhibit the proliferation of tumor cells [310–312]. However, frustrating results of NAC have also been reported. In Conaway et al. ‘s study, NAC was incorporated into the diet of A/J mice at concentrations of 160 and 80 mmol/kg to evaluate its preventive potential against 4-(methylnitrosamine)-1-(3-pyridyl)-1-butanone (NNK)-induced lung tumors [311]. The results showed that at the higher dose (0.8 MTD, 160 mmol/kg diet), NAC significantly delayed the malignant progression of lung tumors from adenoma to adenocarcinoma in NNK-treated mice, as evidenced by the reduced incidence of adenocarcinomas and corresponding increase in adenomas at 52 weeks. However, NAC did not significantly inhibit the overall incidence and multiplicity of lung tumors [311]. Moreover, Volkan et al. reported that diet supplementation with NAC increases tumor progression and reduces survival in mice with lung cancer induced by B-RAF and K-RAS. Elevated GSH expression in cancer tissues alleviates tumor oxidative stress and sustains tumor growth; therefore, its anti-cancer effects have not been confirmed [270].

Benzoquinone is a natural compound with special carbonyl structures that make it easily reduced, and its derivatives exert antioxidant activities in human disease [365]. Distinct benzoquinone derivatives have been applied in cancer intervention, including CoQ10, MitoQ, and SKQs. CoQ10, also known as ubiquinone, is a benzoquinone-containing antioxidant. Though CoQ10 decrease has been found in cancers, the efficacy of CoQ10 supplementation alone in human cancer control via antioxidation has not been widely reported. The MitoQ is a decyl-triphenylphosphonium-cation (TPP)-linked CoQ10 with enhanced mitochondrial targeting capabilities. Notably, MitoQ is the first mitochondria-targeting agent entering clinical trials, and the safety of MitoQ administration has been verified [366], suggesting its potential in cancer treatment. Oral administration of MitoQ could decrease mitochondrial ROS and inhibit the growth and metastasis of human breast [367] and pancreatic cancer cells [314]. However, intraperitoneal injection of MitoQ did not affect BRAF-driven melanoma nad KRAS-driven lung cancer development [315]. Plastoquinone derivatives (SkQs) were reported to harbor a larger “window” between anti- and pro-oxidant concentration (higher concentration) than MitoQ, meaning that SkQs may exert stronger antioxidant effects [368]. SkQ1 is one of the active SkQs, and the nanomolar concentrations of SkQ1 treatment suppressed fibrosarcoma and rhabdomyosarcoma tumor cell growth via antioxidant action [316]. However, the insufficient improvement of oncological parameters and survival of PDAC-bearing mice after SkQ1 treatment has also been reported [317].

Although some research suggests that the non-enzymatic antioxidants may have a therapeutic effect on certain cancer types, such as lycopene for prostate cancer. However, more exciting results from clinical trials are still lacking, indicating that the current understanding of their roles in the human body is limited. The development of novel antioxidants in cancer control and detailed evaluation of their efficacy and safety are expected. In addition, more clinical trials are needed to validate the pharmacokinetic properties of current antioxidants, the optimal route of administration, and their potential synergies with existing therapies to better understand their role and potential in cancer treatment.

### Combating cancers by targeting ROS-generating enzymes

Given the pivotal role that ROS plays in cancer progression, attempts to target ROS generation to repress tumor growth have been initiated. NADPH oxidases are comprised of seven members, including NOX1–5 and dual oxidases 1 and 2. These ROS-generating enzymes have been employed as the main targets for tumor control. Diphenyleneiodonium was the first non-selective NOX inhibitor discovered. It forms a relatively stable covalent adduct by interacting with FAD, inhibiting electron transfer from NADPH to molecular oxygen [369], thus reducing ROS generation and exhibiting anti-cancer effects in colon and epithelial ovarian cancer cells [318, 319]. Pyrazolopyridine dione derivatives, including GKT136901 and GKT137831, function as dual inhibitors targeting the NOX1/4 isoforms, while exhibiting reduced efficacy against NOX2 and NOX5, as indicated by IC50 values of approximately 100–200 nM for NOX1/4 and greater than 1 μM for NOX2/5. Notably, GKT136901 demonstrates a tenfold selectivity for NOX1/4 over NOX2/5. In contrast, GKT137831 is characterized by enhanced pharmacokinetic properties while maintaining effective NOX1/4 inhibition [370]. Preclinical investigations have highlighted their therapeutic potential in attenuating tumor growth, particularly in models of KRAS-driven pancreatic and colorectal cancers, by disrupting ROS-mediated pro-survival signaling pathways [371]. However, these NOX inhibitors lack target specificity, and their precise pharmacological effects remain to be elucidated [372].

### Employment of NRF2-AREs-antioxidant axis and enzymic antioxidation for cancer control

As previously discussed, the transcription factor NRF2 triggers antioxidant gene expression and combats oxidative stress. The main mechanism of NRF2 activators is to block the binding of NRF2 and KEAP1 or reduce the level of KEAP1, weakening KEAP1’s pro-degradation effect on NRF2, thereby promoting the expression of various antioxidant genes. Several inhibitors blocking NRF2-KEAP1 binding have been discovered, but some have not yet been confirmed to exert their antitumor effects through the NRF2 pathway [73, 373, 374]. Several NRF2 activators activate NRF2 via various pathways, including nordihydroguaiaretic acid, enzastaurin, omaveloxolone, and dimethyl fumarate. NGAD (Nordihydroguaiaretic acid) inhibits GSK-3 phosphorylation at Ser9 and Thr390, and this prevents NRF2 degradation in a KEAP1-independent way [375]. NGAD demonstrated chemopreventive capability against skin cancer, as indicated by its mitigatory effects on TPA-induced mouse cutaneous oxidative stress [325]. The treatment of NGAD also reduced the quantity of ROS-damaged phenylalanine in GBM cells, showing a ROS scavenging potential [324]. A phase I dose-escalation trial (NCT00404248) on 35 patients with recurrent high-grade glioma has investigated the role of the NGAD derivative tetra-O-methyl nordihydroguaiaretic acid (TMNDGA) intravenous infusion. The results showed that a 1700 mg/day MTD, a 28% stable disease rate, and a 13% continuing treatment rate were obtained, suggesting its future study value [376]. However, a recent trial on 20 recurrent high-grade glioma patients with oral administration of TMNDGA demonstrated that increased dosage (up to 6000 mg/day) did not increase systemic exposure with a maximal AUC < 5 μg ∗ h/mL and was terminated prematurely (NCT02575794) [377], indicating the limited bioavailability of oral TMNDGA administration. Another inhibitor, enzastaurin, also exhibits inhibitory effects on GSK-3. Enzastaurin is a direct PKC-selective inhibitor and could target the Ser9 of GSK-3β as well and might prevent NRF2 degradation [326, 378]. Enzastaurin exhibits therapeutic effects against lymphoma (NCT03263026 and NCT01432951). A randomized, double-blind, phase III trial (NCT00332202) comparing enzastaurin with vehicle in patients with high-risk diffuse large B-cell lymphoma (DLBCL) who achieved remission after first-line treatment revealed that enzastaurin did not significantly improve disease-free survival in these patients after they attained a complete response to R-CHOP (C/EBP homologous protein) therapy [379]. As an approved agent for the treatment of Friedreich’s ataxia, omaveloxolone (RTA 408) is a semi-synthetic triterpenoid NRF2 activator whose anticancer activity has been demonstrated in multiple studies [328, 380]. At low concentrations, this triterpenoid binds to KEAP1 and reduces NRF2 degradation, which potentially reverses tumor immune evasion by decreasing ROS in the MDSC-related TME, thereby inhibiting tumor growth [381, 382]. At high concentrations, they suppress tumor growth by modulating signaling pathways such as NF-κB and JNK [381]. Dimethyl fumarate (DMF) and its metabolites activate NRF2 through alkylation of KEAP1 cysteine residues and nuclear export of BACH1, demonstrating efficacy across diverse cell types. Substantial evidence indicates DMF’s capacity to suppress proliferation and invasion in multiple cancer cell lines [329, 330, 383], while concurrently attenuating the pro-invasive function of TAMs within the TME [384]. Currently, DMF is approved for the treatment of psoriasis and multiple sclerosis, but its role in tumor therapy is subject to further clinical trial validation. Moreover, NRF2 activation enhances the anti-tumor activity of immune cells while enduring oxidative stress [385].

Abnormally downregulated antioxidant enzymes could promote cancer progression. In addition to activating the cellular antioxidant enzyme expression, endogenous or exogenous supplementation of antioxidant enzymes or enzymatic analogs is also considered a feasible strategy to inhibit tumor progression. Several synthetic SOD mimics have been developed, with metalloporphyrins gaining extensive research attention. For instance, Mn (manganese) porphyrin has been demonstrated to exert anti-tumor effects [322, 323] due to its ability to promote cancer cell apoptosis. But this was achieved by its ability to enhance intracellular H_2_O_2_ accumulation [386]. Messerli et al. used a macromolecular extracellular SOD3 mimic, poly(nitroxide)-albumin, to treat breast cancer and found that the intervention could reduce tumor ROS levels, inhibiting the proliferation and colony formation of highly metastatic 4T1 breast cancer cells. It also increases blood flow in the core region of breast cancer tumors and suppresses their lung metastasis [327]. Meanwhile, the development of SOD3-derived strategies can be used to mitigate treatment-associated complications following standard-of-care oncological interventions. Jang et al. found that increased SOD3 levels in cerebrospinal fluid by choroid plexus adeno-associated virus vector can combat lifelong neurological chemotherapy-related cognitive impairment, offering hope for alleviating chemotherapy complications and sequelae in cancer patients [387]. Greenberger et al. conducted a phase I-II clinical study and discovered that the oral administration of SOD3 plasmid/liposomes could control esophagitis during radiotherapy and chemotherapy for non-small cell lung cancer (NCT00618917). This suggests that SOD3, as a tumor treatment strategy, can simultaneously possess anti-tumor effects and control the complications following tumor treatment, thereby benefiting cancer patients.

In addition, there are other enzymatic antioxidants, such as GPx2 [388], that also show potential for tumor therapy. Although GPx2 expression is upregulated in a variety of cancers, it exhibits cancer-suppressing effects at different stages of cancer [389]. In the early stage of cancer, GPx2 can inhibit the expression of COX2 by removing the hydroperoxide required for COX2 activity and thus suppress the initiation of gastrointestinal tumors [390]. Banning et al. found that GPx2-knockdown cells have a higher ability to migrate and invade than GPx2-expressing controls [391], suggesting GPx2 has an anti-metastatic effect in advanced cancer. Ebselen is a GPx mimic developed for ROS reduction. It shows an inhibitory effect on lung cancer cells via GSH depletion, Notably, ebselen also targets the cys292 and cys361 of the autophagy protein ATG4B to suppress colorectal cancer progression [392]. Meanwhile, it eliminates dormant cancer stem cells and promotes CD8 + T-cell infiltration when combined with anti-PD-L1 therapy in esophageal cancers via Quiescent fibroblast-derived QSOX1 (quiescin sulfhydryl oxidase 1) inhibition [393]. Also, ebselen’s anti-tumor effects in vivo have been observed in pancreatic and renal cancer by binding to the c165 and c237 of QSOX1 [394]. These findings reveal ebselen as an efficient anti-cancer agent with pleiotropic capabilities. However, no clinical trial has been completed to validate its efficacy and safety on humans.

### Blocking transmembrane transport of ROS for cancer control

H_2_O_2_ exhibits complex crosstalk with oncological signals, and its transmembrane transport was facilitated by AQPs, which play a critical role in tumorigenesis. Therefore, AQP inhibitors have been developed for tumor control. The Auphen-derived organogold compounds, a class of AQP3 inhibitors, can markedly reduce H_2_O_2_ influx and intracellular ROS accumulation in melanoma cells, and this correlates with their impaired adhesion, proliferation, and migration [331]. Polymer-based nanoplatforms constructed from these compounds inhibit H_2_O_2_ transport in breast cancer cells, significantly suppressing tumor metastasis [395]. Notably, Auphen monotherapy increases lung metastasis, potentially through off-target AQP suppression in healthy tissues that systemically compromises metabolic coordination [396]. Z433927330 is a potent and selective AQP7 inhibitor [397]. It binds to the endofacial side of AQP7 to form hydrogen bonds with loop B backbone and Gln183 in transmembrane segment 4, thereby reducing leukemic cell (NB4 cell) proliferation [332]. AqB011 can selectively inhibit AQP1 and does not affect H_2_O transport at up to 200 μM [398]. Docking models reveal that AqB011 interacts with intracellular loop D of AQP1, which is a critical site for AQP1 ion channel blockade, significantly impairing migration of AQP1-positive human colon cancer cells, while the cell viability was not affected [333]. The clinically approved AQP1 inhibitor bacopaside II inhibits colon cancer growth via cell cycle arrest and apoptosis induction [334]. The combined therapy of AqB011 and bacopaside II demonstrates enhanced anti-migratory effects compared to monotherapy [399].

As mentioned above, ROS-targeted antioxidant therapy demonstrates complex and multifaceted potential in cancer prevention and treatment [346]. Non-enzymatic antioxidants, including vitamins, polyphenols, carotenoids, pyrazolinone, and GSH-related agents (e.g., NAC), have shown tumor growth inhibition capabilities in vitro and in animal models [250, 251, 256]. For instance, vitamin C significantly reduces tumor growth in ovarian, pancreatic, and glioma xenograft models, with its favorable safety profiles and synergistic effects with chemotherapy demonstrated in some clinical trials [250, 251, 256]. However, clinical studies on vitamin A and E have yielded less optimistic results, with some cases even showing increased cancer risk [266, 269]. Polyphenols display antioxidant and anti-cancer properties in laboratory studies, in which resveratrol inhibits malignant biomarkers in cancers such as colorectal and breast cancers, as clinically validated [348, 349]. Besides, the carotenoid lycopene has exhibited significant clinical effects in patients with both non-metastatic and metastatic prostate cancer as it reduced PSA, IGF-1 signals, and angiogenesis levels [277, 344]. However, limited promising results have been obtained for other antioxidant drugs.

Collectively, while numerous antioxidant agents show promising antitumor or preventive effects in preclinical studies, clinical trial outcomes remain inconsistent, suggesting deeper mechanistic insights await discovery. Notably, antioxidants can protect normal cells from oxidative stress-induced damage and mitigate treatment-related side effects, potentially representing a key future application direction [400, 401]. Although antioxidant therapy in cancer management currently lacks consensus, its clinical prospects remain considerable, particularly given its successful application in other diseases [402–404]. Significant interindividual variations in responses to oxidative stress and antioxidants necessitate the development of personalized assessment approaches for targeted therapies. Future research should focus on optimizing drug dosages, administration routes, and combination strategies with other therapies. There is an urgent need to develop antioxidants with enhanced targeting specificity, lower toxicity, and improved pharmacological profiles, coupled with well-designed clinical trials to validate their safety and efficacy in cancer treatment, ultimately providing more effective therapeutic options for cancer patients.

## Overloading ROS induces tumor cell damage and death

Although cancerous ROS levels may exhibit pro-tumoral effects, a high ROS level that exceeds a compensatory threshold could induce tumor cell death, thereby achieving an anti-tumor effect. One strategy to elevate intracellular ROS levels is to induce a burst in intracellular ROS load. Meanwhile, destroying tumor cell antioxidant capability through antioxidant enzymes or substrates targeting is also feasible to mediate ROS-related anti-cancer effects (Table 2).

**Table 2:** The ROS-overloading strategies in cancer control

Categories	Drug names	Mechanism	ROS	Effects	Tumors	References
ATF-6 inhibitors	Melatonin	RET induction	Increase	Anti-tumor	Head and neck squamous cell carcinoma	[405]
Complex I inhibitors	SMIP003	Complex I Q site inhibition	Increase	Anti-tumor	Triple-negative breast cancer	[406]
	Metformin	Complex I FMN site inhibition	Decrease	Anti-tumor	Colon cancer	[407]
	Phenformin	Complex I inhibition	Increase	Anti-tumor	Brain tumors	[408]
					Melanoma, breast, colon, lung, and prostate cancer	[409]
	Carboxyamidotriazole	Complex I inhibition	Increase	Anti-tumor	Colon cancer	[410]
DNA cross-linking agent	Cisplatin	MtDNA damage	Increase	Anti-tumor	Prostate cancer	[411]
		Mitochondrial content elevation	Increase	Anti-tumor	Ovarian cancer	[412]
​PML-RARα inhibitors	Arsenic trioxide	GSH depletion	Increase	Anti-tumor	Leukemia	[413]
		Complex IV inhibition	Increase	Anti-tumor	Leukemia	[414]
		TrxR and Prx inhibition	Increase	Anti-tumor	Breast cancer	[415]
Topoisomerase inhibitors	DOX	NADPH-to-DOX electron transfer	Increase	Anti-tumor	Breast cancer	[416]
		NOX indirect activation			Human osteosarcoma	[417]
					Leukemia	[418]
Nanoparticles	PTX@TPGS-PBTE NPs	ROS-targeted PTX delivery	Utilize ROS	Anti-tumor	Head and neck cancer	[419]
	Nanoparticles (CPT-Pt (IV) combined with P1 and mPEG2K-DSPE)	ROS-mediated cisplatin/camptothecin release	Utilize ROS	Anti-tumor	Colon cancer	[420]
	MOFs	GSH depletion	Increase	Anti-tumor	Liver cancer and breast cancer	[421]
Radiation therapy		Free electron-H_2_O interaction	Increase	Anti-tumor		[422]
PDT		Photosensitizer irradiation-induced ROS generation​	Increase	Anti-tumor		[423]
RDT		Photosensitizer irradiation-induced ROS generation via high-energy X-ray	Increase	Anti-tumor		[424]
SDT		Sonoexcitation and ultrasonic cavitation	Increase	Anti-tumor		[425, 426]
CDT		Fenton reactions	Increase	Anti-tumor		[427]
Xc- System inhibitors	Erastin	Xc- System blockade	Increase	Anti-tumor	Osteosarcom, fibrosarcoma, and lung cancer	[428]
		Induction of VDAC opening	Increase	Anti-tumor	Lung cancer	[429]
					Fibrosarcoma, Ewing’s sarcoma, neuroblastoma, adult acute monocytic leukemia	[430]
	IKE	Xc- system blockade	Increase	Anti-tumor	Diffuse large B-cell lymphoma	[431]
	Sorafenib	Xc- system blockade	Increase	Anti-tumor	Hepatocellular carcinoma and renal cell carcinoma	[432]
Cyst(e)inases		Blood cystine depletion	Increase	Anti-tumor	Prostate carcinoma and breast cancer	[433]
GCL inhibitors	BSO	GCL inhibition	Increase Increase	Anti-tumor	Renal cancer and ovarian cancer	[434]
CYP450 inhibitors	PEITC	GSH depletion	Increase	Anti-tumor	Breast cancer	[435]
					Leukemia	[436]
					Lung, colon, and ovarian cancer	[437]
	BITC				Non-small cell lung cancer cells	[438]
NRF2 inhibitors	Brusatol	NRF2 protein expression suppression	Increase	Anti-tumor	Pancreatic cancer	[439]
	Halofuginone	Global protein translation inhibition	Increase	Anti-tumor	Lung adenocarcinoma, Oesophageal cancer	[440]
	ML385	Neh1 domain of the NRF2 protein binding	Increase	Anti-tumor	Head and neck squamous cell carcinoma cancer cell	[441]
					Breast cancer	[442]
Trx/TrxR inhibitors	PX-12	Trx inhibition	Increase	Anti-tumor	Acute myeloid leukemia	[443]
	Ethaselen	Trx inhibition	Increase	Anti-tumor	NSCLC and gastric cancer cell	[444, 445]
	Piperine	TrxR Sec residue binding	Increase	Anti-tumor	Cervical cancer, Non-small cell lung cancer, hepatocellular liver carcinoma	[446]
	MGd	TrxR-targeting capability	Increase	Anti-tumor	Metastatic renal cell carcinoma	[447]
					Multiple myeloma	[448]
					Lymphoma	[449]
	Auranofin	TrxR-targeting capability	Increase	Anti-tumor	Human thyroid cancer cell	[450]
					Epithelial ovarian cancer cell	[451]
					Glioblastoma and NSCLC	[452]
					NSCLC	[453]
					Pancreatic cancer	[454]
					Chronic lymphocytic leukemia	[455]
Prx inhibitors	Celastrol	Prx-2 Cys172 residue binding	Increase	Anti-tumor	Gastric cancer	[456]
NQO1/GSTP1 inhibitors	5-Methyl-N-(5-nitro-thiazol-2-yl)-3-phenylisoxazole-4-carboxamide (MNPC)	NQO1 and GSTP1 active sites binding	Increase	Anti-tumor	Glioblastoma	[457]
Phenothiazinium redox cyclers	3,7-diaminophenothiazinium-based redox cyclers	Electron transfer and NQO1 substrate	Increase	Anti-tumor	Melanoma	[458]
HIF-1α inhibitors	2-methoxyestradiol	Mitochondrial membrane potential disruption	Increase	Anti-tumor	Neuroblastoma	[459]

*ATF-6* activating transcription factor 6, *ERT* reverse electron transfer, *FMN* flavin mononucleotide, *NADPH* nicotinamide adenine dinucleotide phosphate, *DOX* doxorubicin, *NOX* nicotinamide adenine dinucleotide phosphate oxidase, *PTX* paclitaxel, *MOFs* metal–organic frameworks, *PDT* photodynamic therapy, *RDT* radiodynamic therapy, *SDT* sonodynamic therapy, *CDT* chemodynamic therapy, *VDAC* voltage-dependent anion channel, *IKE* imidazole ketone erastin, *BSO* buthionine sulfoximine, *GCL* glutamate cysteine ligase, *PEITC* phenethyl isothiocyanate, *BITC* benzyl isothiocyanate, *NSCLC* non-small cell lung cancer, *TrxR* thioredoxin reductase, *MGd* Motexafin Gadolinium, *Prx* peroxiredoxin, *Cys* cystatin, *NQO1* NAD(P)H dehydrogenase quinone 1, *GSTP1* glutathione S-transferase P1

### Burdening cancer cells with elevated ROS load

The mitochondrial respiratory chain generates ROS, and targeting mitochondrial complexes or DNA can lead to electron leakage for excessive ROS generation. Selective inhibitors targeting the Q site of complex I can achieve a rapid superoxide generation via forward electron transport, enhancing NADH-based ROS accumulation [35, 460]. Conversely, inhibiting the upstream FMN site in Complex I reduced ROS generation [407]. In complex III, partial reduction of Q or inhibition of downstream complexes amplifies ROS production [39]. Compound SMIP003 competitively inhibits the Q site of mitochondrial complex I, resulting in high O_2_•^−^ levels that trigger the unfolded protein response and lead to 4T1 tumor cell death [406]. The commonly used biguanide-type diabetes drug, phenformin, also inhibits mitochondrial complex I Q site to induce ROS generation [408]. Metformin was found to selectively inhibit the mitochondrial complex I FMN, increasing ROS generation and playing a role in cancer prevention [461, 462]. Furthermore, melatonin can induce RET in complex I. The induced RET increased ROS generation via modification of CoQ redox and mitochondrial membrane potential in head and neck cancer cells to facilitate their apoptosis [405].

Many cancer chemotherapies exert cytotoxic effects by inducing ROS generation. Cisplatin has been widely used in treating human cancers via various mechanisms. Cisplatin induces apoptosis, probably by activating p53’s pro-oxidative function, triggering ROS generation [463]. Additionally, cisplatin can directly damage mtDNA [411, 412] and increase the mitochondrial content in ovarian cancer cells [412], ultimately promoting ROS accumulation. Arsenic trioxide inhibits mitochondrial complex IV, impairing mitochondrial respiratory function and reducing the efficiency of the electron transport chain. This leads to increased electron leakage and superoxide generation. Concurrently, it slightly decreases cellular GSH content, weakening the cell’s antioxidant capacity. The reduced antioxidant capacity allows superoxide levels to rise further, exacerbating cellular oxidative stress and ultimately causing cell damage and apoptosis [414]. Carboxyamidotriazole targets mitochondrial complex I, thus producing ROS to suppress glucose and lipid metabolism utilization to inhibit cancer development [410]. The anthracycline drug DOX (doxorubicin) also contains quinone and hydroquinone residues, which receive electrons from NADPH and generate H_2_O_2_ via a non-enzymatic reaction [464]. It may also induce excessive activation of poly(ADP-ribose) polymerase and depletion of NAD + and NADP + [418], indirectly activating NOXs and H_2_O_2_ production [417], thereby inducing tumor cell apoptosis. However, DOX-derived ROS also causes cardiotoxic side effects [465–467], limiting its application in widespread cancer therapy. Nanomedicine, a novel anti-cancer strategy, has been developed rapidly and contributes to inducing ROS-mediated cytotoxicity. PTX@TPGS-PBTE NPs can kill tumor cells via targeted delivery of PTX (paclitaxel) and induce an intracellular ROS burden [419]. Besides, the chemotherapeutic drugs cisplatin and camptothecin can be released inside tumor cells under high ROS levels, activating the cGAS (cyclic GMP–AMP synthase) -STING pathway and immune response [420], the selective drug release enhances the targeting specificity of chemotherapy drugs, which improves their efficacy while mitigating their side effects.

RT (radiation therapy) generates free radicals that damage cellular DNA. When tumor tissue is exposed to X-ray radiation, the absorption of high-energy radiation by H_2_O leads to excitation and ionization [468]. This process produces numerous free electrons and initiates cascade reactions with the surrounding H_2_O molecules, resulting in substantial ROS generation [422, 469]. However, RT dosage is limited due to the impact of ionizing radiation on healthy tissues. Radiosensitizers can accelerate ROS production and DNA damage to selectively strengthen the therapeutic effect on cancerous tissues [470].

Given the low tumor specificity of conventional cancer therapies, locally activated dynamic therapies, including PDT (photodynamic therapy), RDT (radiodynamic therapy), SDT (sonodynamic therapy), and CDT (chemodynamic therapy), have been widely employed for tumor control [427, 471, 472]. PDT delivers a designed photosensitizer to tumor sites, absorbing light of a specific wavelength and entering an excited state. Excited photosensitizers can undergo Type I and Type II reactions. Type I generates ROS by interactions with water and oxygen, while Type II produces cytotoxic singlet oxygen by activating oxygen [423]. Both reactions cause oxidative stress damage, enabling selective tumor-killing effects [423]. For instance, a bimetallic ion-modified metal–organic framework nanozyme (Zr^4+^-MOF-Ru^3+^/Pt^4+^-Ce6@HA, ZMRPC@HA) possesses catalase and glutathione oxidase activities, enhancing the oxidative stress pressure in deep-tissue tumors and successfully inhibiting their growth and differentiation [473]. Recent advances have focused on engineering photosensitizers that can be activated more effectively by different penetrating light sources [474]. While preclinical studies have extensively demonstrated the anti-tumor immunity elicited by PDT in various mouse tumor models, there is limited clinical evidence supporting this property. RDT is an emerging paradigm extending conventional PDT, which substitutes visible/infrared light with high-energy X-rays to enable photosensitizer activation in deep-seated malignancies. This overcomes the limitations of dispersed energy and the limited treatment depth of PDT [475]. For instance, the RDT system based on gold nanoclusters can directly absorb low-dose X-rays to generate ROS, efficiently killing cancer cells even under hypoxic conditions and enhancing the antitumor immune response [476]. Additionally, RDT using CsI(Na)@MgO nanoparticles and 5-aminolevulinic acid, which activates photosensitizers via X-ray irradiation, has shown significant potential for the effective treatment of deep tumors [477]. These modified nanomaterial-based RDT not only promote ROS generation but also improve targeting and deep-tissue treatment capabilities. Compared to traditional PDT, they can utilize the high penetration of X-rays more effectively to activate photosensitizers, generating a higher level of ROS in deep tumors for better therapeutic effects, thus offering new insights and methods for the clinical translation of RDT [476–479].

The SDT utilizes sonosensitizers to generate ROS under acoustic wave stimulation and exhibits superior tissue penetration with high-frequency mechanical vibration. Sonosensitizers demonstrate tumor-selective accumulation analogous to photosensitizers, enabling ultrasound-triggered ROS production for localized tumor ablation [53, 425]. Recently, alginate-coated AuNRs^ALG^ (alginate-coated gold nanorods) generated a high level of singlet oxygen after exposure to ultrasound irradiation, which induces apoptosis via the mitochondrial pathway in breast cancer cells [480]. An antibacterial nanoplatform Au@BSA-CuPpIX was designed for colorectal cancer therapy. This nanosonosensitizer was found to effectively eliminate *F. nucleatum* in colorectal cancer via ROS generation under ultrasound, thereby enhancing the therapeutic efficacy of SDT against orthotopic colorectal cancer and suppressing pulmonary metastasis. Furthermore, the incorporated gold nanoparticles reduced the phototoxicity of metal porphyrins accumulated in the skin, preventing severe inflammation and cutaneous damage [481]. Various sonosensitizers are being developed [482], and inorganic sonosensitizers are considered more advantageous regarding stability, compatibility, and photothermal conversion efficiency [483]. For instance, copper-cysteamine (Cu-Cy) nanoparticles, which serve as an inorganic sonosensitizer, have been employed in the treatment of breast cancer cells. Upon activation by FUS (focused ultrasound), these nanoparticles produce ROS that facilitate apoptosis and necrosis in tumor cells. Experimental results demonstrated that the combination of Cu-Cy and FUS treatment reduced cell viability to 45% and decreased tumor volume by 74% in 4T1 breast tumor models [484], while the Cu-Cy group showed no significant cytotoxicity when ultrasound was absent.

CDT introduces iron-based nano-drugs into the body, releasing ferrous ions under acidic conditions of the tumor and initiating Fenton reactions to convert high concentrations of H_2_O_2_ into •OH [427]. This process overcomes the limitations of the tissue penetration capacity of PDT, SDT, and RT because it does not require external energy input [485]. The TME features, including acidity and excess H_2_O_2_ [9], enable CDT’s targeted release. Excessive •OH is generated from H_2_O_2_ via Fenton reaction and can induce tumor cell death [427]. Apart from iron, various metals can act as CDT reagents in Fenton-like reactions. However, further research is necessary to develop novel metal-based/non-metal CDT formulations to improve efficacy and reduce toxicity [486]. For instance, in one study, researchers developed a new nanoplatform that can specifically release ROS in the TME through nanoparticle-triggered intratumoral catalytic chemical reactions, achieving selective killing of tumor cells [427]. This nanoplatform enhances treatment precision and reduces side effects on normal tissues. Additionally, some studies have explored the use of different metal ions in CDT and found that certain metal ions can more efficiently catalyse the Fenton reaction under specific conditions, producing more ROS and thus enhancing the killing effect on tumor cells [485, 486]. These studies offer new insights and approaches for the development of CDT, potentially further improving its applicability in tumor therapy.

### Blocking the cellular antioxidative system to inhibit cancer development

Tumor cells’ antioxidative network greatly contributes to their survival and growth, in which the GSH and Trx systems have been widely reported as critical components for tumor redox homeostasis [487]. The GSH and Trx systems consist of GSH, GSR, glutaredoxin, Trx, TrxR, and NADPH, respectively, forming the two primary independent antioxidant systems in eukaryotes [488]. Within these two systems, GSR and TrxR transfer electrons from NADPH to GSSG or Trx-S2 (oxidized Trx), converting them to their reduced states and participating in the subsequent antioxidant reactions. Meanwhile, transcription factor NRF2 governs the expression of multiple antioxidant enzymes. Various strategies have been designed to target the GSH and Trx system as well as NRF2 activities to destroy the tumor cell redox balance, thereby inhibiting tumor progression.

GSH, a critical antioxidant in cancer, is a promising target for cancer control, triggering strategies to inhibit GSH synthesis and deplete GSH reserves. GSH substrates elimination, transmembrane transport blockage, and synthesis inhibition have been investigated for cancer control. Cys is essential for GSH synthesis [489]. The system x_c_- (cystine/glutamate antiporter) is a specific cystine transporter that imports cystine from the extracellular amino acid pool [490] and is upregulated in many cancers [491]. Targeting the system x_c_- to inhibit Cys intake and GSH depletion can lead to tumor cell ferroptosis [492]. Erastin inhibits cystine uptake by blocking the system x_c_- [428, 493]. However, their poor solubility and renal toxicity limit their application. Exosome-loaded erastin enhanced therapeutic effects in cancers compared to free erastin [494]. Additionally, more water-soluble erastin analogs, including piperazine-erastin and imidazole ketone erastin, have been developed and present improved anti-cancer effects [431, 495]. The combined treatment with celastrol and erastin significantly increased ROS generation, disrupted mitochondrial membrane potential, and promoted mitochondrial fission [496]. But this finding warrants further clinical evaluation. Sorafenib, an approved anti-cancer drug for hepatocellular and renal cell carcinoma [432], also inhibits the system x_c_- [428]. Additionally, depleting blood cysteine using cyst(e)inase simultaneously facilitates system x_c_- inhibition and GSH depletion [433].

GSH depletion and synthesis inhibition also lead to tumor cell oxidative damage. GCL is the rate-limiting enzyme for GSH synthesis, and its irreversible inhibitor, BSO (buthionine sulfoximine), is commonly used as an adjuvant agent, which is demonstrated to suppress glutathione levels and induce lipid peroxidation, thereby inhibiting cell viability [434]. A nano-reaction platform based on iron-based metal–organic frameworks (MOFs), BSO&OXA@MOF-LR, loaded with OXA (oxaliplatin) and BSO, effectively inhibits GSH generation and mitigates cancer cell resistance to chemotherapy drugs [497]. The BSO-based compound, Nap-DFDFY-CS-DEVD-BSO, contains a BSO segment that inhibits intracellular GSH synthesis. This compound is reduced by GSH and transformed into Nap-DFDFY-thiol. It exhibits the dual functions of GSH depletion and inhibition, displaying high cytotoxicity [498]. Other drugs have focused on depleting existing GSH levels over a short period. Isothiocyanates, including phenethyl isothiocyanate, benzyl isothiocyanate, and sulforaphane, contain a carbon atom in the -N = C = S group that can react with the cysteine thiol group on GSH catalyzed by GST to form conjugates [499]. They effectively deplete GSH in various cancers [438, 500, 501]. Moreover, compounds containing α, β-unsaturated carbonyls, such as cinnamaldehyde, can attack GSH via Michael addition and have anti-cancer potential [502, 503]. Certain redox-active MOFs exhibit catalase-like activity that persistently catalyzes H_2_O_2_ to generate O_2._ In addition to supplementing O_2_, these MOFs can also effectively reduce GSH concentration by absorbing and oxidizing GSH [421, 504], further amplifying ROS damage.

Emerging evidence highlights the widespread overexpression of NRF2 across multiple tumor types, with its hyperactivation serving as a compensatory mechanism under oxidative stress [505], exhibiting complex roles in tumorigenesis and progression [373, 506]. Therefore, NRF2 inhibitors have garnered significant attention for cancer control. Certain natural dietary NRF2 inhibitors directly bind to NRF2 to block its activity, while others modulate upstream or downstream pathways, demonstrating antitumor potential. For example, luteolin suppresses NRF2 expression, wogonin inhibits NRF2 mRNA transcription, brusatol and halofuginone block NRF2 translation by suppressing global protein synthesis, and trigonelline prevents NRF2 nuclear translocation [507, 508]. Brusatol suppresses NRF2 protein expression, elevates ROS levels in pancreatic cancer cells to overcome chemoresistance [439], indicating therapeutic promise in cancer treatment [509]. Similar to the mechanism of brusatol, halofuginone is a febrifugine derivative, and it reduces NRF2 synthesis by inducing cellular amino acid starvation response and inhibiting global protein translation, thereby attenuating tumor cell chemoresistance [440, 510]. Certain polyphenols, such as procyanidins, can also act as NRF2 inhibitors, offering preventive and therapeutic benefits against cancer [511]. Currently, a novel and validated NRF2-targeting inhibitor ML385 has exhibited great potential in cancer inhibition. ML385’s anti-cancer effects have been widely observed in preclinical studies. ML385 directly binds to the Neh1 domain of the NRF2 protein and decreases its DNA binding capability [512]. ML385 exerted highly selective cytotoxicity to cancer cells with KEAP1 mutations and showed significant anti-cancer efficacy when combined with carboplatin treatment in NSCLC. It also inhibits the NRF2/HO-1 pathway and suppresses head and neck squamous cell carcinoma cancer cell growth and breast cancer cell stemness [441, 442]. Meanwhile, ML385 could inhibit NRF2 nuclear translocation in cancer cells treated with ionising radiation, and this increased ROS level and ferroptosis, thus sensitizing esophageal squamous cell carcinoma to radiotherapy [513]. In leukemia, ML385 treatment enhanced the efficacy of doxorubicin. Interestingly, ML385 treatment decreased KEAP1 protein levels. However, whether it directly regulates KEAP1 stability or functions via other pathways remains unclear [514]. ML385 has exhibited great potential in multiple cancer control, while no clinical trial has been launched so far.

The TrxR/Trx system is critical for eliminating ROS, such as H_2_O_2_, and has been implicated in cancer. PX-12 is the first inhibitor of Trx with clinical development, and it causes irreversible thioalkylation of Cys73 of Trx1 [515, 516]. It has exhibited inhibitory effects alone or synergistic effects with other anti-tumor drugs on distinct cancer cells [443, 517–519]. However, the evidence from clinical data has demonstrated the limited efficacy of PX-12. A previous phase I trial was launched to evaluate the effects of PX-12 on patients with advanced solid tumors. In this study, the patients were administered with PX-12 infusion at 9–300 mg/m^2^ in 1 h or 3 h, and no objective responses were observed. Only seven patients achieved stable disease, and one patient with appendiceal adenocarcinoma had a minor response of 18.3% [520]. Another phase II study of PX-12 on previously treated advanced pancreatic cancer also reported no consistent decrease in SUV, Trx-1 levels, or CA 199 levels and was terminated early due to limited antitumor activity as well as low baseline Trx-1 levels [521] (NCT00177242). Further, a prolonged infusion trial was initiated and reported no observed response either after PX-12 72-h infusion (300 mg/m^2^/24 h) by efficacy evaluation, with only one patient with rectal cancer having stable disease [522]. Similarly, a phase IB trial has demonstrated that the infusion of PX-12 with the maximally tolerated dose (300 mg/m^2^/24 h) on patients with malignant gastrointestinal cancers did not show significant clinical activity and trends in plasma Trx-1, VEGF, or FGF-2 changes [515]. In addition to PX-12, more Trx/TrxR targeting agents are being investigated recently.

Compared to Trx inhibition, TrxR-targeting strategies have gained more attention in recent years. The TrxR1 inhibitor, Motexafin Gadolinium (MGd), is a pentadentate aromatic metalloporphyrin with non-competitive TrxR1-inhibitory capability [523] to promote ROS accumulation under radiation therapy [524]. Therefore, MGd has been used as a radiosensitizer in cancers [524]. Early clinical trials have demonstrated good tolerance, selective biolocalization in tumors, and detectability at MRI imaging [525, 526] in patients with cancers. Renschler et al. conducted a series of clinical trials to investigate the performance of MGd in patients with brain metastases. In their studies, MGd administration (5 mg/kg/d) obtained a high radiologic response rate as well as high rates of freedom from neurologic progression at 1 year when combined with 30 Gy in 10 fractions of whole-brain radiation therapy in patients with brain metastases [527, 528]. This also improved time to neurologic and neurocognitive progression [529, 530]. Moreover, MGd did not cross the intact blood–brain barrier in normal brain tissue while maintaining measurable uptake and improving the median survival time (16.1 months compared to 11.8 months in the control group) in patients with GBM [531, 532]. However, opposite results were also observed that the combination of standard radiotherapy with temozolomide and MGd achieved no significant survival improvement in GBM patients [533]. In pediatric patients with newly diagnosed intrinsic pontine gliomas, the addition of MGd did not improve the survival either, compared to a standard 6-week course of radiation [534]. In non-CNS tumors, some preclinical studies have indicated the anti-tumor efficacy of MGd in blood tumors [448, 449], while the clinical results of solid tumors showed that the therapeutic effects of MGd are limited, currently. MGd infusion alone did not result in significant clinical responses in metastatic renal cell carcinoma [447]. Meanwhile, no significant therapeutic response differences were obtained for patients with different non-brain malignancies administered with a combination of MGd and doxorubicin [535]. No significant improvement in response rate, PFS, or OS was observed for NSCLC patients administered pemetrexed 500 mg/m plus MGd 15 mg/kg every 21 days, either [536].

Auranofin is a class of gold-containing compound that targets TrxR through substitution by cysteine or selenocysteine amino acid residues in the active site of TrxR [537]. It is first proved for rheumatoid arthritis but recently demonstrated potent antitumor activity both in vitro and in vivo [538, 539]. Multiple investigations focusing on auranofin alone or in combination with other anticancer agents support its repurposing as an adjuvant to overcome chemotherapy resistance or enhance conventional treatment efficacy [524, 540, 541]. Combination of Auranofin with everolimus (mTOR inhibitor) triggers synergistic cell death induction in an oxidative-stress-dependent manner, which activates autophagy, ERS, and JNKs signaling pathways in breast and colon cancer cells [542]. A recent study also involved a nanoplatform for auranofin and doxorubicin co-delivery, and this strategy significantly sensitized ferroptotic and apoptotic effects on breast cancer cells [543]. In GBM, auranofin also exhibited promising anti-cancer capabilities when combined with GSH system inhibitors L-BSO or PPL [544]. Meanwhile, auranofin alone also exerts ROS-induced apoptosis and growth inhibition in anaplastic thyroid cancer [450]. Notably, increased ROS mediated by auranofin induces PD-L1 expression, resulting in immunosuppression, while its combination with anti-PD-1 antibody enhances anticancer activity in murine B-cell lymphoma [545]. Currently, clinical trials covering phase I, II studies are being carried out to investigate auranofin’s roles in chronic lymphocytic leukemia (NCT01419491), ovarian cancer (NCT03456700), recurrent epithelial ovarian, primary peritoneal, and fallopian tube cancer (NCT01747798), lung cancer (NCT01737502), and recurrent GBM (NCT02770378), while their results have not been posted.

Ethaselen is an organoselenium-containing TrxR1 inhibitor designed by Zeng et al. [546]. Its dose-dependent inhibitory effects on NSCLC and gastric cancer cell growth have been identified [444, 445], and it also exhibits synergistic efficacy with sunitinib and sodium selenite for colon cancer and NSCLC cancer cells, respectively [547, 548]. One clinical trial investigating etheselen’s roles in disease control rate, survival improvement, and safety on NSCLC patients has been completed, but no data have been reported (NCT02166242). Butaselen is another organoselenium-containing compound for TrxR-targeting designed by Zeng et al. [549]. This novel TrxR inhibitor has presented therapeutic efficacy in hepatocellular carcinoma cells [549–551]. Butaselen administration decreased the incidence of DEN/CCL4/ethanol-induced hepatocellular carcinoma in mice and protected them from developing fibrosis and cirrhosis. Meanwhile, it inhibited hepatocarcinoma growth in the syngeneic mouse model and this is facilitated via cell cycle arrest and apoptosis induced by TrxR-inhibition [549]. Interestingly, butaselen also decreased the expression of total and phosphorylated STAT3 and downregulated PD-L1 expression in hepatocellular carcinoma cells [550]. Moreover, butaselen promoted NK and T-cell infiltration and activities in TME in vivo via upregulated CXCR3, NKG2D, and their ligands. Butaselen administration exerts synergistic effects when combined with PD-1 blockade [551]. These preclinical studies indicate butaselen’s potential for hepatocellular carcinoma control and immunotherapeutic efficacy improvement.

In addition to NRF2, GSH, and Trx/TrxR systems, other antioxidant components are present in cells, such as Prx, NQO, and SODs. The Prx system is also critical in maintaining tumor cell redox homeostasis as previously discussed, and Prx inhibition could enhance intracellular ROS accumulation, thus suitable for cancer cell elimination. Celastrol is a bioactive constituent extracted from a Chinese herb, binding to the Cys172 residue of Prx-2 to inhibit its antioxidant activity for H_2_O_2_ reduction. Celastrol can effectively inhibit the growth of gastric cancer in vivo and in vitro, suggesting its potential therapeutic effects [456]. Celastrol has been frequently developed as a candidate for nanoplatform-based co-delivery, nanohybrids, and PTORAC-based strategies in treating cancers including NSCLC, pancreatic cancer, and hepatocellular carcinoma, etc. [552–555]. Besides, celastrol exhibits immunotherapeutic potential due to its capability to induce ICD or PD-L1 expression in tumor cells [556, 557]. PHB binds to Prx-3 in the mitochondria, reducing mitochondrial ROS. Knocking out *PHB* or pharmacologically inhibiting PHB via rocaglamide A can effectively inhibit GBM growth and enhance radiotherapy activity [187]. NQO1 is elevated in gliomas, and it prevents oxidative-stress-mediated glioma cell death [457]. 5-methyl-N-(5-nitro-thiazol-2-yl)-3-phenylisoxazole-4-carboxamide has been discovered to target the active sites of NQO1 and glutathione S-transferase Pi 1 via High-throughput screening, and it exhibits significant potential as an anti-cancer drug [457]. The PRCs (3,7-diaminophenothiazinium-based redox cyclers), such as thionine, toluidine blue, and methylene blue, can function as substrates for NQO1. NQO1-mediated electron transfer from NADPH reduces these compounds to their leuco-forms, which can spontaneously transfer electrons to oxygen, generating ROS [458]. Previous studies have indicated that 2-methoxyestradiol can inhibit SOD activity, accumulating O_2_•^−^ in the mitochondria, causing mitochondrial membrane damage and cell death [558]. However, the SOD-based mechanism of 2-methoxyestradiol is suspected, and its capability to induce ROS generation [559] and inhibit cancer development has been confirmed [459].

## The involvement of ROS in cancer immunotherapy

**Fig. 6:**
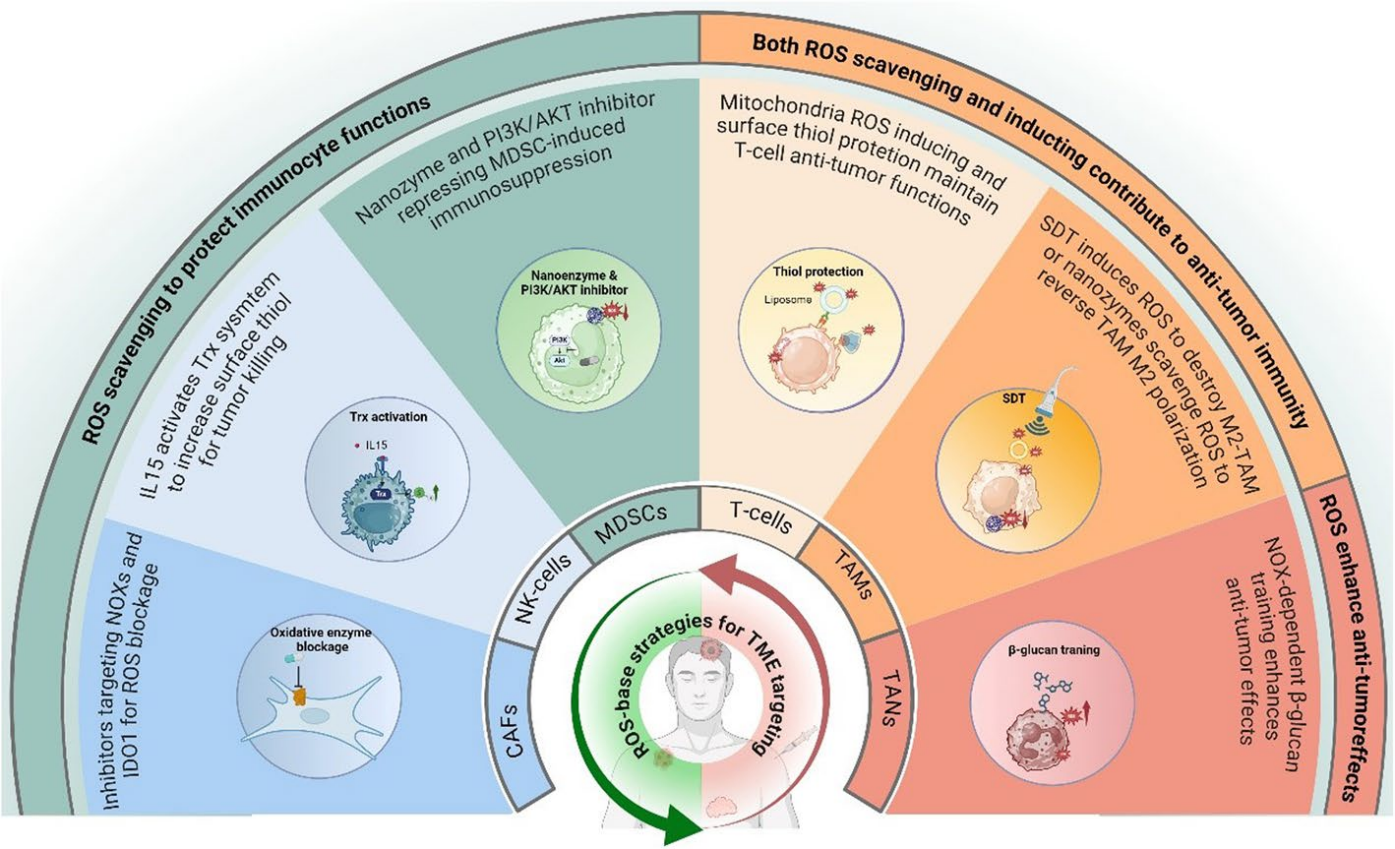
Comprehensive summary of the role of oxidative stress in TME targeting. Antioxidative strategies, including T-cell thiol protection, NK cell Trx system activation, CAF oxidative enzyme inhibition, MDSC PI3K/Akt blockage, and macrophage ROS scavenging, have been adopted to facilitate immunological cytotoxicity and immunosuppression reversal. Meanwhile, ROS-inducing β-glucan training of neutrophils and SDT treatment of M2 macrophages inhibited cancer progression. TME, tumor microenvironment. NK cells, natural killer cells. Trx, thioredoxin. CAFs, cancer-associated fibroblasts. MDSCs, myeloid-derived suppressor cells. ROS, reactive oxygen species. SDT, Sonodynamic Therapy. The figure was created using BioRender.com

Cancer immunotherapy has seen notable success in treating cancers that are resistant to standard therapies; it enhances the immune system’s anti-cancer response by boosting host immunocytes and reversing the immunosuppressive microenvironment to promote cancer elimination (Fig. 6). Improving the antioxidant capacity or ROS-based immunocyte cytotoxicity against tumor cells may enhance the effectiveness of cancer immunotherapy.

### ROS-associated strategies for anti-tumor immunity enhancement

T-Fulips is a liposome composed of anti-CD3 F(ab')2 fragments and TEMP (2,2,6,6-tetramethylpiperidine), targeting T-cells by anti-CD3 F(ab')2 fragments and protecting the –SH on the T-cell surface with TEMP, thereby preserving T-cell anti-tumor function [224]. Moreover, TEMP is converted to the paramagnetic compound TEMPO during redox reactions, allowing MRI (magnetic resonance imaging) assessment [224]. Studies have also indicated that pre-treatment with antioxidants such as N-acetyl cysteine or rapamycin can upregulate thiol levels and antioxidant gene expression in CD8 + T-cells, enhancing their antioxidant and anti-cancer capacity [560]. Venetoclax disrupts the respiratory chain supercomplex and accelerates ROS generation in CD3 + CD4-CD8- double-negative T-cells and CD8 + T-cells, enhancing CD8 + T-cell cytotoxicity [561]. In HCC, high PGAM1 (phosphoglycerate mutase 1) expression reduces the infiltration and activation of CD8 + T-cells [562]. Inhibition of PGAM1 promotes CD8 + T-cells infiltration and promotes ferroptosis in HCC cells by inducing energy stress and ROS-dependent AKT inhibition to downregulate LCN2 (lipocalin-2) [562]. In the TME, IL-15 can activate the Trx system through mTOR, increasing thiol levels on the NK cell surface to stabilize its tumor-killing capability and reverse microenvironment immunosuppression [563]. Inversely, some immune training can enhance the anti-tumor activity of innate immune cells. For instance, training TANs with β-glucan generates excessive ROS in a NOX-dependent manner to exert anti-tumor effects [564].

### ROS-associated strategies for immunosuppression elimination

The tumor immune-suppressive microenvironment is vitally important in immunotherapy blockade. Targeting and depressing immunosuppressive cells by ROS is another effective way to reshape the immunosuppressive TME. CAFs rely on continuous NOX4-mediated ROS production to maintain their immunosuppressive phenotypes. Therefore, the selective NOX1/4 inhibitor GKT137831 can specifically target CAFs, attenuating TGF-β1-induced CAF activation and reshaping the tumor immune-suppressive microenvironment [565, 566]. Inhibiting indoleamine-2,3-dioxygenase 1 and NOX2 also impedes ROS generation, disrupting the interaction between CAFs and monocytes and reducing MDSC generation [207]. The Zr-CeO nanozyme converts O_2_•^−^ to H_2_O_2_ and H_2_O_2_ to O_2_ to eliminate ROS via SOD-like and CAT-like activities, respectively [25]. Reduction of ROS levels inhibits MDSC proliferation and immunosuppression [25]. The PI3K/AKT pathway inhibitor, IPI-549, can also effectively reduce ROS levels in MDSCs, promoting MDSC apoptosis and reducing its immunosuppressive activity against CD8 + T-cells [567]. Additionally, ROS nanozyme could scavenge ROS to modulate macrophages by blocking ERK and JAK/STAT pathway activation, inhibiting their M2-related gene expression, such as *Arg1*, *Chil3*, and *Retnla*, and stimulating their M1 marker expression [24, 25, 568]. The SDT-based M2-targeting nanoparticles M-H@lip-ZA could target M2 macrophages in TME and induce their depletion. This also decreases tumor hypoxia and immunosuppressive cytokines releases, while strengthening vasculature normalization, intertumoral perfusion, and anti-cancer cytokines secretion [569].

A promising strategy to convert TME from ‘cold’ to ‘hot’ and overcome immunosuppression is to trigger ICD (immunogenic cell death) in cancer cells with long-term anti-cancer immunity [570]. ICD immunogenicity is primarily mediated by DAMPs (damage-associated molecular patterns) exposed to dying cells, including calreticulin, HSPs (heat shock proteins), secreted ATP, and high mobility group protein B1 [571]. Most of these DAMPs can be recognized by PRRs (pattern recognition receptors) on the DC surface, improving antigen presentation and CD8 + T-cell cytotoxicity [572–574]. Research has demonstrated that ERS is a prerequisite to induce ICD, and ROS critically influences ERS [571]. ICG/AuNR@BCNP was designed as an ERS nano-orchestrator targeting PERK (protein kinase R-like endoplasmic reticulum kinase)/CHOP pathway to optimize ERS control, thereby promoting ICD [575]. Elevated lactate levels due to severe hypoxia in TME and metabolic reprogramming of tumor cells (the Warburg effect) may limit ROS production [576]. PLNP^Cu^ can degrade lactate into H_2_O_2_, which is then converted into anti-tumor ROS via a Fenton-like reaction [577]. PP_IR780-ZMS_ can control the release of ZMS (manganese zinc sulfide nanoparticles) under near-infrared light, triggering Mn^2+^-mediated CDT to induce ERS [578]. Menger et al. found that cardiac glycosides (CGs) are also an ICD inducer. CGs activate ERS by inhibiting sodium–potassium ATPase on the cell membrane, increasing intracellular calcium ion concentration [579]. Micheliolide, a TrxR inhibitor, can induce ROS generation and ERS in hepatocellular carcinoma cells [580]. These effects induce robust ROS-based ERS and lead to tumor cell ICD, remodeling TME immunity. Therefore, combining immunotherapy with ROS intervention has been widely recognized as a promising strategy to overcome tumor therapeutic resistance (Table 3).

**Table 3:** The role of oxidative stress-based strategies in immunotherapies

Drugs	Mechanism	ROS	Effects	Tumors	References
T-Fulips	Improve the antioxidant capacity of T cells	NA	Anti-tumor	Breast cancer, colon cancer, and melanoma	[224]
				Melanoma	[560]
Venetoclax	Damage the respiratory chain	Increase	Anti-tumor	Acute myeloid leukemia	[561]
IL-15	Activate the Trx system of NK cells	NA	Anti-tumor	Chronic myelogenous leukemia and non-small cell lung cancer	[563]
Zr-CeO Nanozyme	SOD-like and CAT-like enzyme activation	Decrease	Anti-tumor	Renal cancer and breast cancer	[25]
IPI-549	Inhibit PI3K/AKT pathway	Decrease	Anti-tumor	Colon cancer	[567]
β-Glucan	Train TAN to generate ROS	Increase	Anti-tumor	Lung cancer and melanoma	[564]
M-H@Lip-ZA	Kill M2-TAM utilizing ROS	Increase	Anti-tumor	Breast cancer	[569]
ICG/AuNR@BCNP	Induce ICD by tumor cell ERS	Increase	Anti-tumor	Glioblastoma and melanoma	[575]
NP-I-CA-TPP	Induce ICD by consuming GSH	Increase	Anti-tumor	Osteosarcoma	[581]
PPIR780-ZMS	Induce ICD by CDT	Increase	Anti-tumor	Melanoma	[578]
Cardiac Glycosides	Induce ICD by inhibiting sodium–potassium ATPase	Increase	Anti-tumor	Breast, colorectal, head and neck, and hepatocellular carcinoma	[579]
Micheliolide	Induce ICD by inhibiting TrxR	Increase	Anti-tumor	Hepatocellular carcinoma	[580]
PLNPCu	Induce ICD by Fenton-like reaction	Increase	Anti-tumor	Breast cancer	[577]

*IL-15* interleukin-15, *CAT* catalase, *PI3Kγ* phosphoinositide 3‐kinase gamma, *TAN* tumor-associated neutrophil, *M2-TAM* M2-like tumor-associated macrophage, *ICD* immunogenic cell death

## ROS-based strategies in combinatorial therapies

Given tumor heterogeneity, single therapies can quickly induce resistance and attenuate treatment effects [582, 583], while combination therapy can reduce or delay the occurrence of drug resistance through the synergistic effect of different mechanisms.

### Combining therapies with ROS-associated strategies increases tumor-killing efficacy

Given the higher resistance to oxidative damage in tumor cells, integrating ROS-based therapies with additional treatment could increase the tumor-killing efficacy. Hui, K et al. studied the synergistic effect of bortezomib, a proteasome inhibitor, and romidepsin, a histone deacetylase inhibitor, in the treatment of gastric cancer, and found that this combined therapy could induce autophagy and apoptosis in MAPK- and ROS-dependent way, exhibiting enhanced killing effect on gastric cancer cells [584]. In GBM, cells have a high demand for iron to promote tumor growth and progression, so these cells are susceptible to ferroptosis, of which SLC7A11 (solute carrier family 7 member 11) is a key antagonist [585]. SIRT3 (Sirtuin-3) inhibition leads to the accumulation of ferrous and ROS in mitochondria, and the mitochondrial autophagy pathway is upregulated after SIRT3 knockdown in GBM cells [585]. Above, this provides an idea for a combination therapy that targets SIRT3 and inhibits SLC7A11 to induce iron death in GBM.

Recently, the advent of nanomedicine-based ROS therapy has provided a platform for combinatorial therapies. A synergistic approach can be formed by combining open-source and throttling strategies to achieve high ROS concentrations with the ability to induce tumor cell damage. PGC-DOX, an intelligent nano-catalytic theranostic, uses poly (ethylene glycol)-modified GOx (glucose oxidase) loaded with DOX to catalyze intracellular glucose to produce H_2_O_2_, facilitating an in situ Fenton reaction in conjunction with CDT to generate a high •OH level. This synergistic approach enhances the anti-tumor effect with minimal side effects [586]. Liposome-based nano-drugs co-loaded with doxycycline hydrochloride and Ce6 (chlorin e6) simultaneously disrupt mitochondrial function and enhance PDT [587]. Fe^2+^@UCM-BBD loaded with DOX, Ce6, and Fenton reagent (Fe^2+^) achieved a controlled release of DOX in the acidic TME, enabling the combined treatment of PDT, CDT, and chemotherapy [588]. GSH deprivation is critical in nanomedicine-based combinatorial therapy [589]. MCPP is a nanomedicine combining PTX and the photosensitizer P18 (purpurin 18). PTX-SS-PTX is a GSH-responsive dimeric form of PTX connected by a disulfide bond, designed to release PTX and deplete GSH in the TME. P18 generates ROS upon laser irradiation, inducing cytotoxicity and ICD, while PTX-SS-PTX enhances this effect by reducing GSH levels, creating a synergistic anti-tumor response [590].

### Integrating nano-technology with ROS-based strategies promotes immunotherapy efficacy

TME-targeting nanomaterials have been applied to integrate ROS-generating therapy with immunotherapy. A nano-drug containing a photosensitizer and PI3Kγ inhibitor uses PDT to induce ICD and inhibit immune suppression induced by the PI3Kγ/Akt pathway in MDSCs [567]. CCA-M1EVs (M1-like macrophage-derived extracellular vesicles) use surface CPPO to facilitate CDT. The drug-loaded M1EVs can penetrate the blood–brain barrier, specifically target and accumulate in gliomas enriched with M2 macrophages, and induce the polarization of M2 macrophages towards the M1 phenotype, thereby achieving immunomodulation of the TME [591]. The PIH-NO system can accumulate in the mitochondria and alleviate intracellular hypoxia to enhance SDT, leading to mitochondrial dysfunction and amplify ICD effects [592]. Meanwhile, the PIH-NO-derived NO could convert M2-TAM into M1-TAM and simultaneously deplete MDSCs in TME [592].

Moreover, nanotechnology can integrate ROS-based cytotoxicity with ICB (immune checkpoint blockade) therapy. S-αPDL1/ICG@NP-mediated PDT generates ROS and induces ICD, sensitizing the tumor to immunotherapy via subsequent anti-PD-L1 antibody release [593]. The UCNPs@Cu-Cys-GOx nano-system also generates ROS to reverse immunosuppression and strengthen PD-1/PD-L1 therapeutic effects to inhibit primary tumors [594]. Additionally, the nano-drug-mediated ROS therapies exhibit good compatibility with other immune checkpoint inhibitors, including Gal-9, Tim-3, and CD47 [595, 596]. He et al. summarized that PDT-induced ROS can also be combined with tumor vaccines, immune adjuvants, and other agents [597]. Also, the PDT-induced pyroptosis has been summarized as “photo-pyroptosis” to demonstrate PDT’s synergistic immunotherapy [598].

Nanomedicine, a promising application in cancer therapy, has demonstrated its ability to integrate multiple therapeutic effects, potentially overcoming therapeutic resistance in cancers. ROS-mediated cytotoxicity and TME remodeling have been widely considered in nanomaterial design due to their strong anti-tumor effects and compatibility with other therapies, exhibiting great potential for developing future therapeutic strategies.

## The clinical prospects of artificial intelligence, biomaterials, and imaging techniques in oxidative stress

### Artificial intelligence in oxidative damage detection and antioxidative prediction

The short lifespan of ROS in the TME and different subcellular locations makes direct measurement of ROS challenging. Machine-learning-based approaches have been applied to the experimental measurement of ROS. Some supervised learning models, such as neural networks, logistic regression, and decision trees, can quantify oxidative stress damage in biological samples [599]. In addition, Weighted Gene Expression Network Analysis with GO term enrichment could be combined with clinical analysis to identify genes with diagnostic value related to local immune and oxidative stress [600]. As previously mentioned, various substances can exert antioxidant effects. A recent study combined artificial intelligence with oxidative stress to develop a new machine-learning model to identify proteins with antioxidant properties, achieving relatively high accuracy [601, 602]. Machine-learning algorithms can predict the nanoparticle-based antioxidant efficiency [603].

### Bioengineering in ROS-based cancer therapies improvement

Various engineered biomaterials exhibit tumor-specific ROS modulation capabilities, or with nanocarrier systems, enhancing pharmaceutical ROS-regulation efficiency. Meanwhile, the ROS-responsive mechanisms enable precise microenvironmental targeting. Several nanomaterials, such as PTX@TPGS-PBTE NPs, ZMRPC@HA, Zr-CeO, PGC-DOX, and Fe^2+^@UCM-BBD, have demonstrated good anti-tumor effects and exhibit great potential when combined with immunotherapy. The development of energy-converting biomaterials, including noble metals, metal semiconductors, and MOFs, has enhanced the efficacy of energy-driven cancer therapies, including RT, PDT, and SDT, with minimal side effects [604]. A novel radiosensitizer, Met-CuS@DSH, which encapsulates metformin and copper sulfide nanoparticles in an injectable DNA supramolecular hydrogel, has been developed [605]. With targeted delivery to tumor tissues, it can effectively reverse tumor hypoxia and promote the generation of ROS [605]. In addition, some nanomaterials have antioxidant properties that can neutralize ROS. Ultra-small ruthenium nanoparticles can mimic SOD activity to scavenge O_2_•^−^, and this TME-responsive ROS scavenger can be used as an adjuvant therapeutic agent to minimize side effects and improve drug efficacy [606]. To control ROS toxicity, attempts can also be made to selectively eliminate cytotoxic ROS while retaining physiological ROS balance. Modulating the surface state of herbal CDs (carbon dots) can rationally construct dynamic ROS nanomodulators. For example, phenolic OH-containing CDs derived from honeysuckle (Lonicera flos) and dandelion showed appropriate redox potentials, and they were able to scavenge cytotoxic ROS such as •OH and ONOO^−^ [607]. However, it was ineffective against essential ROS such as O_2_•^−^, H_2_O_2,_ and NO [607].

PTK-UR (poly(thioketal)-urethane) is a porous scaffold that can deliver macrophages to tumor tissues and rapidly degrade in response to local ROS in the tumor [608]. Injectable smart hydrogels with a three-dimensional network structure can serve as carriers for anti-tumor drugs or even cells, achieving localized storage and controlled release, and can exert systemic anti-tumor effects [609]. The PAA-MnO2 mineralized hydrogel, with its ability to selectively mineralize in response to the high ROS levels found in tumor tissues, not only facilitates cancer detection through a visible sol–gel transformation but also effectively scavenges ROS, which in turn enhances the accuracy and reliability of cancer monitoring, thus demonstrating great potential for cancer monitoring [610]. Numerous emerging therapeutic strategies use hydrogels for anti-inflammatory purposes [611]. Chitosan and its derivatives produce minimal side effects and have redox regulatory potential when implanted into mammals [612]. However, their degradability, biocompatibility, and toxicity issues have yet to be fully resolved.

### Oxidative stress in imaging-based tumor monitoring

The combination of redox-sensitive agents or probes and imaging techniques has been applied to detect tumor oxidative stress status. Several MRI contrast agents, such as stable nitroxide free radicals, activatable paramagnetic complexes, and hyperpolarized [1-13C] dehydroascorbic acid, can detect redox conditions in tumor cells and tissues. Stable nitroxide free radicals are cell-permeable and provide T1 contrast through 1-electron transfer reactions, with their reduction rate dependent on ROS- and ROS-scavenging systems [613]. The redox-specific probes developed for tumor oxidative stress detection contain several types, including fluorescent, self-luminous, nano-engineered, photoacoustic, and quantum dot probes. Several fluorescent probes have been developed for the real-time monitoring of tumor oxidative stress. These probes consist mainly of a fluorophore, linker, and recognition group [614]. The fluorophore is oxidizable and produces fluorescent products upon oxidation [613]. Research has also found that some self-luminous nano-probes can image high-ROS inflammation sites [615], with some possessing strong anti-tumor properties [616]. A nano-probe based on peroxalate ester derived from vitamin E emits a chemiluminescent signal to precisely locate disease sites under high H_2_O_2_ conditions in the TME [617]. A photoacoustic probe, based on a BODIPY scaffold, can cross the blood–brain barrier and perform dynamic imaging of oxidative stress in the mouse brain using PA imaging. This probe responds reversibly and ratiometrically to oxidative stress, changing its optical properties in the presence of ROS [618]. Small quantum dot-based sensors, which can be detected using electron paramagnetic resonance, MRI, and optical imaging, are capable of tracking and monitoring the overall redox state and oxidative stress in cells and tissues. Recently, quantum dots were found to have size and surface properties that can be tailored to respond to specific redox changes, making them effective for detecting the altered redox environments often found in tumors [619]. Currently, these probes confront multifaceted challenges, including controllable synthesis, toxicity, metabolic clearance, and more convenient imaging technologies [620]. However, future development in nanomanufacturing and bioinspired surface engineering is promising to enable more advanced probe synthesis and precise control over probe biodistribution. Meanwhile, the biodegradable scaffold platforms will enable in vivo clearance to reduce metabolic risk, accelerating their translation.

Combining fluorescent probes with various imaging techniques allows non-invasive, real-time, highly sensitive, and high-resolution monitoring of cellular physiological and pathological states. Meanwhile, the abnormal oxidative stress features in TME provide precise tracking of tumor lesions in vivo for better tumor monitoring. PET imaging is being used to visualize the interior of tumors, providing better insights into the TME and aiding in the development of appropriate diagnostic and treatment plans [613]. (18)F-FASu is a PET tracer that targets the system x_c_- transporter, which is upregulated in tumor cells under oxidative stress to increase cystine uptake for glutathione synthesis; it allows for high-specificity tumor imaging with lower background noise than 18F-FDG [621]. Some fluorescent probes can perform in vivo imaging of specific endogenous ROS, GSH, and other endogenous antioxidant components [614], as well as oxidative damage products (lipids, proteins, and DNA) [622], and can differentiate between tumor and normal tissues [623]. Luminescent fluorescent probes can specifically and sensitively detect gamma-glutamyl transferase (GGT), which is overexpressed in many tumors and associated with their growth, invasion, and resistance to chemotherapy, thus enabling real-time imaging of tumor biology. These probes contain a GGT-cleavable peptide substrate linked to a fluorescent dye, and upon specific recognition and hydrolysis by GGT, the dye is released and fluoresces, allowing for precise localization and monitoring of tumor development and treatment response [624].

## The limitation of ROS-based therapy in clinical practice

### The spatiotemporal dynamics of ROS limit ROS-based therapies

While ROS-based therapeutic strategies demonstrate promising potential, they are constrained by several limitations. ROS encompasses a group of molecules such as O_2_•^−^ and H_2_O_2_, exhibiting divergent biological roles. Significant differences exist in their chemical reactivity, half-lives, and subcellular localization. In both clinical and preclinical studies, the specific ROS species involved are often generalized as “ROS” without further differentiation, largely due to the rapid interconversion rates among ROS and technical challenges in their detection. Therefore, additional studies are warranted to delineate their individual properties and tumor-specific mechanisms in cancer biology. ROS may exert distinct roles in different stages of cancer development [625]. This dynamic nature necessitates precise spatiotemporal regulation of ROS during therapeutic interventions. However, current technologies face challenges in achieving real-time monitoring and dynamically adjustable treatment strategies, potentially leading to uncontrollable therapeutic outcomes.

### The off-target effects of ROS-based strategies increased their toxicity to healthy tissues

The complexity of ROS regulation remains incompletely elucidated. On the one hand, physiological levels of ROS function as critical signaling molecules and are essential for cellular physiology, including immunocytes, with their levels precisely regulated. However, elevated ROS levels also trigger oncogenic signaling pathways to promote tumor initiation and progression. On the other hand, elevated ROS levels could be utilized to induce tumor cell death, but they can concurrently damage immunocytes’ function. This narrow therapeutic window poses challenges in achieving sufficient cancer cell eradication without harming non-malignant cells. For instance, the liver, as the principal metabolic organ for metabolizing most chemotherapeutic agents, generates excessive ROS during drug detoxification, and the resultant oxidative stress has been linked to hepatic pathologies [626]. Arsenic trioxide induces oxidative stress through multiple pathways, leading to hepatotoxicity [627]. Additionally, doxorubicin triggers oxidative stress via Fenton reactions, generating O_2_•^−^ and •OH, which damage enzymes, lipids, and nucleic acids in cardiomyocytes [628]. Similar to cardiomyocytes, neurons are highly vulnerable to irreversible oxidative damage due to their non-renewable nature, significantly compromising patients’ quality of life. Certain chemotherapeutics, such as cisplatin, paclitaxel, and oxaliplatin, have been shown to induce oxidative stress in the central or peripheral nervous systems, contributing to neurotoxicity [629]. Therefore, when clinically intervening to modulate redox balance, it is critical to determine the proper concentration of ROS to be precisely regulated in the target region. One feasible strategy is to integrate antioxidant supplementation to protect healthy tissues from drug-induced oxidative damage. A recently initiated clinical trial (NCT05539053) is evaluating the preventive and therapeutic efficacy of NAC against PIPN (paclitaxel-induced peripheral neuropathy) in patients diagnosed with ovarian, tubal, and peritoneal malignancies. This trial stratifies participants into three arms: a short-course NAC regimen (2,400 mg/day orally administered for one week per paclitaxel cycle), a long-course NAC regimen (2,400 mg/day daily over nine weeks), and a control group receiving paclitaxel monotherapy. The PIPN incidence rates and severity were compared among these patients during chemotherapy administration. Similarly, the protective effects of MitoQ supplementation on doxorubicin-induced cardiovascular toxicity via oxidative stress were prospectively evaluated in breast cancer patients (NCT05146843). The oxidative stress markers, endothelium-dependent dysfunction of peripheral vascular beds, arterial stiffness, central blood pressure, and physical capability were compared in patients receiving MitoQ and placebo 20 mg per day during chemotherapy. Currently, the results of these trials have not been posted. Furthermore, while certain agents exhibit antitumor efficacy by modulating ROS levels, the underlying mechanisms remain incompletely elucidated, with non-ROS-related pathways being increasingly identified. These off-target effects may not be detrimental; instead, combination therapies integrating ROS modulation could demonstrate synergistic antitumor activity superior to ROS-targeted monotherapies.

### The low bioavailability and therapeutic resistance decreased the efficacy of ROS-based treatments

Many ROS-targeting therapies require an adequate oxygen supply to generate cytotoxic ROS. However, hypoxia is a hallmark of many solid tumors [630], which restricts ROS production and consequently diminishes therapeutic efficacy. Meanwhile, ROS inducers or scavengers frequently face bioavailability challenges due to biological barriers. Nanotechnology has great potential to improve pharmaceutical bioavailability, as nanoscale formulations have demonstrated that local or systemic delivery of these compounds to the target site improves pharmacokinetics and enhances the delivery kinetics [631, 632]. Some of the nanomedicines, such as PGX-DOX, CCA-M1EV, PTX@TPGS-PBTE NPs, and MET-CuS@DSH mentioned earlier, demonstrate the ability to target tumor cells. In addition, Fe^2+^@UCM-BBD enables controlled drug release within tumor tissues, while carbon dots enable the selective scavenging of cytotoxic ROS, thereby mitigating ROS-induced damage to normal tissues. Moreover, tumors activate adaptive antioxidant defense to counteract damage induced by oxidative stress. Whereas, it remains unclear whether the tumor cell subpopulation surviving from the attack of ROS overload intervention could exhibit enhanced antioxidant capacity. This may raise a challenge that the drug dosage required for ROS-inducing tumor cell killing might increase over time, leading to therapeutic resistance.

## Conclusion

Oxidative stress is involved in various cancer hallmarks and has been a hotspot in investigating cancerous targets. However, oxidative stress regulation is highly dynamic and complex due to various ROS sources and abundant oxidative stress modulators inside tumor cells and TME. Therefore, we introduced endogenous ROS generation and the oxidative regulatory network. The main source of ROS is the mitochondrial electron transport chain [33]. The electron released from complex I and III generates O_2_•^−^ when reacting with oxygen, the O_2_•^−^ are subsequently converted into ONOO^−^ with NO or H_2_O_2_ by SODs. The ER, peroxisomes, and NOXs in the cytoplasm also generate abundant ROS. Multiple ROS generators provide various targets for ROS-based therapies. Although the roles of oxidative stress and ROS in cancer have been widely revealed, the complexity of ROS regulation severely limited the development of interventions that employ a single supplement or single target blockage in tumor cells due to their poor tumor absorbability [363], specificity [372], and unsatisfactory anti-cancer effects [270], probably leading to unsatisfying outcomes of clinical administration of single antioxidant or ROS inhibitor. Nevertheless, targeting ROS to remodel TME has exhibited exciting discoveries. Altering ROS levels in the TME has presented enhanced cytotoxicity of CD8 + T-cells [560] and NK cells [563] and alleviated TME immunosuppression by depleting MDSCs [567] and M2 macrophages [24, 25, 568]. However, one issue should be noted that the tumor cells and non-tumor cells in the TME exhibited diverse sensitivities to certain ROS levels, and altering the tumor ROS level might cause unpredictable effects on tumor progression. Given the importance of TME in cancer development, TME-targeting ROS strategies warrant further investigation to control cancer, and TME-targeting ROS treatment should be robustly verified using credible animal model experiments.

Moreover, biomaterials and nanotechnology combined with ROS-based therapies can precisely deliver drugs to tumor tissues, overcoming the limitations of poor drug selectivity, extending the duration of drug action, and penetrating biological barriers. This can increase the local concentration of drugs in tumor tissues, thereby enhancing therapeutic efficacy while reducing cytotoxicity to healthy tissues. Some drugs can release oxygen at their site of action, significantly increasing ROS generation. Nanomedicine can maximize ROS cytotoxicity inside tumors via selective local activation and strong dynamic effects [587], induce ICD [580], or remodel the TME for extended tumor control [567]. Besides, it provides a platform for synergistic combinatorial treatment to facilitate multiple targeting, including GSH depletion [590] and lactate degradation [577]. Nano-based ROS strategies are compatible with ICB therapeutic reagents [595, 596] and can enhance anti-PD-L1 treatment [594]. The integration of ROS-based strategies and ICB therapy on nano-and biomaterial platforms holds promise for further development because ICB therapy garners significant attention for its ability to control advanced cancers. Imaging techniques allow for in vivo imaging of oxidative stress, enabling non-invasive, real-time, high-sensitivity, and high-resolution monitoring of the physiological and pathological states of cells. A novel practice related to oxidative stress is that various artificial intelligence learning models can be used to quantify ROS levels and efficiently screen for potential antioxidants.

Although numerous strategies have been developed with increasing understanding of the role of oxidative stress in cancer pathology, the regulatory network of oxidative stress in cancer remains highly interconnected and complex. More laboratory and clinical attempts are required to gradually obtain precise evaluation of distinct oxidative-stress-targeting strategies in cancer control.

## Data Availability

No datasets were generated or analysed during the current study.

## References

[1] Sies H. Oxidative stress: a concept in redox biology and medicine. Redox Biol. 2015;4:180–3. 25588755 10.1016/j.redox.2015.01.002 PMC4309861

[2] Rajaraman P, Hutchinson A, Rothman N, Black PM, Fine HA, Loeffler JS, Selker RG, Shapiro WR, Linet MS, Inskip PD. Oxidative response gene polymorphisms and risk of adult brain tumors. Neuro Oncol. 2008;10:709–15. 18682580 10.1215/15228517-2008-037 PMC2666247

[3] Lushchak VI. Adaptive response to oxidative stress: bacteria, fungi, plants and animals. Comp Biochem Physiol C Toxicol Pharmacol. 2011;153:175–90. 20959147 10.1016/j.cbpc.2010.10.004

[4] Kishi S, Nagasu H, Kidokoro K, Kashihara N. Oxidative stress and the role of redox signalling in chronic kidney disease. Nat Revs Nephrol. 2024;20(2):101-19. 10.1038/s41581-023-00775-0. 10.1038/s41581-023-00775-0 37857763

[5] CAndrés CMC, Pérez de la Lastra JM, Andrés Juan C, Plou FJ, Pérez-Lebeña E. Superoxide Anion Chemistry—Its Role at the Core of the Innate Immunity. Int J Mol Sci. 2023;24(3):1841. 10.3390/ijms24031841. 10.3390/ijms24031841 PMC9916283 36768162

[6] Hanahan D, Weinberg RA. The hallmarks of cancer. Cell. 2000;100:57–70. 10647931 10.1016/s0092-8674(00)81683-9

[7] Hanahan D, Weinberg RA. Hallmarks of cancer: the next generation. Cell. 2011;144:646–74. 21376230 10.1016/j.cell.2011.02.013

[8] Fane M, Weeraratna AT. How the ageing microenvironment influences tumour progression. Nat Rev Cancer. 2020;20:89–106. 31836838 10.1038/s41568-019-0222-9 PMC7377404

[9] Clavreul A, Guette C, Faguer R, Tétaud C, Boissard A, Lemaire L, Rousseau A, Avril T, Henry C, Coqueret O, Menei P. Glioblastoma-associated stromal cells (GASCs) from histologically norm al surgical margins have a myofibroblast phenotype and angiogenic properties. J Pathol. 2014;233:74–88. 24481573 10.1002/path.4332

[10] Jain S, Rick JW, Joshi R, Beniwal A, Spatz J, Chang ACC, Nguyen AT, Sudhir S, Chandra A, Haddad A, et al. Identification of cancer-associated fibroblasts in glioblastoma and defining their pro-tumoral effects. Cold Spring Harbor Laboratory; 2021.

[11] Biffi G, Tuveson DA. Diversity and Biology of Cancer-Associated Fibroblasts. Physiol Revs. 2021;101(1):147-76. 10.1152/physrev.00048.2019. 10.1152/physrev.00048.2019 PMC7864232 32466724

[12] Mintz J, Vedenko A, Rosete O, Shah K, Goldstein G, Hare JM, Ramasamy R, Arora H. Current advances of nitric oxide in cancer and anticancer therapeutics. Vaccines (Basel). 2021;9:94. 33513777 10.3390/vaccines9020094 PMC7912608

[13] Vitale I, Manic G, Coussens LM, Kroemer G, Galluzzi L. Macrophages and metabolism in the tumor microenvironment. Cell Metab. 2019;30:36–50. 31269428 10.1016/j.cmet.2019.06.001

[14] Ni D, Zhou H, Wang P, Xu F, Li C. Visualizing macrophage phenotypes and polarization in diseases: from biomarkers to molecular probes. Phenomics. 2023;3:613–38. 38223685 10.1007/s43657-023-00129-7 PMC10781933

[15] Gao J, Liang Y, Wang L. Shaping polarization of tumor-associated macrophages in cancer immunotherapy. Front Immunol. 2022;13:888713. 35844605 10.3389/fimmu.2022.888713 PMC9280632

[16] Dubinski D, Wölfer J, Hasselblatt M, Schneider-Hohendorf T, Bogdahn U, Stummer W, Wiendl H, Grauer OM. CD4+ T effector memory cell dysfunction is associated with the accumul ation of granulocytic myeloid-derived suppressor cells in glioblastoma patients. Neuro Oncol. 2016;18:807–18. 26578623 10.1093/neuonc/nov280 PMC4864257

[17] Mei X, Chen YS, Chen FR, Xi SY, Chen ZP. Glioblastoma stem cell differentiation into endothelial cells evidence d through live-cell imaging. Neuro Oncol. 2017;19:1109–18. 28340100 10.1093/neuonc/nox016 PMC5570159

[18] Kamiński MM, Sauer SW, Kamiński M, Opp S, Ruppert T, Grigaravičius P, Grudnik P, Gröne HJ, Krammer PH, Gülow K. T cell activation is driven by an ADP-dependent glucokinase linking enhanced glycolysis with mitochondrial reactive oxygen species generation. Cell Rep. 2012;2:1300–15. 23168256 10.1016/j.celrep.2012.10.009

[19] Simula L, Fumagalli M, Vimeux L, Rajnpreht I, Icard P, Birsen G, An D, Pendino F, Rouault A, Bercovici N, et al. Mitochondrial metabolism sustains CD8(+) T cell migration for an efficient infiltration into solid tumors. Nat Commun. 2024;15:2203. 38467616 10.1038/s41467-024-46377-7 PMC10928223

[20] Varanasi SK, Chen D, Liu Y, Johnson MA, Miller CM, Ganguly S, Lande K, LaPorta MA, Hoffmann FA, Mann TH, et al. Bile acid synthesis impedes tumor-specific T cell responses during liver cancer. Science. 2025;387:192–201. 39787217 10.1126/science.adl4100 PMC12166762

[21] Zhang Y, Choksi S, Chen K, Pobezinskaya Y, Linnoila I, Liu ZG. ROS play a critical role in the differentiation of alternatively activated macrophages and the occurrence of tumor-associated macrophages. Cell Res. 2013;23:898–914. 23752925 10.1038/cr.2013.75 PMC3698641

[22] Mo W, Liu S, Zhao X, Wei F, Li Y, Sheng X, Cao W, Ding M, Zhang W, Chen X, et al. ROS scavenging nanozyme modulates immunosuppression for sensitized cancer immunotherapy. Adv Healthc Mater. 2023;12:e2300191. 37031357 10.1002/adhm.202300191

[23] Teng Y, Xu L, Li W, Liu P, Tian L, Liu M. Targeting reactive oxygen species and fat acid oxidation for the modulation of tumor-associated macrophages: a narrative review. Front Immunol. 2023;14:1224443. 37545527 10.3389/fimmu.2023.1224443 PMC10401428

[24] Kraaij MD, Savage ND, van der Kooij SW, Koekkoek K, Wang J, van den Berg JM, Ottenhoff TH, Kuijpers TW, Holmdahl R, van Kooten C, Gelderman KA. Induction of regulatory T cells by macrophages is dependent on production of reactive oxygen species. Proc Natl Acad Sci U S A. 2010;107:17686–91. 20861446 10.1073/pnas.1012016107 PMC2955141

[25] Ma J, Yuan H, Zhang J, Sun X, Yi L, Li W, Li Z, Fu C, Zheng L, Xu X, et al. An ultrasound-activated nanoplatform remodels tumor microenvironment through diverse cell death induction for improved immunotherapy. J Control Release. 2024;370:501–15. 38703950 10.1016/j.jconrel.2024.05.001

[26] Lushchak VI, Storey KB. Oxidative stress concept updated: definitions, classifications, and regulatory pathways implicated. Excli J. 2021;20:956–67. 34267608 10.17179/excli2021-3596 PMC8278216

[27] Powers SK, Ji LL, Kavazis AN, Jackson MJ. Reactive oxygen species: impact on skeletal muscle. Compr Physiol. 2011;1:941–69. 23737208 10.1002/cphy.c100054 PMC3893116

[28] Go YM, Jones DP. Redox theory of aging: implications for health and disease. Clin Sci (Lond). 2017;131:1669–88. 28667066 10.1042/CS20160897 PMC5773128

[29] Jomova K, Raptova R, Alomar SY, Alwasel SH, Nepovimova E, Kuca K, Valko M. Reactive oxygen species, toxicity, oxidative stress, and antioxidants: chronic diseases and aging. Arch Toxicol. 2023;97:2499–574. 37597078 10.1007/s00204-023-03562-9 PMC10475008

[30] Sarniak A, Lipińska J, Tytman K, Lipińska S. Endogenous mechanisms of reactive oxygen species (ROS) generation. Postepy Hig Med Dosw (Online). 2016;70:1150–65. 27892899 10.5604/17322693.1224259

[31] Zhang N, Zhao N, Xie LS, Huang B, Lin SR, Zhang Q, Zhu YB, Wu QF, Yu SG. Mitochondrial respiratory chain and its regulatory elements SIRT1 and SIRT3 play important role in the initial process of energy conversion after moxibustion at local skin. Evid Based Complement Alternat Med. 2020;2020:2343817. 32904439 10.1155/2020/2343817 PMC7456489

[32] Pryde KR, Hirst J. Superoxide is produced by the reduced flavin in mitochondrial complex I: a single, unified mechanism that applies during both forward and reverse electron transfer. J Biol Chem. 2011;286:18056–65. 21393237 10.1074/jbc.M110.186841 PMC3093879

[33] Murphy MP. How mitochondria produce reactive oxygen species. Biochem J. 2009;417:1–13. 19061483 10.1042/BJ20081386 PMC2605959

[34] Onukwufor JO, Berry BJ, Wojtovich AP. Physiologic implications of reactive oxygen species production by mitochondrial complex I reverse electron transport. Antioxidants (Basel). 2019;8:285. 31390791 10.3390/antiox8080285 PMC6719910

[35] Robb EL, Hall AR, Prime TA, Eaton S, Szibor M, Viscomi C, James AM, Murphy MP. Control of mitochondrial superoxide production by reverse electron transport at complex I. J Biol Chem. 2018;293:9869–79. 29743240 10.1074/jbc.RA118.003647 PMC6016480

[36] Nolfi-Donegan D, Braganza A, Shiva S. Mitochondrial electron transport chain: oxidative phosphorylation, oxidant production, and methods of measurement. Redox Biol. 2020;37:101674. 32811789 10.1016/j.redox.2020.101674 PMC7767752

[37] Bleier L, Wittig I, Heide H, Steger M, Brandt U, Dröse S. Generator-specific targets of mitochondrial reactive oxygen species. Free Radic Biol Med. 2015;78:1–10. 25451644 10.1016/j.freeradbiomed.2014.10.511

[38] Sies H, Jones DP. Reactive oxygen species (ROS) as pleiotropic physiological signalling agents. Nat Rev Mol Cell Biol. 2020;21:363–83. 32231263 10.1038/s41580-020-0230-3

[39] Bedard K, Krause KH. The NOX family of ROS-generating NADPH oxidases: physiology and pathophysiology. Physiol Rev. 2007;87:245–313. 17237347 10.1152/physrev.00044.2005

[40] Fridovich I. Superoxide radical and superoxide dismutases. Annu Rev Biochem. 1995;64:97–112. 7574505 10.1146/annurev.bi.64.070195.000525

[41] Szabó C, Ischiropoulos H, Radi R. Peroxynitrite: biochemistry, pathophysiology and development of therapeutics. Nat Rev Drug Discov. 2007;6:662–80. 17667957 10.1038/nrd2222

[42] Wu W, Zhang C, Rees TW, Liao X, Yan X, Chen Y, Ji L, Chao H. Lysosome-targeting iridium(III) probe with near-infrared emission for the visualization of NO/O(2)(•-) crosstalk via in vivo peroxynitrite imaging. Anal Chem. 2020;92:6003–9. 32212607 10.1021/acs.analchem.0c00259

[43] Miao L, St Clair DK. Regulation of superoxide dismutase genes: implications in disease. Free Radic Biol Med. 2009;47:344–56. 19477268 10.1016/j.freeradbiomed.2009.05.018 PMC2731574

[44] Nisimoto Y, Diebold BA, Cosentino-Gomes D, Lambeth JD. Nox4: a hydrogen peroxide-generating oxygen sensor. Biochemistry. 2014;53:5111–20. 25062272 10.1021/bi500331y PMC4131900

[45] Pikuleva IA, Waterman MR. Cytochromes p450: roles in diseases. J Biol Chem. 2013;288:17091–8. 23632021 10.1074/jbc.R112.431916 PMC3682515

[46] Denisov IG, Makris TM, Sligar SG, Schlichting I. Structure and chemistry of cytochrome P450. Chem Rev. 2005;105:2253–77. 15941214 10.1021/cr0307143

[47] Veith A, Moorthy B. Role of cytochrome P450S in the generation and metabolism of reactive oxygen species. Curr Opin Toxicol. 2018;7:44–51. 29527583 10.1016/j.cotox.2017.10.003 PMC5841237

[48] Zito E. ERO1: a protein disulfide oxidase and H2O2 producer. Free Radic Biol Med. 2015;83:299–304. 25651816 10.1016/j.freeradbiomed.2015.01.011

[49] Fransen M, Nordgren M, Wang B, Apanasets O. Role of peroxisomes in ROS/RNS-metabolism: implications for human disease. Biochim Biophys Acta. 2012;1822:1363–73. 22178243 10.1016/j.bbadis.2011.12.001

[50] Forrester SJ, Kikuchi DS, Hernandes MS, Xu Q, Griendling KK. Reactive oxygen species in metabolic and inflammatory signaling. Circ Res. 2018;122:877–902. 29700084 10.1161/CIRCRESAHA.117.311401 PMC5926825

[51] Orrico F, Lopez AC, Saliwonczyk D, Acosta C, Rodriguez-Grecco I, Mouro-Chanteloup I, Ostuni MA, Denicola A, Thomson L, Möller MN. The permeability of human red blood cell membranes to hydrogen peroxide is independent of aquaporins. J Biol Chem. 2022;298:101503. 34929164 10.1016/j.jbc.2021.101503 PMC8753180

[52] Orrico F, Lopez AC, Silva N, Franco M, Mouro-Chanteloup I, Denicola A, Ostuni MA, Thomson L, Möller MN. Hydrogen peroxide diffusion across the red blood cell membrane occurs mainly by simple diffusion through the lipid fraction. Free Radic Biol Med. 2025;226:389–96. 39551450 10.1016/j.freeradbiomed.2024.11.031

[53] Bienert GP, Chaumont F. Aquaporin-facilitated transmembrane diffusion of hydrogen peroxide. Biochim Biophys Acta. 2014;1840:1596–604. 24060746 10.1016/j.bbagen.2013.09.017

[54] Wagner K, Unger L, Salman MM, Kitchen P, Bill RM, Yool AJ. Signaling mechanisms and pharmacological modulators governing diverse aquaporin functions in human health and disease. Int J Mol Sci. 2022;23:1388. 35163313 10.3390/ijms23031388 PMC8836214

[55] Chevriau J, De Palma GZ, Jozefkowicz C, Vitali V, Canessa Fortuna A, Ayub N, Soto G, Bienert GP, Zeida A, Alleva K. Permeation mechanisms of hydrogen peroxide and water through plasma membrane intrinsic protein aquaporins. Biochem J. 2024;481:1329–47. 39136178 10.1042/BCJ20240310

[56] Rodrigues C, Pimpão C, Mósca AF, Coxixo AS, Lopes D, da Silva IV, Pedersen PA, Antunes F, Soveral G. Human aquaporin-5 facilitates hydrogen peroxide permeation affecting adaption to oxidative stress and cancer cell migration. Cancers (Basel). 2019;11:932. 31277235 10.3390/cancers11070932 PMC6678198

[57] Guo Z, Zhang H, Liu X, Zhao Y, Chen Y, Jin J, Guo C, Zhang M, Gu F, Ma Y. Water channel protein AQP1 in cytoplasm is a critical factor in breast cancer local invasion. J Exp Clin Cancer Res. 2023;42:49. 36803413 10.1186/s13046-023-02616-1 PMC9940370

[58] Moon CS, Moon D, Kang SK. Aquaporins in cancer biology. Front Oncol. 2022;12:782829. 35847914 10.3389/fonc.2022.782829 PMC9278817

[59] Sies H. Hydrogen peroxide as a central redox signaling molecule in physiologic al oxidative stress: oxidative eustress. Redox Biol. 2017;11:613–9. 28110218 10.1016/j.redox.2016.12.035 PMC5256672

[60] Harris IS, DeNicola GM. The complex interplay between antioxidants and ROS in cancer. Trends Cell Biol. 2020;30:440–51. 32303435 10.1016/j.tcb.2020.03.002

[61] Endale HT, Tesfaye W, Mengstie TA. ROS induced lipid peroxidation and their role in ferroptosis. Front Cell Dev Biol. 2023;11:1226044. 37601095 10.3389/fcell.2023.1226044 PMC10434548

[62] Gęgotek A, Skrzydlewska E. Lipid peroxidation products’ role in autophagy regulation. Free Radic Biol Med. 2024;212:375–83. 38182071 10.1016/j.freeradbiomed.2024.01.001

[63] Dinkova-Kostova AT, Kostov RV, Canning P. Keap1, the cysteine-based mammalian intracellular sensor for electrophiles and oxidants. Arch Biochem Biophys. 2017;617:84–93. 27497696 10.1016/j.abb.2016.08.005 PMC5339396

[64] Sun J, Brand M, Zenke Y, Tashiro S, Groudine M, Igarashi K. Heme regulates the dynamic exchange of Bach1 and NF-E2-related factors in the Maf transcription factor network. Proc Natl Acad Sci U S A. 2004;101:1461–6. 14747657 10.1073/pnas.0308083100 PMC341742

[65] Zenke-Kawasaki Y, Dohi Y, Katoh Y, Ikura T, Ikura M, Asahara T, Tokunaga F, Iwai K, Igarashi K. Heme induces ubiquitination and degradation of the transcription factor Bach1. Mol Cell Biol. 2007;27:6962–71. 17682061 10.1128/MCB.02415-06 PMC2099246

[66] Marengo B, Nitti M, Furfaro AL, Colla R, Ciucis CD, Marinari UM, Pronzato MA, Traverso N, Domenicotti C. Redox Homeostasis and Cellular Antioxidant Systems: Crucial Players in Cancer Growth and Therapy. Oxid Med Cell Longev. 2016;2016(1). 10.1155/omcl.v2016.1 10.155/2016/6235641. 10.1155/2016/6235641 PMC4932173 27418953

[67] Ngo V, Duennwald ML. Nrf2 and Oxidative Stress: A General Overview of Mechanisms and Implications in Human Disease. Antioxid. 2022;11(12):2345. 10.3390/antiox11122345. 10.3390/antiox11122345 PMC9774434 36552553

[68] Adinolfi S, Patinen T, Jawahar Deen A, Pitkänen S, Härkönen J, Kansanen E, Küblbeck J, Levonen AL. The KEAP1-NRF2 pathway: targets for therapy and role in cancer. Redox Biol. 2023;63:102726. 37146513 10.1016/j.redox.2023.102726 PMC10189287

[69] Cross DA, Alessi DR, Cohen P, Andjelkovich M, Hemmings BA. Inhibition of glycogen synthase kinase-3 by insulin mediated by protein kinase B. Nature. 1995;378:785–9. 8524413 10.1038/378785a0

[70] Xu X, Zhang Y, Li W, Miao H, Zhang H, Zhou Y, Li Z, You Q, Zhao L, Guo Q. Wogonin reverses multi-drug resistance of human myelogenous leukemia K562/A02 cells via downregulation of MRP1 expression by inhibiting Nrf2/ARE signaling pathway. Biochem Pharmacol. 2014;92:220–34. 25264278 10.1016/j.bcp.2014.09.008

[71] You GR, Chang JT, Li YL, Huang CW, Tsai YL, Fan KH, Kang CJ, Huang SF, Chang PH, Cheng AJ. MYH9 facilitates cell invasion and radioresistance in head and neck cancer via modulation of cellular ROS levels by activating the MAPK-Nrf2-GCLC pathway. Cells. 2022;11:2855. 36139430 10.3390/cells11182855 PMC9497050

[72] Li C, Liu M, Deng L, Luo D, Ma R, Lu Q. Oxyberberine ameliorates TNBS-induced colitis in rats through suppressing inflammation and oxidative stress via Keap1/Nrf2/NF-κB signaling pathways. Phytomedicine. 2023;116:154899. 37247589 10.1016/j.phymed.2023.154899

[73] Jerotic D, Matic M, Suvakov S, Vucicevic K, Damjanovic T, Savic-Radojevic A, Pljesa-Ercegovac M, Coric V, Stefanovic A, Ivanisevic J, et al. Association of Nrf2, SOD2 and GPX1 polymorphisms with biomarkers of oxidative distress and survival in end-stage renal disease patients. Toxins (Basel). 2019;11:431. 31340563 10.3390/toxins11070431 PMC6669734

[74] Yang W, Wang Y, Zhang C, Huang Y, Yu J, Shi L, Zhang P, Yin Y, Li R, Tao K. Maresin1 protect against ferroptosis-induced liver injury through ROS inhibition and Nrf2/HO-1/GPX4 activation. Front Pharmacol. 2022;13:865689. 35444546 10.3389/fphar.2022.865689 PMC9013935

[75] Kang JS, Nam LB, Yoo OK, Keum YS. Molecular mechanisms and systemic targeting of NRF2 dysregulation in cancer. Biochem Pharmacol. 2020;177:114002. 32360363 10.1016/j.bcp.2020.114002

[76] Glorieux C, Sandoval JM, Fattaccioli A, Dejeans N, Garbe JC, Dieu M, Verrax J, Renard P, Huang P, Calderon PB. Chromatin remodeling regulates catalase expression during cancer cells adaptation to chronic oxidative stress. Free Radic Biol Med. 2016;99:436–50. 27591797 10.1016/j.freeradbiomed.2016.08.031

[77] Park EY, Cho IJ, Kim SG. Transactivation of the PPAR-responsive enhancer module in chemopreventive glutathione S-transferase gene by the peroxisome proliferator-activated receptor-gamma and retinoid X receptor heterodimer. Cancer Res. 2004;64:3701–13. 15150131 10.1158/0008-5472.CAN-03-3924

[78] Morgan MJ, Liu ZG. Crosstalk of reactive oxygen species and NF-κB signaling. Cell Res. 2011;21:103–15. 21187859 10.1038/cr.2010.178 PMC3193400

[79] Zhao Y, Hu X, Liu Y, Dong S, Wen Z, He W, Zhang S, Huang Q, Shi M. ROS signaling under metabolic stress: cross-talk between AMPK and AKT pathway. Mol Cancer. 2017;16:79. 28407774 10.1186/s12943-017-0648-1 PMC5390360

[80] Ross D, Siegel D. Functions of NQO1 in Cellular Protection and CoQ10 Metabolism and its Potential Role as a Redox Sensitive Molecular Switch. Front Physiol. 2017;8. 10.3389/fphys.2017.00595. 10.3389/fphys.2017.00595 PMC5573868 28883796

[81] Eijkelenboom A, Burgering BM. FOXOs: signalling integrators for homeostasis maintenance. Nat Rev Mol Cell Biol. 2013;14:83–97. 23325358 10.1038/nrm3507

[82] Rodriguez-Colman MJ, Dansen TB, Burgering BMT. FOXO transcription factors as mediators of stress adaptation. Nat Rev Mol Cell Biol. 2024;25:46–64. 37710009 10.1038/s41580-023-00649-0

[83] Hu W, Zhang C, Wu R, Sun Y, Levine A, Feng Z. Glutaminase 2, a novel p53 target gene regulating energy metabolism and antioxidant function. Proc Natl Acad Sci U S A. 2010;107:7455–60. 20378837 10.1073/pnas.1001006107 PMC2867677

[84] Sablina AA, Budanov AV, Ilyinskaya GV, Agapova LS, Kravchenko JE, Chumakov PM. The antioxidant function of the p53 tumor suppressor. Nat Med. 2005;11:1306–13. 16286925 10.1038/nm1320 PMC2637821

[85] Liu B, Chen Y, St Clair DK. ROS and p53: a versatile partnership. Free Radic Biol Med. 2008;44:1529–35. 18275858 10.1016/j.freeradbiomed.2008.01.011 PMC2359898

[86] Shi T, Dansen TB. Reactive oxygen species induced p53 activation: DNA damage, redox signaling, or both? Antioxid Redox Signal. 2020;33:839–59. 32151151 10.1089/ars.2020.8074

[87] Maillet A, Pervaiz S. Redox regulation of p53, redox effectors regulated by p53: a subtle balance. Antioxid Redox Signal. 2012;16:1285–94. 22117613 10.1089/ars.2011.4434

[88] Lu SC. Glutathione synthesis. Biochim Biophys Acta. 1830;2013:3143–53. 10.1016/j.bbagen.2012.09.008 PMC3549305 22995213

[89] Jones CM, Lawrence A, Wardman P, Burkitt MJ. Electron paramagnetic resonance spin trapping investigation into the kinetics of glutathione oxidation by the superoxide radical: re-evaluation of the rate constant. Free Radic Biol Med. 2002;32:982–90. 12008114 10.1016/s0891-5849(02)00791-8

[90] Luque-Ceballos JC, Rodríguez-Zamora P, López-Olivos JC, Garzón IL. Revisiting the scavenging activity of glutathione: free radicals diversity and reaction mechanisms. Comput Theor Chem. 2023;1227:114227.

[91] Forman HJ, Zhang H, Rinna A. Glutathione: overview of its protective roles, measurement, and biosynthesis. Mol Aspects Med. 2009;30:1–12. 18796312 10.1016/j.mam.2008.08.006 PMC2696075

[92] Litwack G, Ketterer B, Arias IM. Ligandin: a hepatic protein which binds steroids, bilirubin, carcinogens and a number of exogenous organic anions. Nature. 1971;234:466–7. 4944188 10.1038/234466a0

[93] Leaver MJ, George SG. A piscine glutathione S-transferase which efficiently conjugates the end-products of lipid peroxidation. Mar Environ Res. 1998;46:71–4.

[94] Tang D, Kang R, Berghe TV, Vandenabeele P, Kroemer G. The molecular machinery of regulated cell death. Cell Res. 2019;29:347–64. 30948788 10.1038/s41422-019-0164-5 PMC6796845

[95] Xia L, Oyang L, Lin J, Tan S, Han Y, Wu N, Yi P, Tang L, Pan Q, Rao S, et al. The cancer metabolic reprogramming and immune response. Mol Cancer. 2021;20:28. 33546704 10.1186/s12943-021-01316-8 PMC7863491

[96] Xie C, Zhou X, Liang C, Li X, Ge M, Chen Y, Yin J, Zhu J, Zhong C. Apatinib triggers autophagic and apoptotic cell death via VEGFR2/STAT3/PD-L1 and ROS/Nrf2/p62 signaling in lung cancer. J Exp Clin Cancer Res. 2021;40:266. 34429133 10.1186/s13046-021-02069-4 PMC8385858

[97] Zhong L, Arnér ES, Holmgren A. Structure and mechanism of mammalian thioredoxin reductase: the active site is a redox-active selenolthiol/selenenylsulfide formed from the conserved cysteine-selenocysteine sequence. Proc Natl Acad Sci U S A. 2000;97:5854–9. 10801974 10.1073/pnas.100114897 PMC18523

[98] Rhee SG, Kang SW, Chang TS, Jeong W, Kim K. Peroxiredoxin, a novel family of peroxidases. IUBMB Life. 2001;52:35–41. 11795591 10.1080/15216540252774748

[99] Arnér ES, Holmgren A. Physiological functions of thioredoxin and thioredoxin reductase. Eur J Biochem. 2000;267:6102–9. 11012661 10.1046/j.1432-1327.2000.01701.x

[100] Brzozowa-Zasada M, Piecuch A, Bajdak-Rusinek K, Michalski M, Klymenko O, Matysiak N, Janelt K, Czuba Z. Glutathione Reductase Expression and Its Prognostic Significance in Colon Cancer. Int J Mol Sci. 2024;25(2):1097. 10.3390/ijms25021097. 10.3390/ijms25021097 PMC10816751 38256170

[101] Lázaro JJ, Jiménez A, Camejo D, Iglesias-Baena I, Martí Mdel C, Lázaro-Payo A, Barranco-Medina S, Sevilla F. Dissecting the integrative antioxidant and redox systems in plant mitochondria. Effect of stress and S-nitrosylation. Front Plant Sci. 2013;4:460. 24348485 10.3389/fpls.2013.00460 PMC3842906

[102] Becker K, Gromer Shirmer RH, Müller S. Thioredoxin reductase as a pathophysiological factor and drug target. Eur J Biochem. 2000;267(20):6118-25. 10.1046/j.1432-1327.2000.01703.x. 10.1046/j.1432-1327.2000.01703.x 11012663

[103] Qiu J, Zhang T, Zhu X, Yang C, Wang Y, Zhou N, Ju B, Zhou T, Deng G, Qiu C. Hyperoside induces breast cancer cells apoptosis via ROS-mediated NF-κ B signaling pathway. Int J Mol Sci. 2019;21:131. 31878204 10.3390/ijms21010131 PMC6981893

[104] Ivanova D, Zhelev Z, Getsov P, Nikolova B, Aoki I, Higashi T, Bakalova R. Vitamin K: redox-modulation, prevention of mitochondrial dysfunction and anticancer effect. Redox Biol. 2018;16:352–8. 29597144 10.1016/j.redox.2018.03.013 PMC5953218

[105] Hayes JD, Dinkova-Kostova AT, Tew KD. Oxidative stress in cancer. Cancer Cell. 2020;38:167–97. 32649885 10.1016/j.ccell.2020.06.001 PMC7439808

[106] Cox AG, Winterbourn CC, Hampton MB. Mitochondrial peroxiredoxin involvement in antioxidant defence and redox signalling. Biochem J. 2010;425(2):313-25. 10.1042/BJ20091541. 10.1042/BJ20091541 20025614

[107] Cadet J, Delatour T, Douki T, Gasparutto D, Pouget JP, Ravanat JL, Sauvaigo S. Hydroxyl radicals and DNA base damage. Mutat Res. 1999;424:9–21. 10064846 10.1016/s0027-5107(99)00004-4

[108] Somyajit K, Gupta R, Sedlackova H, Neelsen KJ, Ochs F, Rask MB, Choudhary C, Lukas J. Redox-sensitive alteration of replisome architecture safeguards genome integrity. Science. 2017;358:797–802. 29123070 10.1126/science.aao3172

[109] Tretyakova NY, Groehler A, Ji S. DNA-protein cross-links: formation, structural identities, and biological outcomes. Acc Chem Res. 2015;48:1631–44. 26032357 10.1021/acs.accounts.5b00056 PMC4704791

[110] Hayes JD, Dinkova-Kostova AT. The Nrf2 regulatory network provides an interface between redox and intermediary metabolism. Trends Biochem Sci. 2014;39(4):199–218. 10.1016/j.tibs.2014.02.002. 24647116 10.1016/j.tibs.2014.02.002

[111] Song Y, Buettner GR. Thermodynamic and kinetic considerations for the reaction of semiquinone radicals to form superoxide and hydrogen peroxide. Free Radic Biol Med. 2010;49(6):919–62. 10.1016/j.freeradbiomed.2010.05.009. 20493944 10.1016/j.freeradbiomed.2010.05.009 PMC2936108

[112] Bravard A, Vacher M, Gouget B, Coutant A, de Boisferon FH, Marsin S, Chevillard S, Radicella JP. Redox regulation of human OGG1 activity in response to cellular oxidative stress. Mol Cell Biol. 2006;26:7430–6. 16923968 10.1128/MCB.00624-06 PMC1636869

[113] Storz P. Reactive oxygen species in tumor progression. Front Biosci. 2005;10:1881–96. 15769673 10.2741/1667

[114] Ogrunc M, Di Micco R, Liontos M, Bombardelli L, Mione M, Fumagalli M, Gorgoulis VG, d’Adda di Fagagna F. Oncogene-induced reactive oxygen species fuel hyperproliferation and DNA damage response activation. Cell Death Differ. 2014;21:998–1012. 24583638 10.1038/cdd.2014.16 PMC4013514

[115] Renaudin X. Reactive oxygen species and DNA damage response in cancer. Int Rev Cell Mol Biol. 2021;364:139–61. 34507782 10.1016/bs.ircmb.2021.04.001

[116] Wilhelm T, Ragu S, Magdalou I, Machon C, Dardillac E, Técher H, Guitton J, Debatisse M, Lopez BS. Slow Replication Fork Velocity of Homologous Recombination-Defective Cells Results from Endogenous Oxidative Stress. PLOS Genetics. 2016;12(5):e1006007. 10.1371/journal.pgen.1006007. 27135742 10.1371/journal.pgen.1006007 PMC4852921

[117] Petty AJ, Li A, Wang X, Dai R, Heyman B, Hsu D, Huang X, Yang Y. Hedgehog signaling promotes tumor-associated macrophage polarization to suppress intratumoral CD8+ T cell recruitment. J Clin Invest. 2019;129:5151–62. 31638600 10.1172/JCI128644 PMC6877305

[118] Cairns RA, Harris IS, Mak TW. Regulation of cancer cell metabolism. Nat Rev Cancer. 2011;11:85–95. 21258394 10.1038/nrc2981

[119] Panieri E, Santoro MM. ROS homeostasis and metabolism: a dangerous liason in cancer cells. Cell Death Dis. 2016;7:e2253. 27277675 10.1038/cddis.2016.105 PMC5143371

[120] Lou YW, Chen YY, Hsu SF, Chen RK, Lee CL, Khoo KH, Tonks NK, Meng TC. Redox regulation of the protein tyrosine phosphatase PTP1B in cancer cells. FEBS J. 2008;275:69–88. 18067579 10.1111/j.1742-4658.2007.06173.x

[121] Villamar-Cruz O, Loza-Mejía MA, Arias-Romero LE, Camacho-Arroyo I. Recent advances in PTP1B signaling in metabolism and cancer. Biosci Rep. 2021;41:BSR20211994. 34726241 10.1042/BSR20211994 PMC8630396

[122] Haeusler RA, McGraw TE, Accili D. Biochemical and cellular properties of insulin receptor signalling. Nat Rev Mol Cell Biol. 2018;19:31–44. 28974775 10.1038/nrm.2017.89 PMC5894887

[123] Xu Y, Tan M, Tian X, Zhang J, Zhang J, Chen J, Xu W, Sheng H. Leptin receptor mediates the proliferation and glucose metabolism of pancreatic cancer cells via AKT pathway activation. Mol Med Rep. 2020;21:945–52. 31789415 10.3892/mmr.2019.10855

[124] Moody TW, Ramos-Alvarez I, Jensen RT. Peptide G-protein-coupled receptors and ErbB receptor tyrosine kinases in cancer. Biology (Basel). 2023;12:957. 37508387 10.3390/biology12070957 PMC10376828

[125] Son Y, Cheong YK, Kim NH, Chung HT, Kang DG, Pae HO. Mitogen-activated protein kinases and reactive oxygen species: how can ROS activate MAPK pathways? J Signal Transduct. 2011;2011:792639. 21637379 10.1155/2011/792639 PMC3100083

[126] McCubrey JA, Lahair MM, Franklin RA. Reactive oxygen species-induced activation of the MAP kinase signaling pathways. Antioxid Redox Signal. 2006;8:1775–89. 16987031 10.1089/ars.2006.8.1775

[127] Son Y, Kim S, Chung HT, Pae HO. Reactive oxygen species in the activation of MAP kinases. Methods Enzymol. 2013;528:27–48. 23849857 10.1016/B978-0-12-405881-1.00002-1

[128] Giannoni E, Taddei ML, Chiarugi P. Src redox regulation: again in the front line. Free Radic Biol Med. 2010;49:516–27. 20434540 10.1016/j.freeradbiomed.2010.04.025

[129] Guo YJ, Pan WW, Liu SB, Shen ZF, Xu Y, Hu LL. ERK/MAPK signalling pathway and tumorigenesis. Exp Ther Med. 2020;19:1997–2007. 32104259 10.3892/etm.2020.8454 PMC7027163

[130] González-Pacheco FR, Caramelo C, Castilla MA, Deudero JJ, Arias J, Yagüe S, Jiménez S, Bragado R, Alvarez-Arroyo MV. Mechanism of vascular smooth muscle cells activation by hydrogen peroxide: role of phospholipase C gamma. Nephrol Dial Transplant. 2002;17:392–8. 11865083 10.1093/ndt/17.3.392

[131] Kma L, Baruah TJ. The interplay of ROS and the PI3K/Akt pathway in autophagy regulation. Biotechnol Appl Biochem. 2022;69:248–64. 33442914 10.1002/bab.2104

[132] Koundouros N, Poulogiannis G. Phosphoinositide 3-kinase/Akt signaling and redox metabolism in cancer. Front Oncol. 2018;8:160. 29868481 10.3389/fonc.2018.00160 PMC5968394

[133] Moloney JN, Cotter TG. ROS signalling in the biology of cancer. Semin Cell Dev Biol. 2018;80:50–64. 28587975 10.1016/j.semcdb.2017.05.023

[134] Iqbal MJ, Kabeer A, Abbas Z, Siddiqui HA, Calina D, Sharifi-Rad J, Cho WC. Interplay of oxidative stress, cellular communication and signaling pathways in cancer. Cell Commun Signal. 2024;22:7. 38167159 10.1186/s12964-023-01398-5 PMC10763046

[135] Stieg DC, Wang Y, Liu LZ, Jiang BH. ROS and miRNA dysregulation in ovarian cancer development, angiogenesis and therapeutic resistance. Int J Mol Sci. 2022;23:6702. 35743145 10.3390/ijms23126702 PMC9223852

[136] Khromova NV, Kopnin PB, Stepanova EV, Agapova LS, Kopnin BP. p53 hot-spot mutants increase tumor vascularization via ROS-mediated activation of the HIF1/VEGF-A pathway. Cancer Lett. 2009;276:143–51. 19091459 10.1016/j.canlet.2008.10.049

[137] Liu LZ, Hu XW, Xia C, He J, Zhou Q, Shi X, Fang J, Jiang BH. Reactive oxygen species regulate epidermal growth factor-induced vascular endothelial growth factor and hypoxia-inducible factor-1alpha expression through activation of AKT and P70S6K1 in human ovarian cancer cells. Free Radic Biol Med. 2006;41:1521–33. 17045920 10.1016/j.freeradbiomed.2006.08.003

[138] Dewhirst MW, Cao Y, Moeller B. Cycling hypoxia and free radicals regulate angiogenesis and radiotherapy response. Nat Rev Cancer. 2008;8:425–37. 18500244 10.1038/nrc2397 PMC3943205

[139] Ryu JH, Li SH, Park HS, Park JW, Lee B, Chun YS. Hypoxia-inducible factor α subunit stabilization by NEDD8 conjugation is reactive oxygen species-dependent. J Biol Chem. 2011;286:6963–70. 21193393 10.1074/jbc.M110.188706 PMC3044952

[140] Wang T, Dong Y, Huang Z, Zhang G, Zhao Y, Yao H, Hu J, Tüksammel E, Cai H, Liang N, et al. Antioxidants stimulate BACH1-dependent tumor angiogenesis. J Clin Invest. 2023;133:e169671. 37651203 10.1172/JCI169671 PMC10575724

[141] Thiery JP. Epithelial-mesenchymal transitions in tumour progression. Nat Rev Cancer. 2002;2:442–54. 12189386 10.1038/nrc822

[142] Xu J, Lamouille S, Derynck R. TGF-beta-induced epithelial to mesenchymal transition. Cell Res. 2009;19:156–72. 19153598 10.1038/cr.2009.5 PMC4720263

[143] Yazaki K, Matsuno Y, Yoshida K, Sherpa M, Nakajima M, Matsuyama M, Kiwamoto T, Morishima Y, Ishii Y, Hizawa N. ROS-Nrf2 pathway mediates the development of TGF-β1-induced epithelial-mesenchymal transition through the activation of Notch signaling. Eur J Cell Biol. 2021;100:151181. 34763128 10.1016/j.ejcb.2021.151181

[144] Jin M, Wang J, Ji X, Cao H, Zhu J, Chen Y, Yang J, Zhao Z, Ren T, Xing J. MCUR1 facilitates epithelial-mesenchymal transition and metastasis via the mitochondrial calcium dependent ROS/Nrf2/Notch pathway in hepatocellular carcinoma. J Exp Clin Cancer Res. 2019;38:136. 30909929 10.1186/s13046-019-1135-x PMC6434841

[145] Fan Q, Liang X, Xu Z, Li S, Han S, Xiao Y, Xu Q, Yuan R, Yang S, Gao H. Pedunculoside inhibits epithelial-mesenchymal transition and overcomes gefitinib-resistant non-small cell lung cancer through regulating MAPK and Nrf2 pathways. Phytomedicine. 2023;116:154884. 37209605 10.1016/j.phymed.2023.154884

[146] Liao Z, Chua D, Tan NS. Reactive oxygen species: a volatile driver of field cancerization and metastasis. Mol Cancer. 2019;18:65. 30927919 10.1186/s12943-019-0961-y PMC6441160

[147] Chen JR, Zhao JT, Xie ZZ. Integrin-mediated cancer progression as a specific target in clinical therapy. Biomed Pharmacother. 2022;155:113745. 36182738 10.1016/j.biopha.2022.113745

[148] Pi L, Robinson PM, Jorgensen M, Oh SH, Brown AR, Weinreb PH, Trinh TL, Yianni P, Liu C, Leask A, et al. Connective tissue growth factor and integrin αvβ6: a new pair of regulators critical for ductular reaction and biliary fibrosis in mice. Hepatology. 2015;61:678–91. 25203810 10.1002/hep.27425 PMC4303530

[149] Jin M, Seed RI, Cai G, Shing T, Wang L, Ito S, Cormier A, Wankowicz SA, Jespersen JM, Baron JL, et al. Dynamic allostery drives autocrine and paracrine TGF-β signaling. Cell. 2024;187:6200-6219.e6223. 39288764 10.1016/j.cell.2024.08.036 PMC11531391

[150] Chen W, Yang H, Huang L, Fang C, Yao L, Liu F, Jin T. ROS-mediated ITGB5 promotes tongue squamous cell carcinoma metastasis through epithelial mesenchymal transition and cell adhesion signal pathway. J Cancer Res Clin Oncol. 2024;150:398. 39180583 10.1007/s00432-024-05922-z PMC11344732

[151] Jiang J, Wang K, Chen Y, Chen H, Nice EC, Huang C. Redox regulation in tumor cell epithelial-mesenchymal transition: molecular basis and therapeutic strategy. Signal Transduct Target Ther. 2017;2:17036. 29263924 10.1038/sigtrans.2017.36 PMC5661624

[152] Mandal JP, Shiue CN, Chen YC, Lee MC, Yang HH, Chang HH, Hu CT, Liao PC, Hui LC, You RI, Wu WS. PKCδ mediates mitochondrial ROS generation and oxidation of HSP60 to relieve RKIP inhibition on MAPK pathway for HCC progression. Free Radic Biol Med. 2021;163:69–87. 33307168 10.1016/j.freeradbiomed.2020.12.003

[153] Chang H, Li J, Qu K, Wan Y, Liu S, Zheng W, Zhang Z, Liu C. CRIF1 overexpression facilitates tumor growth and metastasis through inducing ROS/NFκB pathway in hepatocellular carcinoma. Cell Death Dis. 2020;11:332. 32382077 10.1038/s41419-020-2528-7 PMC7205899

[154] Tobar N, Villar V, Santibanez JF. ROS-NFkappaB mediates TGF-beta1-induced expression of urokinase-type plasminogen activator, matrix metalloproteinase-9 and cell invasion. Mol Cell Biochem. 2010;340:195–202. 20204677 10.1007/s11010-010-0418-5

[155] Chang CH, Pauklin S. ROS and TGFβ: from pancreatic tumour growth to metastasis. J Exp Clin Cancer Res. 2021;40:152. 33941245 10.1186/s13046-021-01960-4 PMC8091747

[156] Peng F, Xu Q, Jing X, Chi X, Zhang Z, Meng X, Liu X, Yan J, Liu X, Shao S. GPX2 promotes EMT and metastasis in non-small cell lung cancer by activating PI3K/AKT/mTOR/Snail signaling axis. FASEB Bioadv. 2023;5:233–50. 37287867 10.1096/fba.2022-00045 PMC10242197

[157] Chatterjee R, Chatterjee J. ROS and oncogenesis with special reference to EMT and stemness. Eur J Cell Biol. 2020;99:151073. 32201025 10.1016/j.ejcb.2020.151073

[158] Xing Y, Wei X, Liu Y, Wang MM, Sui Z, Wang X, Zhu W, Wu M, Lu C, Fei YH, et al. Autophagy inhibition mediated by MCOLN1/TRPML1 suppresses cancer metastasis via regulating a ROS-driven TP53/p53 pathway. Autophagy. 2022;18:1932–54. 34878954 10.1080/15548627.2021.2008752 PMC9450983

[159] Piskounova E, Agathocleous M, Murphy MM, Hu Z, Huddlestun SE, Zhao Z, Leitch AM, Johnson TM, DeBerardinis RJ, Morrison SJ. Oxidative stress inhibits distant metastasis by human melanoma cells. Nature. 2015;527:186–91. 26466563 10.1038/nature15726 PMC4644103

[160] Wiel C, Le Gal K, Ibrahim MX, Jahangir CA, Kashif M, Yao H, Ziegler DV, Xu X, Ghosh T, Mondal T, et al. BACH1 stabilization by antioxidants stimulates lung cancer metastasis. Cell. 2019;178:330-345.e322. 31257027 10.1016/j.cell.2019.06.005

[161] Ogata FT, Simões Sato AY, Coppo L, Arai RJ, Stern AI, Pequeno Monteiro H. Thiol-based antioxidants and the epithelial/mesenchymal transition in cancer. Antioxid Redox Signal. 2022;36:1037–50. 34541904 10.1089/ars.2021.0199

[162] Lignitto L, LeBoeuf SE, Homer H, Jiang S, Askenazi M, Karakousi TR, Pass HI, Bhutkar AJ, Tsirigos A, Ueberheide B, et al. Nrf2 activation promotes lung cancer metastasis by inhibiting the degradation of Bach1. Cell. 2019;178:316-329.e318. 31257023 10.1016/j.cell.2019.06.003 PMC6625921

[163] Xu H, Chai H, Chen M, Zhu R, Jiang S, Liu X, Wang Y, Chen J, Wei J, Mao Y, Shi Z. Single-cell RNA sequencing identifies a subtype of FN1 + tumor-associated macrophages associated with glioma recurrence and as a biomarker for immunotherapy. Biomark Res. 2024;12:114. 39375795 10.1186/s40364-024-00662-1 PMC11457430

[164] Vadevoo SMP, Kang Y, Gunassekaran GR, Lee SM, Park MS, Jo DG, Kim SK, Lee H, Kim WJ, Lee B. IL4 receptor targeting enables nab-paclitaxel to enhance reprogramming of M2-type macrophages into M1-like phenotype via ROS-HMGB1-TLR4 axis and inhibition of tumor growth and metastasis. Theranostics. 2024;14:2605–21. 38646639 10.7150/thno.92672 PMC11024855

[165] Suzuki S, Venkatesh D, Kanda H, Nakayama A, Hosokawa H, Lee E, Miki T, Stockwell BR, Yokote K, Tanaka T, Prives C. GLS2 is a tumor suppressor and a regulator of ferroptosis in hepatocellular carcinoma. Cancer Res. 2022;82:3209–22. 35895807 10.1158/0008-5472.CAN-21-3914 PMC11057045

[166] Wang Q, Huang L, Yue J. Oxidative stress activates the TRPM2-Ca(2+)-CaMKII-ROS signaling loop to induce cell death in cancer cells. Biochim Biophys Acta Mol Cell Res. 2017;1864:957–67. 28007458 10.1016/j.bbamcr.2016.12.014

[167] Wang S, Chen Z, Zhu S, Lu H, Peng D, Soutto M, Naz H, Peek R Jr, Xu H, Zaika A, et al. PRDX2 protects against oxidative stress induced by H. pylori and promotes resistance to cisplatin in gastric cancer. Redox Biol. 2020;28:101319. 31536951 10.1016/j.redox.2019.101319 PMC6811995

[168] Raha D, Wilson TR, Peng J, Peterson D, Yue P, Evangelista M, Wilson C, Merchant M, Settleman J. The cancer stem cell marker aldehyde dehydrogenase is required to maintain a drug-tolerant tumor cell subpopulation. Cancer Res. 2014;74:3579–90. 24812274 10.1158/0008-5472.CAN-13-3456

[169] He L, Chen J, Deng P, Huang S, Liu P, Wang C, Huang X, Li Y, Chen B, Shi D, et al. Lysosomal cyst(e)ine storage potentiates tolerance to oxidative stress in cancer cells. Mol Cell. 2023;83:3502-3519.e3511. 37751742 10.1016/j.molcel.2023.08.032

[170] Wang X, Zhou T, Yang X, Cao X, Jin G, Zhang P, Guo J, Rong K, Li B, Hu Y, et al. DDRGK1 enhances osteosarcoma chemoresistance via inhibiting KEAP1-mediated NRF2 ubiquitination. Adv Sci (Weinh). 2023;10:e2204438. 36965071 10.1002/advs.202204438 PMC10190621

[171] Ge W, Zhao K, Wang X, Li H, Yu M, He M, Xue X, Zhu Y, Zhang C, Cheng Y, et al. iASPP is an antioxidative factor and drives cancer growth and drug resistance by competing with Nrf2 for Keap1 binding. Cancer Cell. 2017;32:561-573.e566. 29033244 10.1016/j.ccell.2017.09.008

[172] Zhou N, Chen J, Ling Z, Zhang C, Zhou Y, Wang D, Zhou L, Wang Z, Sun N, Wang X, et al. Aryl hydrocarbon receptor sulfenylation promotes glycogenolysis and rescues cancer chemoresistance. J Clin Invest. 2023;133:e170753. 38099490 10.1172/JCI170753 PMC10721154

[173] Zhang L, Xu Y, Cheng Z, Zhao J, Wang M, Sun Y, Mi Z, Yuan Z, Wu Z. The EGR1/miR-139/NRF2 axis orchestrates radiosensitivity of non-small-cell lung cancer via ferroptosis. Cancer Lett. 2024;595:217000. 38821254 10.1016/j.canlet.2024.217000

[174] Adkins I, Fucikova J, Garg AD, Agostinis P, Špíšek R. Physical modalities inducing immunogenic tumor cell death for cancer immunotherapy. Oncoimmunology. 2014;3:e968434. 25964865 10.4161/21624011.2014.968434 PMC4352954

[175] Huang H, Zhang S, Li Y, Liu Z, Mi L, Cai Y, Wang X, Chen L, Ran H, Xiao D, et al. Suppression of mitochondrial ROS by prohibitin drives glioblastoma progression and therapeutic resistance. Nat Commun. 2021;12:3720. 34140524 10.1038/s41467-021-24108-6 PMC8211793

[176] Zhan Y, Zhang Z, Liu Y, Fang Y, Xie Y, Zheng Y, Li G, Liang L, Ding Y. NUPR1 contributes to radiation resistance by maintaining ROS homeostasis via AhR/CYP signal axis in hepatocellular carcinoma. BMC Med. 2022;20:365. 36258210 10.1186/s12916-022-02554-3 PMC9580158

[177] Zhou Y, Zhang J, Gong J, Tang X, Zhang C. UBE2C mediated radiotherapy resistance of head and neck squamous cell carcinoma by regulating oxidative-stress-relative apoptosis. Aging (Albany NY). 2022;14:7003–13. 36069832 10.18632/aging.204265 PMC9512496

[178] Bhattacharya S, Calar K, Evans C, Petrasko M, de la Puente P. Bioengineering the oxygen-deprived tumor microenvironment within a three-dimensional platform for studying tumor-immune interactions. Front Bioeng Biotechnol. 2020;8:1040. 33015012 10.3389/fbioe.2020.01040 PMC7498579

[179] Hanahan D. Hallmarks of cancer: new dimensions. Cancer Discov. 2022;12:31–46. 35022204 10.1158/2159-8290.CD-21-1059

[180] Quail DF, Amulic B, Aziz M, Barnes BJ, Eruslanov E, Fridlender ZG, Goodridge HS, Granot Z, Hidalgo A, Huttenlocher A, et al. Neutrophil phenotypes and functions in cancer: a consensus statement. J Exp Med. 2022;219:e20220011. 35522219 10.1084/jem.20220011 PMC9086501

[181] Leone RD, Powell JD. Metabolism of immune cells in cancer. Nat Rev Cancer. 2020;20:516–31. 32632251 10.1038/s41568-020-0273-y PMC8041116

[182] Jaillon S, Ponzetta A, Di Mitri D, Santoni A, Bonecchi R, Mantovani A. Neutrophil diversity and plasticity in tumour progression and therapy. Nat Rev Cancer. 2020;20:485–503. 32694624 10.1038/s41568-020-0281-y

[183] Toullec A, Gerald D, Despouy G, Bourachot B, Cardon M, Lefort S, Richardson M, Rigaill G, Parrini MC, Lucchesi C, et al. Oxidative stress promotes myofibroblast differentiation and tumour spreading. EMBO Mol Med. 2010;2:211–30. 20535745 10.1002/emmm.201000073 PMC3377319

[184] Zhao Z, Hu Y, Li H, Lu T, He X, Ma Y, Huang M, Li M, Yang L, Shi C. Inhibition of stromal MAOA leading activation of WNT5A enhance prostate cancer immunotherapy by involving the transition of cancer-associated fibroblasts. J Immunother Cancer. 2025;13:e010555. 40121032 10.1136/jitc-2024-010555 PMC11931948

[185] Zhou Z, Qu C, Zhou P, Zhou Q, Li D, Wu X, Yang L. Extracellular vesicles activated cancer-associated fibroblasts promote lung cancer metastasis through mitophagy and mtDNA transfer. J Exp Clin Cancer Res. 2024;43:158. 38825680 10.1186/s13046-024-03077-w PMC11145873

[186] Giannoni E, Bianchini F, Calorini L, Chiarugi P. Cancer associated fibroblasts exploit reactive oxygen species through a proinflammatory signature leading to epithelial mesenchymal transition and stemness. Antioxid Redox Signal. 2011;14:2361–71. 21235356 10.1089/ars.2010.3727

[187] Zhao J, Shen J, Mao L, Yang T, Liu J, Hongbin S. Cancer associated fibroblast secreted miR-432-5p targets CHAC1 to inhibit ferroptosis and promote acquired chemoresistance in prostate cancer. Oncogene. 2024;43(27):2104–14. 38769193 10.1038/s41388-024-03057-6

[188] Gabrilovich DI, Ostrand-Rosenberg S, Bronte V. Coordinated regulation of myeloid cells by tumours. Nat Rev Immunol. 2012;12:253–68. 22437938 10.1038/nri3175 PMC3587148

[189] Gabrilovich DI, Nagaraj S. Myeloid-derived suppressor cells as regulators of the immune system. Nat Rev Immunol. 2009;9:162–74. 19197294 10.1038/nri2506 PMC2828349

[190] Zhao T, Liu S, Hanna NH, Jalal S, Ding X, Wan J, Yan C, Du H. LAL deficiency induced myeloid-derived suppressor cells as targets and biomarkers for lung cancer. J Immunother Cancer. 2023;11:e006272. 36914206 10.1136/jitc-2022-006272 PMC10016256

[191] Beury DW, Carter KA, Nelson C, Sinha P, Hanson E, Nyandjo M, Fitzgerald PJ, Majeed A, Wali N, Ostrand-Rosenberg S. Myeloid-derived suppressor cell survival and function are regulated by the transcription factor Nrf2. J Immunol. 2016;196:3470–8. 26936880 10.4049/jimmunol.1501785 PMC4821672

[192] Jian SL, Chen WW, Su YC, Su YW, Chuang TH, Hsu SC, Huang LR. Glycolysis regulates the expansion of myeloid-derived suppressor cells in tumor-bearing hosts through prevention of ROS-mediated apoptosis. Cell Death Dis. 2017;8:e2779. 28492541 10.1038/cddis.2017.192 PMC5520713

[193] Kusmartsev S, Nefedova Y, Yoder D, Gabrilovich DI. Antigen-specific inhibition of CD8+ T cell response by immature myeloid cells in cancer is mediated by reactive oxygen species. J Immunol. 2004;172:989–99. 14707072 10.4049/jimmunol.172.2.989

[194] Sprouse ML, Welte T, Boral D, Liu HN, Yin W, Vishnoi M, Goswami-Sewell D, Li L, Pei G, Jia P, et al. PMN-MDSCs enhance CTC metastatic properties through reciprocal interactions via ROS/Notch/Nodal signaling. Int J Mol Sci. 1916;2019:20. 10.3390/ijms20081916 PMC6514876 31003475

[195] Xiang H, Ramil CP, Hai J, Zhang C, Wang H, Watkins AA, Afshar R, Georgiev P, Sze MA, Song XS, et al. Cancer-associated fibroblasts promote immunosuppression by inducing ROS-generating monocytic MDSCs in lung squamous cell carcinoma. Cancer Immunol Res. 2020;8:436–50. 32075803 10.1158/2326-6066.CIR-19-0507

[196] Zheng Y, Han Y, Wang T, Liu H, Sun Q, Hu S, Chen J, Li Z. Reprogramming tumor-associated macrophages via ROS-mediated novel mechanism of ultra-small Cu2− xSe nanoparticles to enhance anti-tumor immunity. Adv Func Mater. 2022;32:2108971.

[197] Wang YN, Wang YY, Wang J, Bai WJ, Miao NJ, Wang J. Vinblastine resets tumor-associated macrophages toward M1 phenotype and promotes antitumor immune response. J Immunother Cancer. 2023;11:e007253. 37652576 10.1136/jitc-2023-007253 PMC10476141

[198] Ito M, Mimura K, Nakajima S, Okayama H, Saito K, Nakajima T, Kikuchi T, Onozawa H, Fujita S, Sakamoto W, et al. M2 tumor-associated macrophages resist to oxidative stress through heme oxygenase-1 in the colorectal cancer tumor microenvironment. Cancer Immunol Immunother. 2023;72:2233–44. 36869896 10.1007/s00262-023-03406-6 PMC10992489

[199] Li X, Wang S, Mu W, Barry J, Han A, Carpenter RL, Jiang BH, Peiper SC, Mahoney MG, Aplin AE, et al. Reactive oxygen species reprogram macrophages to suppress antitumor immune response through the exosomal miR-155-5p/PD-L1 pathway. J Exp Clin Cancer Res. 2022;41:41. 35086548 10.1186/s13046-022-02244-1 PMC8793215

[200] Lin X, Zheng W, Liu J, Zhang Y, Qin H, Wu H, Xue B, Lu Y, Shen P. Oxidative stress in malignant melanoma enhances tumor necrosis factor- α secretion of tumor-associated macrophages that promote cancer cell invasion. Antioxid Redox Signal. 2013;19:1337–55. 23373752 10.1089/ars.2012.4617

[201] Yu X, Lao Y, Teng XL, Li S, Zhou Y, Wang F, Guo X, Deng S, Chang Y, Wu X, et al. SENP3 maintains the stability and function of regulatory T cells via BACH2 deSUMOylation. Nat Commun. 2018;9:3157. 30089837 10.1038/s41467-018-05676-6 PMC6082899

[202] Jiang Z, Wang H, Wang X, Duo H, Tao Y, Li J, Li X, Liu J, Ni J, Wu EJ, et al. TMED4 facilitates regulatory T cell suppressive function via ROS homeostasis in tumor and autoimmune mouse models. J Clin Invest. 2024;135:e179874. 39480507 10.1172/JCI179874 PMC11684806

[203] Mougiakakos D, Johansson CC, Kiessling R. Naturally occurring regulatory T cells show reduced sensitivity toward oxidative stress-induced cell death. Blood. 2009;113:3542–5. 19050306 10.1182/blood-2008-09-181040

[204] Maj T, Wang W, Crespo J, Zhang H, Wang W, Wei S, Zhao L, Vatan L, Shao I, Szeliga W, et al. Oxidative stress controls regulatory T cell apoptosis and suppressor activity and PD-L1-blockade resistance in tumor. Nat Immunol. 2017;18:1332–41. 29083399 10.1038/ni.3868 PMC5770150

[205] Steinert EM, Vasan K, Chandel NS. Mitochondrial metabolism regulation of T cell-mediated immunity. Annu Rev Immunol. 2021;39:395–416. 33902315 10.1146/annurev-immunol-101819-082015 PMC10403253

[206] Wu Z, Huang H, Han Q, Hu Z, Teng XL, Ding R, Ye Y, Yu X, Zhao R, Wang Z, Zou Q. SENP7 senses oxidative stress to sustain metabolic fitness and antitum or functions of CD8+ T cells. J Clin Invest. 2022;132:e155224. 35143421 10.1172/JCI155224 PMC8970670

[207] Belikov AV, Schraven B, Simeoni L. TCR-triggered extracellular superoxide production is not required for T-cell activation. Cell Commun Signal. 2014;12:50. 25081034 10.1186/s12964-014-0050-1 PMC4237797

[208] Kennel KB, Greten FR. Immune cell - produced ROS and their impact on tumor growth and metastasis. Redox Biol. 2021;42:101891. 33583736 10.1016/j.redox.2021.101891 PMC8113043

[209] Qin Y, Huo F, Feng Z, Hou J, Ding Y, Wang Q, Gui Y, Yang Z, Yang J, Zhou G, et al. CD36 promotes iron accumulation and dysfunction in CD8+ T Cells via the p38-CEBPB-TfR1 axis in early-stage hepatocellular carcinoma. Clin Mol Hepatol. 2025. 10.3350/cmh.2024.0948 PMC12260632 40037690

[210] Chen S, Fan J, Xie P, Ahn J, Fernandez M, Billingham LK, Miska J, Wu JD, Wainwright DA, Fang D, et al. CD8+ T cells sustain antitumor response by mediating crosstalk between adenosine A2A receptor and glutathione/GPX4. J Clin Invest. 2024;134:e170071. 38441967 10.1172/JCI170071 PMC11014673

[211] Wen Z, Shimojima Y, Shirai T, Li Y, Ju J, Yang Z, Tian L, Goronzy JJ, Weyand CM. NADPH oxidase deficiency underlies dysfunction of aged CD8+ Tregs. J Clin Invest. 2016;126:1953–67. 27088800 10.1172/JCI84181 PMC4855948

[212] Shi C, Zhang Q, Yao Y, Zeng F, Du C, Nijiati S, Wen X, Zhang X, Yang H, Chen H, et al. Targeting the activity of T cells by membrane surface redox regulation for cancer theranostics. Nat Nanotechnol. 2023;18:86–97. 36536041 10.1038/s41565-022-01261-7

[213] Van Acker HH, Ma S, Scolaro T, Kaech SM, Mazzone M. How metabolism bridles cytotoxic CD8(+) T cells through epigenetic modifications. Trends Immunol. 2021;42:401–17. 33867272 10.1016/j.it.2021.03.006 PMC9681987

[214] Qu C, Zhang H, Cao H, Tang L, Mo H, Liu F, Zhang L, Yi Z, Long L, Yan L, et al. Tumor buster - where will the CAR-T cell therapy ‘missile’ go? Mol Cancer. 2022;21:201. 36261831 10.1186/s12943-022-01669-8 PMC9580202

[215] Pu Y, Ji Q. Tumor-associated macrophages regulate PD-1/PD-L1 immunosuppression. Front Immunol. 2022;13:874589. 35592338 10.3389/fimmu.2022.874589 PMC9110638

[216] Wu S, Zhang J, Pan J, Bai S, Wang Z, Chen Y, Xu D, An Y, Liu C, Chu C, et al. Integrated nanorod-mediated PD-L1 downregulation in combination with oxidative-stress immunogene therapy against cancer. Adv Healthc Mater. 2023;12:e2300110. 36773310 10.1002/adhm.202300110

[217] Nakamura K, Matsunaga K. Susceptibility of natural killer (NK) cells to reactive oxygen species (ROS) and their restoration by the mimics of superoxide dismutase (SOD). Cancer Biother Radiopharm. 1998;13:275–90. 10850363 10.1089/cbr.1998.13.275

[218] Stiff A, Trikha P, Mundy-Bosse B, McMichael E, Mace TA, Benner B, Kendra K, Campbell A, Gautam S, Abood D, et al. Nitric oxide production by myeloid-derived suppressor cells plays a role in impairing Fc receptor-mediated natural killer cell function. Clin Cancer Res. 2018;24:1891–904. 29363526 10.1158/1078-0432.CCR-17-0691 PMC7184799

[219] Karlsson V, Stål E, Stoopendahl E, Ivarsson A, Leffler H, Lycke M, Sundqvist M, Sundfeldt K, Christenson K, Bernson E. Elevated Galectin-3 levels in the tumor microenvironment of ovarian cancer - implication of ROS mediated suppression of NK cell antitumor response via tumor-associated neutrophils. Front Immunol. 2024;15:1506236. 39759523 10.3389/fimmu.2024.1506236 PMC11695286

[220] Aydin E, Johansson J, Nazir FH, Hellstrand K, Martner A. Role of NOX2-derived reactive oxygen species in NK cell-mediated control of murine melanoma metastasis. Cancer Immunol Res. 2017;5:804–11. 28760732 10.1158/2326-6066.CIR-16-0382

[221] Jin F, Wu Z, Hu X, Zhang J, Gao Z, Han X, Qin J, Li C, Wang Y. The PI3K/Akt/GSK-3β/ROS/eIF2B pathway promotes breast cancer growth and metastasis via suppression of NK cell cytotoxicity and tumor cell susceptibility. Cancer Biol Med. 2019;16:38–54. 31119045 10.20892/j.issn.2095-3941.2018.0253 PMC6528454

[222] Amin PJ, Shankar BS. Sulforaphane induces ROS mediated induction of NKG2D ligands in human cancer cell lines and enhances susceptibility to NK cell mediated lysis. Life Sci. 2015;126:19–27. 25721293 10.1016/j.lfs.2015.01.026

[223] Liu J, Zhang Y, Yang B, Jia Y, Liu RT, Ding L, Shen Z, Chen X. Synergistic glutathione depletion and STING activation to potentiate dendritic cell maturation and cancer vaccine efficacy. Angew Chem Int Ed Engl. 2024;63:e202318530. 38196070 10.1002/anie.202318530

[224] Hu Z, Teng X-L, Zhang T, Yu X, Ding R, Yi J, Deng L, Wang Z, Zou Q. SENP3 senses oxidative stress to facilitate STING-dependent dendritic cell antitumor function. Mol Cell. 2021;81:940-952.e945. 33434504 10.1016/j.molcel.2020.12.024

[225] Paardekooper LM, Vos W, van den Bogaart G. Oxygen in the tumor microenvironment: effects on dendritic cell function. Oncotarget. 2019;10:883–96. 30783517 10.18632/oncotarget.26608 PMC6368231

[226] Yang Y, Karakhanova S, Hartwig W, D’Haese JG, Philippov PP, Werner J, Bazhin AV. Mitochondria and mitochondrial ROS in cancer: novel targets for anticancer therapy. J Cell Physiol. 2016;231:2570–81. 26895995 10.1002/jcp.25349

[227] Kashif M, Yao H, Schmidt S, Chen X, Truong M, Tüksammel E, Liu Y, Bergo MO. ROS-lowering doses of vitamins C and A accelerate malignant melanoma metastasis. Redox Biol. 2023;60:102619. 36774779 10.1016/j.redox.2023.102619 PMC9945759

[228] Ilghami R, Barzegari A, Mashayekhi MR, Letourneur D, Crepin M, Pavon-Djavid G. The conundrum of dietary antioxidants in cancer chemotherapy. Nutr Rev. 2020;78:65–76. 31407778 10.1093/nutrit/nuz027

[229] Ambrosone CB, Zirpoli GR, Hutson AD, McCann WE, McCann SE, Barlow WE, Kelly KM, Cannioto R, Sucheston-Campbell LE, Hershman DL, et al. Dietary supplement use during chemotherapy and survival outcomes of patients with breast cancer enrolled in a cooperative group clinical trial (SWOG S0221). J Clin Oncol. 2020;38:804–14. 31855498 10.1200/JCO.19.01203 PMC7062457

[230] Block KI, Koch AC, Mead MN, Tothy PK, Newman RA, Gyllenhaal C. Impact of antioxidant supplementation on chemotherapeutic toxicity: a systematic review of the evidence from randomized controlled trials. Int J Cancer. 2008;123:1227–39. 18623084 10.1002/ijc.23754

[231] Lykkesfeldt J, Michels AJ, Frei B. Vitamin C. Adv Nutr. 2014;5:16–8. 24425716 10.3945/an.113.005157 PMC3884093

[232] Du J, Cullen JJ, Buettner GR. Ascorbic acid: chemistry, biology and the treatment of cancer. Biochim Biophys Acta. 2012;1826:443–57. 22728050 10.1016/j.bbcan.2012.06.003 PMC3608474

[233] Reang J, Sharma PC, Thakur VK, Majeed J. Understanding the therapeutic potential of ascorbic acid in the battle to overcome cancer. Biomolecules. 2021;11:1130. 34439796 10.3390/biom11081130 PMC8392841

[234] Jiang T, Mao Y, Ma W, Mao Q, You Y, Yang X, Jiang C, Kang C, Li X, Chen L, et al. CGCG clinical practice guidelines for the management of adult diffuse gliomas. Cancer Lett. 2016;375:263–73. 26966000 10.1016/j.canlet.2016.01.024

[235] Shenoy N, Creagan E, Witzig T, Levine M. Ascorbic acid in cancer treatment: let the phoenix fly. Cancer Cell. 2018;34:700–6. 30174242 10.1016/j.ccell.2018.07.014 PMC6234047

[236] Kaźmierczak-Barańska J, Boguszewska K, Adamus-Grabicka A, Karwowski BT. Two faces of vitamin C-antioxidative and pro-oxidative agent. Nutrients. 2018;12:1501. 10.3390/nu12051501 PMC7285147 32455696

[237] Glorieux C, Buc Calderon P. Vitamin C (ascorbate) and redox topics in cancer. Antioxid Redox Signal. 2021;35:1157–75. 34254829 10.1089/ars.2020.8233

[238] Chen P, Reed G, Jiang J, Wang Y, Sunega J, Dong R, Ma Y, Esparham A, Ferrell R, Levine M, et al. Pharmacokinetic evaluation of intravenous vitamin C: a classic pharmacokinetic study. Clin Pharmacokinet. 2022;61:1237–49. 35750958 10.1007/s40262-022-01142-1 PMC9439974

[239] Fritz H, Flower G, Weeks L, Cooley K, Callachan M, McGowan J, Skidmore B, Kirchner L, Seely D. Intravenous vitamin C and cancer: a systematic review. Integr Cancer Ther. 2014;13:280–300. 24867961 10.1177/1534735414534463

[240] Gordon N, Gallagher PT, Neupane NP, Mandigo AC, McCann JK, Dylgjeri E, Vasilevskaya I, McNair C, Paller CJ, Kelly WK, et al. PARP inhibition and pharmacological ascorbate demonstrate synergy in castration-resistant prostate cancer. bioRxiv. 2023. 10.1002/1878-0261.70183 PMC13238595 41534532

[241] Böttger F, Vallés-Martí A, Cahn L, Jimenez CR. High-dose intravenous vitamin C, a promising multi-targeting agent in the treatment of cancer. J Exp Clin Cancer Res. 2021;40:343. 34717701 10.1186/s13046-021-02134-y PMC8557029

[242] Monti DA, Mitchell E, Bazzan AJ, Littman S, Zabrecky G, Yeo CJ, Pillai MV, Newberg AB, Deshmukh S, Levine M. Phase I evaluation of intravenous ascorbic acid in combination with gemcitabine and erlotinib in patients with metastatic pancreatic cancer. PLoS One. 2012;7:e29794. 22272248 10.1371/journal.pone.0029794 PMC3260161

[243] Welsh JL, Wagner BA, van’t Erve TJ, Zehr PS, Berg DJ, Halfdanarson TR, Yee NS, Bodeker KL, Du J, Roberts LJ 2nd, et al. Pharmacological ascorbate with gemcitabine for the control of metastatic and node-positive pancreatic cancer (PACMAN): results from a phase I clinical trial. Cancer Chemother Pharmacol. 2013;71:765–75. 23381814 10.1007/s00280-013-2070-8 PMC3587047

[244] Chen Q, Espey MG, Sun AY, Pooput C, Kirk KL, Krishna MC, Khosh DB, Drisko J, Levine M. Pharmacologic doses of ascorbate act as a prooxidant and decrease growth of aggressive tumor xenografts in mice. Proc Natl Acad Sci U S A. 2008;105:11105–9. 18678913 10.1073/pnas.0804226105 PMC2516281

[245] Ou J, Zhu X, Chen P, Du Y, Lu Y, Peng X, Bao S, Wang J, Zhang X, Zhang T, Pang CLK. A randomized phase II trial of best supportive care with or without hyperthermia and vitamin C for heavily pretreated, advanced, refractory non-small-cell lung cancer. J Adv Res. 2020;24:175–82. 32368355 10.1016/j.jare.2020.03.004 PMC7190757

[246] Creagan ET, Moertel CG, O’Fallon JR, Schutt AJ, O’Connell MJ, Rubin J, Frytak S. Failure of high-dose vitamin C (ascorbic acid) therapy to benefit patients with advanced cancer. A controlled trial. N Engl J Med. 1979;301:687–90. 384241 10.1056/NEJM197909273011303

[247] Moertel CG, Fleming TR, Creagan ET, Rubin J, O’Connell MJ, Ames MM. High-dose vitamin C versus placebo in the treatment of patients with advanced cancer who have had no prior chemotherapy. A randomized double-blind comparison. N Engl J Med. 1985;312:137–41. 3880867 10.1056/NEJM198501173120301

[248] Poulter JM, White WF, Dickerson JW. Ascorbic acid supplementation and five year survival rates in women with early breast cancer. Acta Vitaminol Enzymol. 1984;6:175–82. 6524577

[249] Verrax J, Calderon PB. Pharmacologic concentrations of ascorbate are achieved by parenteral administration and exhibit antitumoral effects. Free Radic Biol Med. 2009;47:32–40. 19254759 10.1016/j.freeradbiomed.2009.02.016

[250] Chen Q, Espey MG, Krishna MC, Mitchell JB, Corpe CP, Buettner GR, Shacter E, Levine M. Pharmacologic ascorbic acid concentrations selectively kill cancer cells: action as a pro-drug to deliver hydrogen peroxide to tissues. Proc Natl Acad Sci U S A. 2005;102:13604–9. 16157892 10.1073/pnas.0506390102 PMC1224653

[251] Espey MG, Chen P, Chalmers B, Drisko J, Sun AY, Levine M, Chen Q. Pharmacologic ascorbate synergizes with gemcitabine in preclinical models of pancreatic cancer. Free Radic Biol Med. 2011;50:1610–9. 21402145 10.1016/j.freeradbiomed.2011.03.007 PMC3482496

[252] Serrano OK, Parrow NL, Violet PC, Yang J, Zornjak J, Basseville A, Levine M. Antitumor effect of pharmacologic ascorbate in the B16 murine melanoma model. Free Radic Biol Med. 2015;87:193–203. 26119785 10.1016/j.freeradbiomed.2015.06.032

[253] Xia J, Xu H, Zhang X, Allamargot C, Coleman KL, Nessler R, Frech I, Tricot G, Zhan F. Multiple myeloma tumor cells are selectively killed by pharmacologically-dosed ascorbic acid. EBioMedicine. 2017;18:41–9. 28229908 10.1016/j.ebiom.2017.02.011 PMC5405162

[254] Omenn GS, Goodman GE, Thornquist MD, Balmes J, Cullen MR, Glass A, Keogh JP, Meyskens FL, Valanis B, Williams JH, et al. Effects of a combination of beta carotene and vitamin A on lung cancer and cardiovascular disease. N Engl J Med. 1996;334:1150–5. 8602180 10.1056/NEJM199605023341802

[255] Ramchatesingh B, Martínez Villarreal A, Arcuri D, Lagacé F, Setah SA, Touma F, Al-Badarin F, Litvinov IV. The use of retinoids for the prevention and treatment of skin cancers: an updated review. Int J Mol Sci. 2022;23:12622. 36293471 10.3390/ijms232012622 PMC9603842

[256] Kim JA, Jang JH, Lee SY. An updated comprehensive review on vitamin A and carotenoids in breast cancer: mechanisms, genetics, assessment, current evidence, and future clinical implications. Nutrients. 2021;13:3162. 34579037 10.3390/nu13093162 PMC8465379

[257] van Zandwijk N, Dalesio O, Pastorino U, de Vries N, van Tinteren H. EUROSCAN, a randomized trial of vitamin A and N-acetylcysteine in patients with head and neck cancer or lung cancer. For the EUropean Organization for Research and Treatment of Cancer Head and Neck and Lung Cancer Cooperative Groups. J Natl Cancer Inst. 2000;92:977–86. 10861309 10.1093/jnci/92.12.977

[258] Sayin VI, Ibrahim MX, Larsson E, Nilsson JA, Lindahl P, Bergo MO. Antioxidants accelerate lung cancer progression in mice. Sci Transl Med. 2014;6:221ra215. 10.1126/scitranslmed.3007653 24477002

[259] Xin J, Jiang X, Ben S, Yuan Q, Su L, Zhang Z, Christiani DC, Du M, Wang M. Association between circulating vitamin E and ten common cancers: evidence from large-scale Mendelian randomization analysis and a longitudinal cohort study. BMC Med. 2022;20:168. 35538486 10.1186/s12916-022-02366-5 PMC9092790

[260] Lippman SM, Klein EA, Goodman PJ, Lucia MS, Thompson IM, Ford LG, Parnes HL, Minasian LM, Gaziano JM, Hartline JA, et al. Effect of selenium and vitamin E on risk of prostate cancer and other cancers: the Selenium and Vitamin E Cancer Prevention Trial (SELECT). JAMA. 2009;301:39–51. 19066370 10.1001/jama.2008.864 PMC3682779

[261] Pierpaoli E, Viola V, Barucca A, Orlando F, Galli F, Provinciali M. Effect of annatto-tocotrienols supplementation on the development of mammary tumors in HER-2/neu transgenic mice. Carcinogenesis. 2013;34:1352–60. 23430951 10.1093/carcin/bgt064

[262] Copat C, Favara C, Tomasello MF, Sica C, Grasso A, Dominguez HG, Conti GO, Ferrante M. Astaxanthin in cancer therapy and prevention (review). Biomed Rep. 2025;22:66. 40017498 10.3892/br.2025.1944 PMC11865706

[263] Chew EY, Clemons TE, Agrón E, Domalpally A, Keenan TDL, Vitale S, Weber C, Smith DC, Christen W. Long-term outcomes of adding lutein/zeaxanthin and ω-3 fatty acids to the AREDS supplements on age-related macular degeneration progression: AREDS2 report 28. JAMA Ophthalmol. 2022;140:692–8. 35653117 10.1001/jamaophthalmol.2022.1640 PMC9164119

[264] Gong X, Smith JR, Swanson HM, Rubin LP. Carotenoid lutein selectively inhibits breast cancer cell growth and potentiates the effect of chemotherapeutic agents through ROS-mediated mechanisms. Molecules. 2018;23:905. 29662002 10.3390/molecules23040905 PMC6017803

[265] Paur I, Lilleby W, Bøhn SK, Hulander E, Klein W, Vlatkovic L, Axcrona K, Bolstad N, Bjøro T, Laake P, et al. Tomato-based randomized controlled trial in prostate cancer patients: effect on PSA. Clin Nutr. 2017;36:672–9. 27406859 10.1016/j.clnu.2016.06.014

[266] Zhou Y, Fu R, Yang M, Liu W, Tong Z. Lycopene suppresses gastric cancer cell growth without affecting normal gastric epithelial cells. J Nutr Biochem. 2023;116:109313. 36871837 10.1016/j.jnutbio.2023.109313

[267] Alhoshani NM, Al-Zharani M, Almutairi B, Aljarba NH, Al-Johani NS, Alkeraishan N, AlKahtane AA, Alarifi S, Ali D, Alkahtani S. Antioxidant and anti-inflammatory activities of lycopene against 5-fluorouracil-induced cytotoxicity in Caco2 cells. Saudi Pharm J. 2022;30:1665–71. 36465840 10.1016/j.jsps.2022.09.011 PMC9715638

[268] Thyagarajan A, Forino AS, Konger RL, Sahu RP. Dietary polyphenols in cancer chemoprevention: implications in pancreatic cancer. Antioxidants (Basel). 2020;9:651. 32717779 10.3390/antiox9080651 PMC7464582

[269] Suganuma M, Okabe S, Kai Y, Sueoka N, Sueoka E, Fujiki H. Synergistic effects of (–)-epigallocatechin gallate with (–)-epicatechin, sulindac, or tamoxifen on cancer-preventive activity in the human lung cancer cell line PC-9. Cancer Res. 1999;59:44–7. 9892181

[270] Suganuma M, Sueoka E, Sueoka N, Okabe S, Fujiki H. Mechanisms of cancer prevention by tea polyphenols based on inhibition of TNF-alpha expression. BioFactors. 2000;13:67–72. 11237202 10.1002/biof.5520130112

[271] Lee MH, Han DW, Hyon SH, Park JC. Apoptosis of human fibrosarcoma HT-1080 cells by epigallocatechin-3-O-gallate via induction of p53 and caspases as well as suppression of Bcl-2 and phosphorylated nuclear factor-κB. Apoptosis. 2011;16:75–85. 20963498 10.1007/s10495-010-0548-y

[272] Maeda-Yamamoto M, Suzuki N, Sawai Y, Miyase T, Sano M, Hashimoto-Ohta A, Isemura M. Association of suppression of extracellular signal-regulated kinase phosphorylation by epigallocatechin gallate with the reduction of matrix metalloproteinase activities in human fibrosarcoma HT1080 cells. J Agric Food Chem. 2003;51:1858–63. 12643642 10.1021/jf021039l

[273] Maeda-Yamamoto M, Kawahara H, Tahara N, Tsuji K, Hara Y, Isemura M. Effects of tea polyphenols on the invasion and matrix metalloproteinases activities of human fibrosarcoma HT1080 cells. J Agric Food Chem. 1999;47:2350–4. 10794635 10.1021/jf9811525

[274] Bodduluru LN, Kasala ER, Barua CC, Karnam KC, Dahiya V, Ellutla M. Antiproliferative and antioxidant potential of hesperetin against benzo(a)pyrene-induced lung carcinogenesis in Swiss albino mice. Chem Biol Interact. 2015;242:345–52. 26546711 10.1016/j.cbi.2015.10.020

[275] Bodduluru LN, Kasala ER, Madhana RM, Barua CC, Hussain MI, Haloi P, Borah P. Naringenin ameliorates inflammation and cell proliferation in benzo(a)pyrene induced pulmonary carcinogenesis by modulating CYP1A1, NFκB and PCNA expression. Int Immunopharmacol. 2016;30:102–10. 26655880 10.1016/j.intimp.2015.11.036

[276] Iida K, Naiki T, Naiki-Ito A, Suzuki S, Kato H, Nozaki S, Nagai T, Etani T, Nagayasu Y, Ando R, et al. Luteolin suppresses bladder cancer growth via regulation of mechanistic target of rapamycin pathway. Cancer Sci. 2020;111:1165–79. 31994822 10.1111/cas.14334 PMC7156788

[277] Kang KA, Piao MJ, Ryu YS, Hyun YJ, Park JE, Shilnikova K, Zhen AX, Kang HK, Koh YS, Jeong YJ, Hyun JW. Luteolin induces apoptotic cell death via antioxidant activity in human colon cancer cells. Int J Oncol. 2017;51:1169–78. 28791416 10.3892/ijo.2017.4091

[278] Leung HW, Kuo CL, Yang WH, Lin CH, Lee HZ. Antioxidant enzymes activity involvement in luteolin-induced human lung squamous carcinoma CH27 cell apoptosis. Eur J Pharmacol. 2006;534:12–8. 16469309 10.1016/j.ejphar.2006.01.021

[279] Granato M, Gilardini Montani MS, Santarelli R, D’Orazi G, Faggioni A, Cirone M. Apigenin, by activating p53 and inhibiting STAT3, modulates the balance between pro-apoptotic and pro-survival pathways to induce PEL cell death. J Exp Clin Cancer Res. 2017;36:167. 29179721 10.1186/s13046-017-0632-z PMC5704516

[280] Alhosin M, León-González AJ, Dandache I, Lelay A, Rashid SK, Kevers C, Pincemail J, Fornecker LM, Mauvieux L, Herbrecht R, Schini-Kerth VB. Bilberry extract (Antho 50) selectively induces redox-sensitive caspase 3-related apoptosis in chronic lymphocytic leukemia cells by targeting the Bcl-2/Bad pathway. Sci Rep. 2015;5:8996. 25757575 10.1038/srep08996 PMC4355738

[281] Cvorovic J, Tramer F, Granzotto M, Candussio L, Decorti G, Passamonti S. Oxidative stress-based cytotoxicity of delphinidin and cyanidin in colon cancer cells. Arch Biochem Biophys. 2010;501:151–7. 20494645 10.1016/j.abb.2010.05.019

[282] Lee HZ, Lin CJ, Yang WH, Leung WC, Chang SP. Aloe-emodin induced DNA damage through generation of reactive oxygen species in human lung carcinoma cells. Cancer Lett. 2006;239:55–63. 16300878 10.1016/j.canlet.2005.07.036

[283] León-González AJ, Auger C, Schini-Kerth VB. Pro-oxidant activity of polyphenols and its implication on cancer chemoprevention and chemotherapy. Biochem Pharmacol. 2015;98:371–80. 26206193 10.1016/j.bcp.2015.07.017

[284] Yuan X, Zhang B, Gan L, Wang ZH, Yu BC, Liu LL, Zheng QS, Wang ZP. Involvement of the mitochondrion-dependent and the endoplasmic reticulum stress-signaling pathways in isoliquiritigenin-induced apoptosis of HeLa cell. Biomed Environ Sci. 2013;26:268–76. 23534467 10.3967/0895-3988.2013.04.005

[285] Hwang JT, Ha J, Park IJ, Lee SK, Baik HW, Kim YM, Park OJ. Apoptotic effect of EGCG in HT-29 colon cancer cells via AMPK signal pathway. Cancer Lett. 2007;247:115–21. 16797120 10.1016/j.canlet.2006.03.030

[286] Palit S, Kar S, Sharma G, Das PK. Hesperetin induces apoptosis in breast carcinoma by triggering accumulation of ROS and activation of ASK1/JNK pathway. J Cell Physiol. 2015;230:1729–39. 25204891 10.1002/jcp.24818

[287] Kim GT, Lee SH, Kim YM. Quercetin regulates sestrin 2-AMPK-mTOR signaling pathway and induces apoptosis via increased intracellular ROS in HCT116 colon cancer cells. J Cancer Prev. 2013;18:264–70. 25337554 10.15430/JCP.2013.18.3.264 PMC4189461

[288] Zhang Q, Cheng G, Qiu H, Zhu L, Ren Z, Zhao W, Zhang T, Liu L. The p53-inducible gene 3 involved in flavonoid-induced cytotoxicity through the reactive oxygen species-mediated mitochondrial apoptotic pathway in human hepatoma cells. Food Funct. 2015;6:1518–25. 25820747 10.1039/c5fo00142k

[289] Jin S, Zhang QY, Kang XM, Wang JX, Zhao WH. Daidzein induces MCF-7 breast cancer cell apoptosis via the mitochondrial pathway. Ann Oncol. 2010;21:263–8. 19889614 10.1093/annonc/mdp499

[290] Lo YL, Wang W, Ho CT. 7,3’,4’-Trihydroxyisoflavone modulates multidrug resistance transporters and induces apoptosis via production of reactive oxygen species. Toxicology. 2012;302:221–32. 22914566 10.1016/j.tox.2012.08.003

[291] Rakshit S, Mandal L, Pal BC, Bagchi J, Biswas N, Chaudhuri J, Chowdhury AA, Manna A, Chaudhuri U, Konar A, et al. Involvement of ROS in chlorogenic acid-induced apoptosis of Bcr-Abl+ CML cells. Biochem Pharmacol. 2010;80:1662–75. 20832390 10.1016/j.bcp.2010.08.013

[292] Kim KK, Singh AP, Singh RK, Demartino A, Brard L, Vorsa N, Lange TS, Moore RG. Anti-angiogenic activity of cranberry proanthocyanidins and cytotoxic properties in ovarian cancer cells. Int J Oncol. 2012;40:227–35. 21922132 10.3892/ijo.2011.1198

[293] Gundala SR, Yang C, Mukkavilli R, Paranjpe R, Brahmbhatt M, Pannu V, Cheng A, Reid MD, Aneja R. Hydroxychavicol, a betel leaf component, inhibits prostate cancer through ROS-driven DNA damage and apoptosis. Toxicol Appl Pharmacol. 2014;280:86–96. 25064160 10.1016/j.taap.2014.07.012 PMC4363134

[294] Rana C, Piplani H, Vaish V, Nehru B, Sanyal SN. Downregulation of PI3-K/Akt/PTEN pathway and activation of mitochondrial intrinsic apoptosis by diclofenac and curcumin in colon cancer. Mol Cell Biochem. 2015;402:225–41. 25644785 10.1007/s11010-015-2330-5

[295] Luo C, Li Y, Wang H, Cui Y, Feng Z, Li H, Li Y, Wang Y, Wurtz K, Weber P, et al. Hydroxytyrosol promotes superoxide production and defects in autophagy leading to anti-proliferation and apoptosis on human prostate cancer cells. Curr Cancer Drug Targets. 2013;13:625–39. 23597197 10.2174/15680096113139990035

[296] Sun L, Luo C, Liu J. Hydroxytyrosol induces apoptosis in human colon cancer cells through ROS generation. Food Funct. 2014;5:1909–14. 24953710 10.1039/c4fo00187g

[297] Guha P, Dey A, Sen R, Chatterjee M, Chattopadhyay S, Bandyopadhyay SK. Intracellular GSH depletion triggered mitochondrial Bax translocation to accomplish resveratrol-induced apoptosis in the U937 cell line. J Pharmacol Exp Ther. 2011;336:206–14. 20876229 10.1124/jpet.110.171983

[298] Balansky R, Ganchev G, Iltcheva M, Steele VE, De Flora S. Prevention of cigarette smoke-induced lung tumors in mice by budesonide, phenethyl isothiocyanate, and N-acetylcysteine. Int J Cancer. 2010;126:1047–54. 19816928 10.1002/ijc.24942 PMC4909837

[299] Conaway CC, Jiao D, Kelloff GJ, Steele VE, Rivenson A, Chung FL. Chemopreventive potential of fumaric acid, N-acetylcysteine, N-(4-hydroxyphenyl) retinamide and beta-carotene for tobacco-nitrosamine-induced lung tumors in A/J mice. Cancer Lett. 1998;124:85–93. 9500196 10.1016/s0304-3835(97)00454-0

[300] Kwon Y. Possible beneficial effects of N-acetylcysteine for treatment of triple-negative breast cancer. Antioxidants (Basel). 2021;10:169. 33498875 10.3390/antiox10020169 PMC7911701

[301] Le Gal K, Ibrahim MX, Wiel C, Sayin VI, Akula MK, Karlsson C, Dalin MG, Akyürek LM, Lindahl P, Nilsson J, Bergo MO. Antioxidants can increase melanoma metastasis in mice. Sci Transl Med. 2015;7:308re308. 10.1126/scitranslmed.aad3740 26446958

[302] Capeloa T, Van de Velde JA, d’Hose D, Lipari SG, Derouane F, Hamelin L, Bedin M, Vazeille T, Duhoux FP, Murphy MP, et al. Inhibition of mitochondrial redox signaling with MitoQ prevents metastasis of human pancreatic cancer in mice. Cancers (Basel). 2022;14:4918. 36230841 10.3390/cancers14194918 PMC9562676

[303] Le Gal K, Wiel C, Ibrahim MX, Henricsson M, Sayin VI, Bergo MO. Mitochondria-targeted antioxidants MitoQ and MitoTEMPO do not influence BRAF-driven malignant melanoma and KRAS-driven lung cancer progression in mice. Antioxidants (Basel). 2021;10:163. 33499262 10.3390/antiox10020163 PMC7912553

[304] Titova E, Shagieva G, Ivanova O, Domnina L, Domninskaya M, Strelkova O, Khromova N, Kopnin P, Chernyak B, Skulachev V, Dugina V. Mitochondria-targeted antioxidant SkQ1 suppresses fibrosarcoma and rhabdomyosarcoma tumour cell growth. Cell Cycle. 2018;17:1797–811. 29995559 10.1080/15384101.2018.1496748 PMC6133338

[305] Bazhin AV, Yang Y, D’Haese JG, Werner J, Philippov PP, Karakhanova S. The novel mitochondria-targeted antioxidant SkQ1 modulates angiogenesis and inflammatory micromilieu in a murine orthotopic model of pancreatic cancer. Int J Cancer. 2016;139:130–9. 26914404 10.1002/ijc.30054

[306] Jiang Z, Fletcher NM, Ali-Fehmi R, Diamond MP, Abu-Soud HM, Munkarah AR, Saed GM. Modulation of redox signaling promotes apoptosis in epithelial ovarian cancer cells. Gynecol Oncol. 2011;122:418–23. 21620448 10.1016/j.ygyno.2011.04.051 PMC4237166

[307] Doroshow JH, Gaur S, Markel S, Lu J, van Balgooy J, Synold TW, Xi B, Wu X, Juhasz A. Effects of iodonium-class flavin dehydrogenase inhibitors on growth, reactive oxygen production, cell cycle progression, NADPH oxidase 1 levels, and gene expression in human colon cancer cells and xenografts. Free Radic Biol Med. 2013;57:162–75. 23314043 10.1016/j.freeradbiomed.2013.01.002 PMC3594408

[308] Garrido-Urbani S, Jemelin S, Deffert C, Carnesecchi S, Basset O, Szyndralewiez C, Heitz F, Page P, Montet X, Michalik L, et al. Targeting vascular NADPH oxidase 1 blocks tumor angiogenesis through a PPARα mediated mechanism. PLoS One. 2011;6:e14665. 21326871 10.1371/journal.pone.0014665 PMC3034713

[309] Demircan MB, Mgbecheta PC, Kresinsky A, Schnoeder TM, Schröder K, Heidel FH, Böhmer FD. Combined activity of the redox-modulating compound setanaxib (GKT137831) with cytotoxic agents in the killing of acute myeloid leukemia cells. Antioxidants (Basel). 2022;11:513. 35326163 10.3390/antiox11030513 PMC8944474

[310] Rabbani ZN, Spasojevic I, Zhang X, Moeller BJ, Haberle S, Vasquez-Vivar J, Dewhirst MW, Vujaskovic Z, Batinic-Haberle I. Antiangiogenic action of redox-modulating Mn(III) meso-tetrakis(N-ethy lpyridinium-2-yl)porphyrin, MnTE-2-PyP(5+), via suppression of oxidative stress in a mouse model of breast tumor. Free Radic Biol Med. 2009;47:992–1004. 19591920 10.1016/j.freeradbiomed.2009.07.001 PMC2749298

[311] Costa JG, Saraiva N, Batinic-Haberle I, Castro M, Oliveira NG, Fernandes AS. The SOD mimic MnTnHex-2-PyP^5+^ reduces the viability and migration of 786-O human renal cancer cells. Antioxidants (Basel). 2019;8:490. 31627290 10.3390/antiox8100490 PMC6826590

[312] Manciu FS, Guerrero J, Bennet KE, Chang SY, Rahman M, Martinez Lopez LV, Chantigian S, Castellanos M, Manciu M. Assessing nordihydroguaiaretic acid therapeutic effect for glioblastoma multiforme. Sensors (Basel). 2022;22:2643. 35408257 10.3390/s22072643 PMC9002887

[313] Rahman S, Ansari RA, Rehman H, Parvez S, Raisuddin S. Nordihydroguaiaretic acid from creosote bush (Larrea tridentata) mitigates 12-O-tetradecanoylphorbol-13-acetate-induced inflammatory and oxidative stress responses of tumor promotion cascade in mouse skin. Evid Based Complement Alternat Med. 2011;2011:734785. 19861506 10.1093/ecam/nep076 PMC3138708

[314] Graff JR, McNulty AM, Hanna KR, Konicek BW, Lynch RL, Bailey SN, Banks C, Capen A, Goode R, Lewis JE, et al. The protein kinase Cbeta-selective inhibitor, enzastaurin (LY317615.HCl), suppresses signaling through the AKT pathway, induces apoptosis, and suppresses growth of human colon cancer and glioblastoma xenografts. Cancer Res. 2005;65:7462–9. 16103100 10.1158/0008-5472.CAN-05-0071

[315] Messerli SM, Schaefer AM, Zhuang Y, Soltys BJ, Keime N, Jin J, Ma L, Hsia CJC, Miskimins WK. Use of antimetastatic SOD3-mimetic albumin as a primer in triple negative breast cancer. J Oncol. 2019;2019:3253696. 30941174 10.1155/2019/3253696 PMC6420975

[316] Cohen-Nowak AJ, Cohen AJ, Correia ED, Portocarrero CP, South AP, Nikbakht N. Omaveloxolone attenuates squamous cell carcinoma growth and disease severity in an epidermolysis bullosa mouse model. Exp Dermatol. 2022;31:1083–8. 35285087 10.1111/exd.14564

[317] Chen K, Wu S, Ye S, Huang H, Zhou Y, Zhou H, Wu S, Mao Y, Shangguan F, Lan L, Chen B. Dimethyl fumarate induces metabolic crisie to suppress pancreatic carcinoma. Front Pharmacol. 2021;12:617714. 33692690 10.3389/fphar.2021.617714 PMC7937954

[318] Gonnella R, Zarrella R, Santarelli R, Germano CA, Gilardini Montani MS, Cirone M. Mechanisms of sensitivity and resistance of primary effusion lymphoma to dimethyl fumarate (DMF). Int J Mol Sci. 2022;23:6773. 35743211 10.3390/ijms23126773 PMC9223506

[319] da Silva IV, Pimpão C, Paccetti-Alves I, Thomas SR, Barateiro A, Casini A, Soveral G. Blockage of aquaporin-3 peroxiporin activity by organogold compounds affects melanoma cell adhesion, proliferation and migration. J Physiol. 2024;602:3111–29. 38323926 10.1113/JP284155

[320] Huang P, Åbacka H, Wilson CJ, Wind ML, Rűtzler M, Hagström-Andersson A, Gourdon P, de Groot BL, Venskutonytė R, Lindkvist-Petersson K. Molecular basis for human aquaporin inhibition. Proc Natl Acad Sci U S A. 2024;121:e2319682121. 38319972 10.1073/pnas.2319682121 PMC10873552

[321] Kourghi M, Pei JV, De Ieso ML, Flynn G, Yool AJ. Bumetanide derivatives AqB007 and AqB011 selectively block the aquaporin-1 ion channel conductance and slow cancer cell migration. Mol Pharmacol. 2016;89:133–40. 26467039 10.1124/mol.115.101618 PMC6067643

[322] Smith E, Palethorpe HM, Tomita Y, Pei JV, Townsend AR, Price TJ, Young JP, Yool AJ, Hardingham JE. The purified extract from the medicinal plant Bacopa monnieri, Bacopaside II, inhibits growth of colon cancer cells in vitro by inducing cell cycle arrest and apoptosis. Cells. 2018;7:81. 30037060 10.3390/cells7070081 PMC6070819

[323] Blaner WS, Shmarakov IO, Traber MG. Vitamin A and vitamin E: will the real antioxidant please stand up? Annu Rev Nutr. 2021;41:105–31. 34115520 10.1146/annurev-nutr-082018-124228

[324] Donnelly J, Appathurai A, Yeoh HL, Driscoll K, Faisal W. Vitamin E in cancer treatment: a review of clinical applications in randomized control trials. Nutrients. 2022;14:4329. 36297013 10.3390/nu14204329 PMC9611110

[325] Jomova K, Alomar SY, Alwasel SH, Nepovimova E, Kuca K, Valko M. Several lines of antioxidant defense against oxidative stress: antioxidant enzymes, nanomaterials with multiple enzyme-mimicking activities, and low-molecular-weight antioxidants. Arch Toxicol. 2024;98:1323–67. 38483584 10.1007/s00204-024-03696-4 PMC11303474

[326] Michaud DS, Pietinen P, Taylor PR, Virtanen M, Virtamo J, Albanes D. Intakes of fruits and vegetables, carotenoids and vitamins A, E, C in relation to the risk of bladder cancer in the ATBC cohort study. Br J Cancer. 2002;87:960–5. 12434284 10.1038/sj.bjc.6600604 PMC2364321

[327] Peters U, Leitzmann MF, Chatterjee N, Wang Y, Albanes D, Gelmann EP, Friesen MD, Riboli E, Hayes RB. Serum lycopene, other carotenoids, and prostate cancer risk: a nested case-control study in the prostate, lung, colorectal, and ovarian cancer screening trial. Cancer Epidemiol Biomarkers Prev. 2007;16:962–8. 17507623 10.1158/1055-9965.EPI-06-0861

[328] Datta M, Shaw EG, Lesser GJ, Case LD, Vitolins MZ, Schneider C, Frizzell B, Sullivan C, Lively M, Franzmann E, Hu JJ. A randomized double-blind placebo-controlled trial of fruit and vegetable concentrates on intermediate biomarkers in head and neck cancer. Integr Cancer Ther. 2018;17:115–23. 28102098 10.1177/1534735416684947 PMC5501769

[329] Donoso A, González-Durán J, Muñoz AA, González PA, Agurto-Muñoz C. Therapeutic uses of natural astaxanthin: an evidence-based review focused on human clinical trials. Pharmacol Res. 2021;166:105479. 33549728 10.1016/j.phrs.2021.105479

[330] Levy J, Bosin E, Feldman B, Giat Y, Miinster A, Danilenko M, Sharoni Y. Lycopene is a more potent inhibitor of human cancer cell proliferation than either alpha-carotene or beta-carotene. Nutr Cancer. 1995;24:257–66. 8610045 10.1080/01635589509514415

[331] Kapała A, Szlendak M, Motacka E. The anti-cancer activity of lycopene: a systematic review of human and animal studies. Nutrients. 2022;14:5152. 36501182 10.3390/nu14235152 PMC9741066

[332] Lilly MB, Wu C, Ke Y, Chen WP, Soloff AC, Armeson K, Yokoyama NN, Li X, Song L, Yuan Y, et al. A phase I study of docetaxel plus synthetic lycopene in metastatic prostate cancer patients. Clin Transl Med. 2024;14:e1627. 38515274 10.1002/ctm2.1627 PMC10958125

[333] Lyubitelev A, Studitsky V. Inhibition of cancer development by natural plant polyphenols: molecular mechanisms. Int J Mol Sci. 2023;24:10663. 37445850 10.3390/ijms241310663 PMC10341686

[334] Pietta PG. Flavonoids as antioxidants. J Nat Prod. 2000;63:1035–42. 10924197 10.1021/np9904509

[335] Wu SX, Xiong RG, Huang SY, Zhou DD, Saimaiti A, Zhao CN, Shang A, Zhang YJ, Gan RY, Li HB. Effects and mechanisms of resveratrol for prevention and management of cancers: an updated review. Crit Rev Food Sci Nutr. 2023;63:12422–40. 35852215 10.1080/10408398.2022.2101428

[336] Howells LM, Berry DP, Elliott PJ, Jacobson EW, Hoffmann E, Hegarty B, Brown K, Steward WP, Gescher AJ. Phase I randomized, double-blind pilot study of micronized resveratrol (SRT501) in patients with hepatic metastases–safety, pharmacokinetics, and pharmacodynamics. Cancer Prev Res (Phila). 2011;4:1419–25. 21680702 10.1158/1940-6207.CAPR-11-0148 PMC3173869

[337] Chow HH, Garland LL, Heckman-Stoddard BM, Hsu CH, Butler VD, Cordova CA, Chew WM, Cornelison TL. A pilot clinical study of resveratrol in postmenopausal women with high body mass index: effects on systemic sex steroid hormones. J Transl Med. 2014;12:223. 25115686 10.1186/s12967-014-0223-0 PMC4243716

[338] Nguyen AV, Martinez M, Stamos MJ, Moyer MP, Planutis K, Hope C, Holcombe RF. Results of a phase I pilot clinical trial examining the effect of plant-derived resveratrol and grape powder on Wnt pathway target gene expression in colonic mucosa and colon cancer. Cancer Manag Res. 2009;1:25–37. 21188121 PMC3004662

[339] Slika H, Mansour H, Wehbe N, Nasser SA, Iratni R, Nasrallah G, Shaito A, Ghaddar T, Kobeissy F, Eid AH. Therapeutic potential of flavonoids in cancer: ROS-mediated mechanisms. Biomed Pharmacother. 2022;146:112442. 35062053 10.1016/j.biopha.2021.112442

[340] Rudrapal M, Khairnar SJ, Khan J, Dukhyil AB, Ansari MA, Alomary MN, Alshabrmi FM, Palai S, Deb PK, Devi R. Dietary polyphenols and their role in oxidative stress-induced human diseases: insights into protective effects, antioxidant potentials and mechanism(s) of action. Front Pharmacol. 2022;13:806470. 35237163 10.3389/fphar.2022.806470 PMC8882865

[341] Zhou Y, Zheng J, Li Y, Xu DP, Li S, Chen YM, Li HB. Natural polyphenols for prevention and treatment of cancer. Nutrients. 2016;8:515. 27556486 10.3390/nu8080515 PMC4997428

[342] Zhu Z, Deng X, Xie W, Li H, Li Y, Deng Z. Pharmacological effects of bioactive agents in earthworm extract: A comprehensive review. Animal Model Exp Med. 2024;7(5):653–72. 38957072 10.1002/ame2.12465 PMC11528390

[343] Branković J, Milovanović VM, Petrović ZD, Simijonović D, Petrović VP. Pyrazolone-type compounds (part II): in vitro and in silico evaluation of antioxidant potential; structure-activity relationship. RSC Adv. 2023;13:2884–95. 36756409 10.1039/d2ra08280b PMC9846718

[344] Metwally M, Suleiman YA, Gouda M, Harmal AN, Khalil A. Synthesis, antitumor and antioxidant evaluation of some new antipyrine based azo dyes incorporating pyrazolone moiety. Int J Mod Org Chem. 2012;1:213–25.

[345] Watanabe K, Tanaka M, Yuki S, Hirai M, Yamamoto Y. How is edaravone effective against acute ischemic stroke and amyotrophic lateral sclerosis? J Clin Biochem Nutr. 2018;62:20–38. 29371752 10.3164/jcbn.17-62 PMC5773834

[346] Apaydin M, Erbas O, Taskiran D. Protection by edaravone, a radical scavenger, against manganese-induced neurotoxicity in rats. J Biochem Mol Toxicol. 2016;30:217–23. 26778341 10.1002/jbt.21780

[347] Liu J, Jiang Y, Zhang G, Lin Z, Du S. Protective effect of edaravone on blood-brain barrier by affecting NRF-2/HO-1 signaling pathway. Exp Ther Med. 2019;18:2437–42. 31555355 10.3892/etm.2019.7859 PMC6755265

[348] Duarte D, Guerreiro I, Vale N. Novel strategies for cancer combat: drug combination using repurposed drugs induces synergistic growth inhibition of MCF-7 breast and HT-29 colon cancer cells. Curr Issues Mol Biol. 2022;44:4930–49. 36286050 10.3390/cimb44100335 PMC9601176

[349] Bailly C. Potential use of edaravone to reduce specific side effects of chemo-, radio- and immuno-therapy of cancers. Int Immunopharmacol. 2019;77:105967. 31670091 10.1016/j.intimp.2019.105967

[350] Arai T, Nonogawa M, Makino K, Endo N, Mori H, Miyoshi T, Yamashita K, Sasada M, Kakuyama M, Fukuda K. The radical scavenger edaravone (3-methyl-1-phenyl-2-pyrazolin-5-one) reacts with a pterin derivative and produces a cytotoxic substance that induces intracellular reactive oxygen species generation and cell death. J Pharmacol Exp Ther. 2008;324:529–38. 18029546 10.1124/jpet.107.131391

[351] van Zandwijk N. N-acetylcysteine for lung cancer prevention. Chest. 1995;107:1437–41. 7750344 10.1378/chest.107.5.1437

[352] Pedre B, Barayeu U, Ezeriņa D, Dick TP. The mechanism of action of N-acetylcysteine (NAC): the emerging role of H2S and sulfane sulfur species. Pharmacol Ther. 2021;228:107916. 34171332 10.1016/j.pharmthera.2021.107916

[353] Miao Y, Wu Y, Jin Y, Lei M, Nan J, Wu X. Benzoquinone derivatives with antioxidant activity inhibit activated hepatic stellate cells and attenuate liver fibrosis in TAA-induced mice. Chem Biol Interact. 2020;317:108945. 31935363 10.1016/j.cbi.2020.108945

[354] Smith RA, Murphy MP. Animal and human studies with the mitochondria-targeted antioxidant MitoQ. Ann N Y Acad Sci. 2010;1201:96–103. 20649545 10.1111/j.1749-6632.2010.05627.x

[355] Capeloa T, Krzystyniak J, d’Hose D, Canas Rodriguez A, Payen VL, Zampieri LX, Van de Velde JA, Benyahia Z, Pranzini E, Vazeille T, et al. MitoQ inhibits human breast cancer cell migration, invasion and clonogenicity. Cancers (Basel). 2022;14:1516. 35326667 10.3390/cancers14061516 PMC8946220

[356] Antonenko YN, Avetisyan AV, Bakeeva LE, Chernyak BV, Chertkov VA, Domnina LV, Ivanova OY, Izyumov DS, Khailova LS, Klishin SS, et al. Mitochondria-targeted plastoquinone derivatives as tools to interrupt execution of the aging program. 1. Cationic plastoquinone derivatives: synthesis and in vitro studies. Biochemistry (Mosc). 2008;73:1273–87. 19120014 10.1134/s0006297908120018

[357] O’Donnell BV, Tew DG, Jones OT, England PJ. Studies on the inhibitory mechanism of iodonium compounds with special reference to neutrophil NADPH oxidase. Biochem J. 1993;290(Pt 1):41–9. 8439298 10.1042/bj2900041 PMC1132380

[358] Laleu B, Gaggini F, Orchard M, Fioraso-Cartier L, Cagnon L, Houngninou-Molango S, Gradia A, Duboux G, Merlot C, Heitz F, et al. First in class, potent, and orally bioavailable NADPH oxidase isoform 4 (Nox4) inhibitors for the treatment of idiopathic pulmonary fibrosis. J Med Chem. 2010;53:7715–30. 20942471 10.1021/jm100773e

[359] Morry J, Ngamcherdtrakul W, Yantasee W. Oxidative stress in cancer and fibrosis: opportunity for therapeutic intervention with antioxidant compounds, enzymes, and nanoparticles. Redox Biol. 2017;11:240–53. 28012439 10.1016/j.redox.2016.12.011 PMC5198743

[360] Konaté MM, Antony S, Doroshow JH. Inhibiting the activity of NADPH oxidase in cancer. Antioxid Redox Signal. 2020;33:435–54. 32008376 10.1089/ars.2020.8046 PMC7370979

[361] Liu Y, Lang F, Yang C. NRF2 in human neoplasm: cancer biology and potential therapeutic target. Pharmacol Ther. 2021;217:107664. 32810525 10.1016/j.pharmthera.2020.107664

[362] Robledinos-Antón N, Fernández-Ginés R, Manda G, Cuadrado A. Activators and inhibitors of NRF2: a review of their potential for clinical development. Oxid Med Cell Longev. 2019;2019:9372182. 31396308 10.1155/2019/9372182 PMC6664516

[363] Rojo AI, Medina-Campos ON, Rada P, Zúñiga-Toalá A, López-Gazcón A, Espada S, Pedraza-Chaverri J, Cuadrado A. Signaling pathways activated by the phytochemical nordihydroguaiaretic acid contribute to a Keap1-independent regulation of Nrf2 stability: role of glycogen synthase kinase-3. Free Radic Biol Med. 2012;52:473–87. 22142471 10.1016/j.freeradbiomed.2011.11.003

[364] Grossman SA, Ye X, Peereboom D, Rosenfeld MR, Mikkelsen T, Supko JG, Desideri S. Phase I study of terameprocol in patients with recurrent high-grade glioma. Neuro Oncol. 2012;14:511–7. 22323663 10.1093/neuonc/nor230 PMC3309850

[365] Ahluwalia MS, Ozair A, Rudek M, Ye X, Holdhoff M, Lieberman FS, Piotrowski AF, Nabors B, Desai A, Lesser G, et al. A multicenter, phase 1, adult brain tumor consortium trial of oral terameprocol for patients with recurrent high-grade glioma (GATOR). Cell Rep Med. 2024;5:101630. 38955178 10.1016/j.xcrm.2024.101630 PMC11293336

[366] Lastra D, Fernández-Ginés R, Manda G, Cuadrado A. Perspectives on the clinical development of NRF2-targeting drugs. Handb Exp Pharmacol. 2021;264:93–141. 32776282 10.1007/164_2020_381

[367] Crump M, Leppä S, Fayad L, Lee JJ, Di Rocco A, Ogura M, Hagberg H, Schnell F, Rifkin R, Mackensen A, et al. Randomized, double-blind, phase III trial of enzastaurin versus placebo in patients achieving remission after first-line therapy for high-risk diffuse large B-cell lymphoma. J Clin Oncol. 2016;34:2484–92. 27217449 10.1200/JCO.2015.65.7171

[368] Ball MS, Bhandari R, Torres GM, Martyanov V, ElTanbouly MA, Archambault K, Whitfield ML, Liby KT, Pioli PA. CDDO-me alters the tumor microenvironment in estrogen receptor negative breast cancer. Sci Rep. 2020;10:6560. 32300202 10.1038/s41598-020-63482-x PMC7162855

[369] Probst BL, Trevino I, McCauley L, Bumeister R, Dulubova I, Wigley WC, Ferguson DA. RTA 408, a novel synthetic triterpenoid with broad anticancer and anti-inflammatory activity. PLoS One. 2015;10:e0122942. 25897966 10.1371/journal.pone.0122942 PMC4405374

[370] Liu X, Ward K, Xavier C, Jann J, Clark AF, Pang IH, Wu H. The novel triterpenoid RTA 408 protects human retinal pigment epithelial cells against H2O2-induced cell injury via NF-E2-related factor 2 (Nrf2) activation. Redox Biol. 2016;8:98–109. 26773873 10.1016/j.redox.2015.12.005 PMC4731949

[371] Bresciani G, Manai F, Davinelli S, Tucci P, Saso L, Amadio M. Novel potential pharmacological applications of dimethyl fumarate-an overview and update. Front Pharmacol. 2023;14:1264842. 37745068 10.3389/fphar.2023.1264842 PMC10512734

[372] Li Y, Jia Y, Xu Y, Li K. DMF activates NRF2 to inhibit the pro-invasion ability of TAMs in breast cancer. Front Oncol. 2021;11:706448. 34476214 10.3389/fonc.2021.706448 PMC8406629

[373] Renken S, Nakajima T, Magalhaes I, Mattsson J, Lundqvist A, Arnér ESJ, Kiessling R, Wickström SL. Targeting of Nrf2 improves antitumoral responses by human NK cells, TI L and CAR T cells during oxidative stress. J Immunother Cancer. 2022;10:e004458. 35738800 10.1136/jitc-2021-004458 PMC9226989

[374] Shin SW, Choi C, Lee GH, Son A, Kim SH, Park HC, Batinic-Haberle I, Park W. Mechanism of the antitumor and radiosensitizing effects of a manganese porphyrin, MnHex-2-PyP. Antioxid Redox Signal. 2017;27:1067–82. 28358581 10.1089/ars.2016.6889

[375] Jang A, Petrova B, Cheong TC, Zawadzki ME, Jones JK, Culhane AJ, Shipley FB, Chiarle R, Wong ET, Kanarek N, Lehtinen MK. Choroid plexus-CSF-targeted antioxidant therapy protects the brain from toxicity of cancer chemotherapy. Neuron. 2022;110:3288-3301.e3288. 36070751 10.1016/j.neuron.2022.08.009 PMC9588748

[376] Brigelius-Flohé R, Kipp A. Glutathione peroxidases in different stages of carcinogenesis. Biochim Biophys Acta. 2009;1790:1555–68. 19289149 10.1016/j.bbagen.2009.03.006

[377] Brigelius-Flohé R, Maiorino M. Glutathione peroxidases. Biochim Biophys Acta. 1830;2013:3289–303. 10.1016/j.bbagen.2012.11.020 23201771

[378] Brigelius-Flohé R, Kipp AP. Physiological functions of GPx2 and its role in inflammation-triggered carcinogenesis. Ann N Y Acad Sci. 2012;1259:19–25. 22758632 10.1111/j.1749-6632.2012.06574.x

[379] Banning A, Kipp A, Schmitmeier S, Löwinger M, Florian S, Krehl S, Thalmann S, Thierbach R, Steinberg P, Brigelius-Flohé R. Glutathione peroxidase 2 inhibits cyclooxygenase-2-mediated migration and invasion of HT-29 adenocarcinoma cells but supports their growth as tumors in nude mice. Cancer Res. 2008;68:9746–53. 19047153 10.1158/0008-5472.CAN-08-1321

[380] Xie H, Qiang P, Wang Y, Xia F, Liu P, Li M. Discovery and mechanism studies of a novel ATG4B inhibitor ebselen by drug repurposing and its anti-colorectal cancer effects in mice. Cell Biosci. 2022;12:206. 36539845 10.1186/s13578-022-00944-x PMC9767854

[381] Wei JR, Zhang B, Zhang Y, Chen WM, Zhang XP, Zeng TT, Li Y, Zhu YH, Guan XY, Li L. QSOX1 facilitates dormant esophageal cancer stem cells to evade immune elimination via PD-L1 upregulation and CD8 T cell exclusion. Proc Natl Acad Sci U S A. 2024;121:e2407506121. 39432781 10.1073/pnas.2407506121 PMC11536095

[382] Hanavan PD, Borges CR, Katchman BA, Faigel DO, Ho TH, Ma CT, Sergienko EA, Meurice N, Petit JL, Lake DF. Ebselen inhibits QSOX1 enzymatic activity and suppresses invasion of pancreatic and renal cancer cell lines. Oncotarget. 2015;6:18418–28. 26158899 10.18632/oncotarget.4099 PMC4621900

[383] Zhong L, Xia Y, He T, Wenjie S, Jinxia A, Lijun Y, Hui G. Polymeric photothermal nanoplatform with the inhibition of aquaporin 3 for anti-metastasis therapy of breast cancer. Acta Biomater. 2022;153:505–17. 36115652 10.1016/j.actbio.2022.09.026

[384] Charlestin V, Tan E, Arias-Matus CE, Wu J, Miranda-Vergara MC, Lee M, Wang M, Nannapaneni DT, Tennakoon P, Blagg BSJ, et al. Evaluation of the mammalian aquaporin inhibitors Auphen and Z433927330 in treating breast cancer. Cancers (Basel). 2024;16:2714. 39123442 10.3390/cancers16152714 PMC11311482

[385] Sonntag Y, Gena P, Maggio A, Singh T, Artner I, Oklinski MK, Johanson U, Kjellbom P, Nieland JD, Nielsen S, et al. Identification and characterization of potent and selective aquaporin-3 and aquaporin-7 inhibitors. J Biol Chem. 2019;294:7377–87. 30862673 10.1074/jbc.RA118.006083 PMC6509502

[386] De Ieso ML, Yool AJ. Mechanisms of aquaporin-facilitated cancer invasion and metastasis. Front Chem. 2018;6:135. 29922644 10.3389/fchem.2018.00135 PMC5996923

[387] De Ieso ML, Pei JV, Nourmohammadi S, Smith E, Chow PH, Kourghi M, Hardingham JE, Yool AJ. Combined pharmacological administration of AQP1 ion channel blocker AqB011 and water channel blocker Bacopaside II amplifies inhibition of colon cancer cell migration. Sci Rep. 2019;9:12635. 31477744 10.1038/s41598-019-49045-9 PMC6718670

[388] Stankovic JSK, Selakovic D, Mihailovic V, Rosic G. Antioxidant supplementation in the treatment of neurotoxicity induced by platinum-based chemotherapeutics-a review. Int J Mol Sci. 2020;21:7753. 33092125 10.3390/ijms21207753 PMC7589133

[389] Xia Y, Gui H, Li X, Wu Y, Liu J, Liu J. X-ray responsive antioxidant drug-free hydrogel for treatment of radiation skin injury. ACS Appl Mater Interfaces. 2025;17:5671–83. 39825803 10.1021/acsami.4c16810

[390] Olufunmilayo EO, Gerke-Duncan MB, Holsinger RMD. Oxidative stress and antioxidants in neurodegenerative disorders. Antioxidants (Basel). 2023;12:517. 36830075 10.3390/antiox12020517 PMC9952099

[391] Barnes PJ. Oxidative stress in chronic obstructive pulmonary disease. Antioxidants (Basel). 2022;11:965. 35624831 10.3390/antiox11050965 PMC9138026

[392] Halliwell B. Understanding mechanisms of antioxidant action in health and disease. Nat Rev Mol Cell Biol. 2024;25:13–33. 37714962 10.1038/s41580-023-00645-4

[393] Florido J, Martinez-Ruiz L, Rodriguez-Santana C, López-Rodríguez A, Hidalgo-Gutiérrez A, Cottet-Rousselle C, Lamarche F, Schlattner U, Guerra-Librero A, Aranda-Martínez P, et al. Melatonin drives apoptosis in head and neck cancer by increasing mitochondrial ROS generated via reverse electron transport. J Pineal Res. 2022;73:e12824. 35986493 10.1111/jpi.12824 PMC9541246

[394] Jain S, Hu C, Kluza J, Ke W, Tian G, Giurgiu M, Bleilevens A, Campos AR, Charbono A, Stickeler E, et al. Metabolic targeting of cancer by a ubiquinone uncompetitive inhibitor of mitochondrial complex I. Cell Chem Biol. 2022;29:436-450.e415. 34852219 10.1016/j.chembiol.2021.11.002

[395] Wheaton WW, Weinberg SE, Hamanaka RB, Soberanes S, Sullivan LB, Anso E, Glasauer A, Dufour E, Mutlu GM, Budigner GS, Chandel NS. Metformin inhibits mitochondrial complex I of cancer cells to reduce t umorigenesis. Elife. 2014;3:e02242. 24843020 10.7554/eLife.02242 PMC4017650

[396] Altinoz MA, Ozpinar A. Oxamate targeting aggressive cancers with special emphasis to brain tumors. Biomed Pharmacother. 2022;147:112686. 35124385 10.1016/j.biopha.2022.112686

[397] Miskimins WK, Ahn HJ, Kim JY, Ryu S, Jung YS, Choi JY. Synergistic anti-cancer effect of phenformin and oxamate. PLoS One. 2014;9:e85576. 24465604 10.1371/journal.pone.0085576 PMC3897486

[398] Shi J, Ju R, Gao H, Huang Y, Guo L, Zhang D. Targeting glutamine utilization to block metabolic adaptation of tumor cells under the stress of carboxyamidotriazole-induced nutrients unavailability. Acta Pharm Sin B. 2022;12:759–73. 35256945 10.1016/j.apsb.2021.07.008 PMC8897199

[399] Marullo R, Werner E, Degtyareva N, Moore B, Altavilla G, Ramalingam SS, Doetsch PW. Cisplatin induces a mitochondrial-ROS response that contributes to cytotoxicity depending on mitochondrial redox status and bioenergetic functions. PLoS One. 2013;8:e81162. 24260552 10.1371/journal.pone.0081162 PMC3834214

[400] Kleih M, Böpple K, Dong M, Gaißler A, Heine S, Olayioye MA, Aulitzky WE, Essmann F. Direct impact of cisplatin on mitochondria induces ROS production that dictates cell fate of ovarian cancer cells. Cell Death Dis. 2019;10:851. 31699970 10.1038/s41419-019-2081-4 PMC6838053

[401] Iwama K, Nakajo S, Aiuchi T, Nakaya K. Apoptosis induced by arsenic trioxide in leukemia U937 cells is dependent on activation of p38, inactivation of ERK and the Ca2+-dependent production of superoxide. Int J Cancer. 2001;92:518–26. 11304686 10.1002/ijc.1220

[402] Pelicano H, Feng L, Zhou Y, Carew JS, Hileman EO, Plunkett W, Keating MJ, Huang P. Inhibition of mitochondrial respiration: a novel strategy to enhance drug-induced apoptosis in human leukemia cells by a reactive oxygen species-mediated mechanism. J Biol Chem. 2003;278:37832–9. 12853461 10.1074/jbc.M301546200

[403] Lu J, Chew EH, Holmgren A. Targeting thioredoxin reductase is a basis for cancer therapy by arsenic trioxide. Proc Natl Acad Sci U S A. 2007;104:12288–93. 17640917 10.1073/pnas.0701549104 PMC1940330

[404] Sinha BK, Katki AG, Batist G, Cowan KH, Myers CE. Differential formation of hydroxyl radicals by adriamycin in sensitive and resistant MCF-7 human breast tumor cells: implications for the mechanism of action. Biochemistry. 1987;26:3776–81. 2820475 10.1021/bi00387a006

[405] Tsang WP, Chau SPY, Kong SK, Fung KP, Kwok TT. Reactive oxygen species mediate doxorubicin induced p53-independent apoptosis. Life Sci. 2003;73:2047–58. 12899928 10.1016/s0024-3205(03)00566-6

[406] Mizutani H, Tada-Oikawa S, Hiraku Y, Kojima M, Kawanishi S. Mechanism of apoptosis induced by doxorubicin through the generation of hydrogen peroxide. Life Sci. 2005;76:1439–53. 15680309 10.1016/j.lfs.2004.05.040

[407] Tu Y, Zhang W, Fan G, Zou C, Zhang J, Wu N, Ding J, Zou WQ, Xiao H, Tan S. Paclitaxel-loaded ROS-responsive nanoparticles for head and neck cancer therapy. Drug Deliv. 2023;30:2189106. 36916054 10.1080/10717544.2023.2189106 PMC10026753

[408] Cao L, Tian H, Fang M, Xu Z, Tang D, Chen J, Yin J, Xiao H, Shang K, Han H, Li X. Activating cGAS-STING pathway with ROS-responsive nanoparticles delivering a hybrid prodrug for enhanced chemo-immunotherapy. Biomaterials. 2022;290:121856. 36306685 10.1016/j.biomaterials.2022.121856

[409] Xu Q, Zhan G, Zhang Z, Yong T, Yang X, Gan L. Manganese porphyrin-based metal-organic framework for synergistic sonodynamic therapy and ferroptosis in hypoxic tumors. Theranostics. 2021;11:1937–52. 33408790 10.7150/thno.45511 PMC7778611

[410] Chen Y, Li Y, Huang L, Du Y, Gan F, Li Y, Yao Y. Antioxidative stress: inhibiting reactive oxygen species production as a cause of radioresistance and chemoresistance. Oxid Med Cell Longev. 2021;2021:6620306. 33628367 10.1155/2021/6620306 PMC7884184

[411] Kwiatkowski S, Knap B, Przystupski D, Saczko J, Kędzierska E, Knap-Czop K, Kotlińska J, Michel O, Kotowski K, Kulbacka J. Photodynamic therapy – mechanisms, photosensitizers and combinations. Biomed Pharmacother. 2018;106:1098–107. 30119176 10.1016/j.biopha.2018.07.049

[412] Chong LM, Tng DJH, Tan LLY, Chua MLK, Zhang Y. Recent advances in radiation therapy and photodynamic therapy. Appl Phys Rev. 2021;8:041322.

[413] Pan X, Wang H, Wang S, Sun X, Wang L, Wang W, Shen H, Liu H. Sonodynamic therapy (SDT): a novel strategy for cancer nanotheranostics. Sci China Life Sci. 2018;61:415–26. 29666990 10.1007/s11427-017-9262-x

[414] He Z, Du J, Miao Y, Li Y. Recent developments of inorganic nanosensitizers for sonodynamic therapy. Adv Healthc Mater. 2023;12:e2300234. 37070721 10.1002/adhm.202300234

[415] Lin H, Chen Y, Shi J. Nanoparticle-triggered in situ catalytic chemical reactions for tumour -specific therapy. Chem Soc Rev. 2018;47:1938–58. 29417106 10.1039/c7cs00471k

[416] Dixon SJ, Patel DN, Welsch M, Skouta R, Lee ED, Hayano M, Thomas AG, Gleason CE, Tatonetti NP, Slusher BS, Stockwell BR. Pharmacological inhibition of cystine-glutamate exchange induces endoplasmic reticulum stress and ferroptosis. Elife. 2014;3:e02523. 24844246 10.7554/eLife.02523 PMC4054777

[417] Dolma S, Lessnick SL, Hahn WC, Stockwell BR. Identification of genotype-selective antitumor agents using synthetic lethal chemical screening in engineered human tumor cells. Cancer Cell. 2003;3:285–96. 12676586 10.1016/s1535-6108(03)00050-3

[418] Yagoda N, von Rechenberg M, Zaganjor E, Bauer AJ, Yang WS, Fridman DJ, Wolpaw AJ, Smukste I, Peltier JM, Boniface JJ, et al. RAS-RAF-MEK-dependent oxidative cell death involving voltage-dependent anion channels. Nature. 2007;447:864–8. 17568748 10.1038/nature05859 PMC3047570

[419] Zhang Y, Tan H, Daniels JD, Zandkarimi F, Liu H, Brown LM, Uchida K, O’Connor OA, Stockwell BR. Imidazole ketone erastin induces ferroptosis and slows tumor growth in a mouse lymphoma model. Cell Chem Biol. 2019;26:623-633.e629. 30799221 10.1016/j.chembiol.2019.01.008 PMC6525071

[420] Abdelgalil AA, Alkahtani HM, Al-Jenoobi FI. Sorafenib. Profiles Drug Subst Excip Relat Methodol. 2019;44:239–66. 31029219 10.1016/bs.podrm.2018.11.003

[421] Cramer SL, Saha A, Liu J, Tadi S, Tiziani S, Yan W, Triplett K, Lamb C, Alters SE, Rowlinson S, et al. Systemic depletion of L-cyst(e)ine with cyst(e)inase increases reactive oxygen species and suppresses tumor growth. Nat Med. 2017;23:120–7. 27869804 10.1038/nm.4232 PMC5218918

[422] Nishizawa S, Araki H, Ishikawa Y, Kitazawa S, Hata A, Soga T, Hara T. Low tumor glutathione level as a sensitivity marker for glutamate-cysteine ligase inhibitors. Oncol Lett. 2018;15:8735–43. 29928324 10.3892/ol.2018.8447 PMC6004701

[423] Gupta P, Srivastava SK. Antitumor activity of phenethyl isothiocyanate in HER2-positive breast cancer models. BMC Med. 2012;10:80. 22824293 10.1186/1741-7015-10-80 PMC3412708

[424] Chen G, Chen Z, Hu Y, Huang P. Inhibition of mitochondrial respiration and rapid depletion of mitochondrial glutathione by β-phenethyl isothiocyanate: mechanisms for anti-leukemia activity. Antioxid Redox Signal. 2011;15:2911–21. 21827296 10.1089/ars.2011.4170 PMC3201634

[425] Wu WJ, Zhang Y, Zeng ZL, Li XB, Hu KS, Luo HY, Yang J, Huang P, Xu RH. β-phenylethyl isothiocyanate reverses platinum resistance by a GSH-dependent mechanism in cancer cells with epithelial-mesenchymal transition phenotype. Biochem Pharmacol. 2013;85:486–96. 23219523 10.1016/j.bcp.2012.11.017

[426] Wu X, Zhu Y, Yan H, Liu B, Li Y, Zhou Q, Xu K. Isothiocyanates induce oxidative stress and suppress the metastasis potential of human non-small cell lung cancer cells. BMC Cancer. 2010;10:269. 20534110 10.1186/1471-2407-10-269 PMC2891640

[427] Xiang Y, Ye W, Huang C, Yu D, Chen H, Deng T, Zhang F, Lou B, Zhang J, Shi K, et al. Brusatol enhances the chemotherapy efficacy of gemcitabine in pancreatic cancer via the Nrf2 signalling pathway. Oxid Med Cell Longev. 2018;2018:2360427. 29849873 10.1155/2018/2360427 PMC5932458

[428] Tsuchida K, Tsujita T, Hayashi M, Ojima A, Keleku-Lukwete N, Katsuoka F, Otsuki A, Kikuchi H, Oshima Y, Suzuki M, Yamamoto M. Halofuginone enhances the chemo-sensitivity of cancer cells by suppressing NRF2 accumulation. Free Radic Biol Med. 2017;103:236–47. 28039084 10.1016/j.freeradbiomed.2016.12.041

[429] Jeong EJ, Choi JJ, Lee SY, Kim YS. The effects of ML385 on head and neck squamous cell carcinoma: implications for NRF2 inhibition as a therapeutic strategy. Int J Mol Sci. 2024;25:7011. 39000120 10.3390/ijms25137011 PMC11241175

[430] Yang H, Zheng W, Lin H, Huang H. ML385 Suppresses Hypoxia-Induced Drug Resistance and Cancer Stemness of Breast Cancer Cells by Blocking the Nrf2/HO-1 Pathway. Pharm Chem J. 2025;58:1506–13.

[431] Tan Y, Bi L, Zhang P, Wang F, Lin F, Ni W, Wu J, Jiang L. Thioredoxin-1 inhibitor PX-12 induces human acute myeloid leukemia cell apoptosis and enhances the sensitivity of cells to arsenic trioxide. Int J Clin Exp Pathol. 2014;7:4765–73. 25197347 PMC4152037

[432] Wu GN, Ford JM, Alger JR. MRI measurement of the uptake and retention of motexafin gadolinium in glioblastoma multiforme and uninvolved normal human brain. J Neurooncol. 2006;77:95–103. 16547607 10.1007/s11060-005-9101-1

[433] Ford JM, Seiferheld W, Alger JR, Wu G, Endicott TJ, Mehta M, Curran W, Phan SC. Results of the phase I dose-escalating study of motexafin gadolinium with standard radiotherapy in patients with glioblastoma multiforme. Int J Radiat Oncol Biol Phys. 2007;69:831–8. 17560737 10.1016/j.ijrobp.2007.04.017

[434] Zhong M, Chen L, Tao Y, Zhao J, Chang B, Zhang F, Tu J, Cai W, Zhang B. Synthesis and evaluation of Piperine analogs as thioredoxin reductase inhibitors to cause oxidative stress-induced cancer cell apoptosis. Bioorgan Chem. 2023;138:106589. 10.1016/j.bioorg.2023.106589 37320912

[435] Amato RJ, Jac J, Hernandez-McClain J. Motexafin gadolinium for the treatment of metastatic renal cell carcinoma: phase II study results. Clin Genitourin Cancer. 2008;6:73–8. 18824428 10.3816/CGC.2008.n.011

[436] Evens AM, Lecane P, Magda D, Prachand S, Singhal S, Nelson J, Miller RA, Gartenhaus RB, Gordon LI. Motexafin gadolinium generates reactive oxygen species and induces apoptosis in sensitive and highly resistant multiple myeloma cells. Blood. 2005;105:1265–73. 15388578 10.1182/blood-2004-03-0964

[437] Chen J, Ramos J, Sirisawad M, Miller R, Naumovski L. Motexafin gadolinium induces mitochondrially-mediated caspase-dependent apoptosis. Apoptosis. 2005;10:1131–42. 16151646 10.1007/s10495-005-0887-2

[438] An SC, Jun HH, Kim KM, Kim I, Choi S, Yeo H, Lee S, An HJ. Auranofin as a novel anticancer drug for anaplastic thyroid cancer. Pharmaceuticals (Basel). 2024;17:1394. 39459033 10.3390/ph17101394 PMC11510098

[439] Abdalbari FH, Martinez-Jaramillo E, Forgie BN, Tran E, Zorychta E, Goyeneche AA, Sabri S, Telleria CM. Auranofin induces lethality driven by reactive oxygen species in high-grade serous ovarian cancer cells. Cancers (Basel). 2023;15:5136. 37958311 10.3390/cancers15215136 PMC10650616

[440] Freire Boullosa L, Van Loenhout J, Hermans C, Lau HW, Merlin C, Marcq E, Takhsha FS, Martinet W, De Meyer GRY, Lardon F, et al. Optimization of the solvent and in vivo administration route of auranofin in a syngeneic non-small cell lung cancer and glioblastoma mouse model. Pharmaceutics. 2022;14:2761. 36559255 10.3390/pharmaceutics14122761 PMC9783082

[441] Wang R, Zhong L, Wang T, Sun T, Yang J, Liu X, Wu Y, Guo Q, Gao Y, Zhao K. Inducing ubiquitination and degradation of TrxR1 protein by LW-216 promotes apoptosis in non-small cell lung cancer via triggering ROS production. Neoplasia. 2024;53:101004. 38733769 10.1016/j.neo.2024.101004 PMC11104261

[442] Onodera T, Momose I, Adachi H, Yamazaki Y, Sawa R, Ohba SI, Kawada M. Human pancreatic cancer cells under nutrient deprivation are vulnerable to redox system inhibition. J Biol Chem. 2020;295:16678–90. 32978257 10.1074/jbc.RA120.013893 PMC7864064

[443] Fiskus W, Saba N, Shen M, Ghias M, Liu J, Gupta SD, Chauhan L, Rao R, Gunewardena S, Schorno K, et al. Auranofin induces lethal oxidative and endoplasmic reticulum stress and exerts potent preclinical activity against chronic lymphocytic leukemia. Cancer Res. 2014;74:2520–32. 24599128 10.1158/0008-5472.CAN-13-2033 PMC4172421

[444] Wu W, Yang Z, Xiao X, An T, Li B, Ouyang J, Li H, Wang C, Zhang Y, Zhang H, et al. A thioredoxin reductase inhibitor ethaselen induces growth inhibition and apoptosis in gastric cancer. J Cancer. 2020;11:3013–9. 32226516 10.7150/jca.40744 PMC7086267

[445] Ye SF, Li J, Ji SM, Zeng HH, Lu W. Dose-biomarker-response modeling of the anticancer effect of ethaselen in a human non-small cell lung cancer xenograft mouse model. Acta Pharmacol Sin. 2017;38:223–32. 27917873 10.1038/aps.2016.114 PMC5309752

[446] Wondrak GT. NQO1-activated phenothiazinium redox cyclers for the targeted bioreductive induction of cancer cell apoptosis. Free Radic Biol Med. 2007;43:178–90. 17603928 10.1016/j.freeradbiomed.2007.03.035 PMC2705808

[447] Zhang Q, Ma Y, Cheng YF, Li WJ, Zhang Z, Chen S-Y. Involvement of reactive oxygen species in 2-methoxyestradiol-induced apoptosis in human neuroblastoma cells. Cancer Lett. 2011;313:201–10. 21978530 10.1016/j.canlet.2011.09.005 PMC3224534

[448] Lambert AJ, Brand MD. Inhibitors of the quinone-binding site allow rapid superoxide production from mitochondrial NADH: ubiquinone oxidoreductase (complex I). J Biol Chem. 2004;279:39414–20. 15262965 10.1074/jbc.M406576200

[449] Bridges HR, Jones AJ, Pollak MN, Hirst J. Effects of metformin and other biguanides on oxidative phosphorylation in mitochondria. Biochem J. 2014;462:475–87. 25017630 10.1042/BJ20140620 PMC4148174

[450] Feng J, Wang X, Ye X, Ares I, Lopez-Torres B, Martínez M, Martínez-Larrañaga MR, Wang X, Anadón A, Martínez MA. Mitochondria as an important target of metformin: the mechanism of action, toxic and side effects, and new therapeutic applications. Pharmacol Res. 2022;177:106114. 35124206 10.1016/j.phrs.2022.106114

[451] Bragado P, Armesilla A, Silva A, Porras A. Apoptosis by cisplatin requires p53 mediated p38alpha MAPK activation through ROS generation. Apoptosis. 2007;12:1733–42. 17505786 10.1007/s10495-007-0082-8

[452] Jung K, Reszka R. Mitochondria as subcellular targets for clinically useful anthracyclines. Adv Drug Deliv Rev. 2001;49:87–105. 11377805 10.1016/s0169-409x(01)00128-4

[453] Chatterjee S, Hofer T, Costa A, Lu D, Batkai S, Gupta SK, Bolesani E, Zweigerdt R, Megias D, Streckfuss-Bömeke K, et al. Telomerase therapy attenuates cardiotoxic effects of doxorubicin. Mol Ther. 2021;29:1395–410. 33388418 10.1016/j.ymthe.2020.12.035 PMC8058493

[454] Deng S, Kruger A, Kleschyov AL, Kalinowski L, Daiber A, Wojnowski L. Gp91phox-containing NAD(P)H oxidase increases superoxide formation by doxorubicin and NADPH. Free Radic Biol Med. 2007;42:466–73. 17275678 10.1016/j.freeradbiomed.2006.11.013

[455] Kciuk M, Gielecińska A, Mujwar S, Kołat D, Kałuzińska-Kołat Ż, Celik I, Kontek R. Doxorubicin-an agent with multiple mechanisms of anticancer activity. Cells. 2023;12:659. 36831326 10.3390/cells12040659 PMC9954613

[456] Azzam EI, Jay-Gerin JP, Pain D. Ionizing radiation-induced metabolic oxidative stress and prolonged cell injury. Cancer Lett. 2012;327:48–60. 22182453 10.1016/j.canlet.2011.12.012 PMC3980444

[457] Yang B, Chen Y, Shi J. Reactive Oxygen Species (ROS)-based nanomedicine. Chem Rev. 2019;119:4881–985. 30973011 10.1021/acs.chemrev.8b00626

[458] Wang H, Mu X, He H, Zhang XD. Cancer radiosensitizers. Trends Pharmacol Sci. 2018;39:24–48. 29224916 10.1016/j.tips.2017.11.003

[459] Agostinis P, Berg K, Cengel KA, Foster TH, Girotti AW, Gollnick SO, Hahn SM, Hamblin MR, Juzeniene A, Kessel D, et al. Photodynamic therapy of cancer: an update. CA Cancer J Clin. 2011;61:250–81. 21617154 10.3322/caac.20114 PMC3209659

[460] Guo QL, Dai XL, Yin MY, Cheng HW, Qian HS, Wang H, Zhu DM, Wang X-W. Nanosensitizers for sonodynamic therapy for glioblastoma multiforme: current progress and future perspectives. Mil Med Res. 2022;9:26. 35676737 10.1186/s40779-022-00386-z PMC9178901

[461] Pan MM, Li P, Yu YP, et al. Bimetallic Ions Functionalized Metal-Organic-Framework Nanozyme for Tumor Microenvironment Regulating and Enhanced Photodynamic Therapy for Hypoxic Tumor. Adv Healthc Mater. 2023;12(26):e2300821. 37199497 10.1002/adhm.202300821

[462] Lan M, Zhao S, Liu W, Lee CS, Zhang W, Wang P. Photosensitizers for photodynamic therapy. Adv Healthc Mater. 2019;8:1900132. 10.1002/adhm.201900132 31067008

[463] Lan G, Ni K, Veroneau SS, Song Y, Lin W. Nanoscale metal-organic layers for radiotherapy-radiodynamic therapy. J Am Chem Soc. 2018;140:16971–5. 30485084 10.1021/jacs.8b11593

[464] Zhu S, Yan F, Yang L, Li B, Xue R, Yu W, Wang Y, Huang L, Wang L, Han R, Jiang Y. Low-dose X-ray radiodynamic therapy solely based on gold nanoclusters for efficient treatment of deep hypoxic solid tumors combined with enhanced antitumor immune response. Theranostics. 2023;13:1042–58. 36793856 10.7150/thno.78649 PMC9925321

[465] Jiang F, Lee C, Zhang W, Jiang W, Cao Z, Chong HB, Yang W, Zhan S, Li J, Teng Y, et al. Radiodynamic therapy with CsI(na)@MgO nanoparticles and 5-aminolevulinic acid. J Nanobiotechnol. 2022;20:330. 10.1186/s12951-022-01537-z PMC9288050 35842630

[466] Zhong X, Wang X, Zhan G, Tang YA, Yao Y, Dong Z, Hou L, Zhao H, Zeng S, Hu J, et al. NaCeF4:Gd, Tb scintillator as an X-ray responsive photosensitizer for multimodal imaging-guided synchronous radio/radiodynamic therapy. Nano Lett. 2019;19:8234–44. 31576757 10.1021/acs.nanolett.9b03682

[467] Liu J, Hu F, Wu M, Tian L, Gong F, Zhong X, Chen M, Liu Z, Liu B. Bioorthogonal coordination polymer nanoparticles with aggregation-induced emission for deep tumor-penetrating radio- and radiodynamic therapy. Adv Mater. 2021;33:e2007888. 33491820 10.1002/adma.202007888

[468] Loke YL, Beishenaliev A, Wang P-W, Lin CY, Chang CY, Foo YY, Faruqu FN, Leo BF, Misran M, Chung LY, et al. ROS-generating alginate-coated gold nanorods as biocompatible nanosonosensitisers for effective sonodynamic therapy of cancer. Ultrason Sonochem. 2023;96:106437. 37187119 10.1016/j.ultsonch.2023.106437 PMC10197111

[469] Qu X, Yin F, Pei M, Chen Q, Zhang Y, Lu S, Zhang X, Liu Z, Li X, Chen H, et al. Modulation of intratumoral *Fusobacterium nucleatum* to enhance sonodynamic therapy for colorectal cancer with reduced phototoxic skin injury. ACS Nano. 2023;17:11466–80. 37201179 10.1021/acsnano.3c01308 PMC10311605

[470] Nowak KM, Schwartz MR, Breza VR, Price RJ. Sonodynamic therapy: rapid progress and new opportunities for non-invasive tumor cell killing with sound. Cancer Lett. 2022;532:215592. 35151824 10.1016/j.canlet.2022.215592 PMC8918024

[471] Wang P, Wang X, Ma L, Sahi S, Li L, Wang X, Wang Q, Chen Y, Chen W, Liu Q. Nanosonosensitization by Using Copper-Cysteamine Nanoparticles Augmented Sonodynamic Cancer Treatment. Part Part Syst Charact. 2018;35:1700378.

[472] Tang Z, Liu Y, He M, Bu W. Chemodynamic therapy: tumour microenvironment-mediated Fenton and Fenton-like reactions. Angew Chem Int Ed Engl. 2019;58:946–56. 30048028 10.1002/anie.201805664

[473] Jia C, Guo Y, Wu FG. Chemodynamic therapy via Fenton and Fenton-like nanomaterials: strategies and recent advances. Small. 2022;18:e2103868. 34729913 10.1002/smll.202103868

[474] Valente A, Podolski-Renić A, Poetsch I, Filipović N, López Ó, Turel I, Heffeter P. Metal- and metalloid-based compounds to target and reverse cancer multidrug resistance. Drug Resist Updat. 2021;58:100778. 34403910 10.1016/j.drup.2021.100778

[475] Lu J, Holmgren A. The thioredoxin antioxidant system. Free Radic Biol Med. 2014;66:75–87. 23899494 10.1016/j.freeradbiomed.2013.07.036

[476] Lu SC. Regulation of glutathione synthesis. Mol Aspects Med. 2009;30:42–59. 18601945 10.1016/j.mam.2008.05.005 PMC2704241

[477] Parker JL, Deme JC, Kolokouris D, Kuteyi G, Biggin PC, Lea SM, Newstead S. Molecular basis for redox control by the human cystine/glutamate antiporter system xc. Nat Commun. 2021;12:7147. 34880232 10.1038/s41467-021-27414-1 PMC8654953

[478] Liu J, Xia X, Huang P. xCT: a critical molecule that links cancer metabolism to redox signaling. Mol Ther. 2020;28:2358–66. 32931751 10.1016/j.ymthe.2020.08.021 PMC7647670

[479] Li FJ, Long HZ, Zhou ZW, Luo HY, Xu SG, Gao LC. System X_c_^-^/GSH/GPX4 *axis*: an important antioxidant system for the ferroptosis in drug-resistant solid tumor therapy. Front Pharmacol. 2022;13:910292. 36105219 10.3389/fphar.2022.910292 PMC9465090

[480] Zhao Y, Li Y, Zhang R, Wang F, Wang T, Jiao Y. The role of erastin in ferroptosis and its prospects in cancer therapy. Onco Targets Ther. 2020;13:5429–41. 32606760 10.2147/OTT.S254995 PMC7295539

[481] Yu M, Gai C, Li Z, Ding D, Zheng J, Zhang W, Lv S, Li W. Targeted exosome-encapsulated erastin induced ferroptosis in triple negative breast cancer cells. Cancer Sci. 2019;110:3173–82. 31464035 10.1111/cas.14181 PMC6778638

[482] Larraufie MH, Yang WS, Jiang E, Thomas AG, Slusher BS, Stockwell BR. Incorporation of metabolically stable ketones into a small molecule probe to increase potency and water solubility. Bioorg Med Chem Lett. 2015;25:4787–92. 26231156 10.1016/j.bmcl.2015.07.018 PMC4653046

[483] Cao X, Li M, Liu Q, Zhao J, Lu X, Wang J. Inorganic Sonosensitizers for Sonodynamic Therapy in Cancer Treatment Abstract. Small. 2023;19(42). 10.1002/smll.v19.42. 10.1002/smll.202303195. 10.1002/smll.202303195 37323087

[484] Rao Z, Xia Y, Jia Q, Zhu Y, Wang L, Liu G, Liu X, Yang P, Ning P, Zhang R, et al. Iron-based metal-organic framework co-loaded with buthionine sulfoximine and oxaliplatin for enhanced cancer chemo-ferrotherapy via sustainable glutathione elimination. J Nanobiotechnol. 2023;21:265. 10.1186/s12951-023-01998-w PMC10416514 37563614

[485] Gao Y, Li Y, Cao H, Jia H, Wang D, Ren C, Wang Z, Yang C, Liu J. Hypertoxic self-assembled peptide with dual functions of glutathione depletion and biosynthesis inhibition for selective tumor ferroptosis and pyroptosis. J Nanobiotechnol. 2022;20:390. 10.1186/s12951-022-01604-5 PMC9429723 36045424

[486] Zhang Y. The molecular basis that unifies the metabolism, cellular uptake and chemopreventive activities of dietary isothiocyanates. Carcinogenesis. 2012;33:2–9. 22080571 10.1093/carcin/bgr255 PMC3276327

[487] Upadhyaya B, Liu Y, Dey M. Phenethyl isothiocyanate exposure promotes oxidative stress and suppresses Sp1 transcription factor in cancer stem cells. Int J Mol Sci. 2019;20:1027. 30818757 10.3390/ijms20051027 PMC6429440

[488] Gupta P, Wright SE, Kim SH, Srivastava SK. Phenethyl isothiocyanate: a comprehensive review of anti-cancer mechanisms. Biochim Biophys Acta. 2014;1846:405–24. 25152445 10.1016/j.bbcan.2014.08.003 PMC4260992

[489] Slawik C, Rickmeyer C, Brehm M, Böhme A, Schüürmann G. Glutathione adduct patterns of Michael-acceptor carbonyls. Environ Sci Technol. 2017;51:4018–26. 28225253 10.1021/acs.est.6b04981

[490] Liu Y, Zhou Z, Liu Y, Li Y, Huang X, Qian C, Sun M. H2O2-activated oxidative stress amplifier capable of GSH scavenging for enhancing tumor photodynamic therapy. Biomater Sci. 2019;7:5359–68. 31621699 10.1039/c9bm01354g

[491] Wang H, Chen T, Ren H, Liu W, Nan F, Ge J, Wang P. Metal-organic Frameworks@Au nanoreactor as an oxidative stress amplifier for enhanced tumor photodynamic therapy through the alleviation of hypoxemia and the depletion of glutathione. ACS Appl Biomater. 2023. 10.1021/acsabm.1022c01090. 10.1021/acsabm.2c01090 36912885

[492] Wang R, Liang L, Matsumoto M, Iwata K, Umemura A, He F. Reactive oxygen species and NRF2 signaling, friends or foes in cancer? Biomolecules. 2023;13:353. 36830722 10.3390/biom13020353 PMC9953152

[493] Menegon S, Columbano A, Giordano S. The dual roles of NRF2 in cancer. Trends Mol Med. 2016;22:578–93. 27263465 10.1016/j.molmed.2016.05.002

[494] Zhang J, Xu HX, Zhu JQ, Dou YX, Xian YF, Lin ZX. Natural Nrf2 inhibitors: a review of their potential for cancer treatment. Int J Biol Sci. 2023;19:3029–41. 37416770 10.7150/ijbs.82401 PMC10321279

[495] Panieri E, Saso L. Inhibition of the NRF2/KEAP1 axis: a promising therapeutic strategy to alter redox balance of cancer cells. Antioxid Redox Signal. 2021;34:1428–83. 33403898 10.1089/ars.2020.8146

[496] Xi W, Zhao C, Wu Z, Ye T, Zhao R, Jiang X, Ling S. Brusatol’s anticancer activity and its molecular mechanism: a research update. J Pharm Pharmacol. 2024;76:753–62. 38394388 10.1093/jpp/rgae017

[497] Gjorgieva Ackova D, Maksimova V, Smilkov K, Buttari B, Arese M, Saso L. Alkaloids as natural NRF2 inhibitors: chemoprevention and cytotoxic action in cancer. Pharmaceuticals (Basel). 2023;16:850. 37375797 10.3390/ph16060850 PMC10300961

[498] Krajka-Kuźniak V, Baer-Dubowska W. Modulation of Nrf2 and NF-κB signaling pathways by naturally occurring compounds in relation to cancer prevention and therapy. Are combinations better than single compounds? Int J Mol Sci. 2021;22:8223. 34360990 10.3390/ijms22158223 PMC8348704

[499] Singh A, Venkannagari S, Oh KH, Zhang YQ, Rohde JM, Liu L, Nimmagadda S, Sudini K, Brimacombe KR, Gajghate S, et al. Small molecule inhibitor of NRF2 selectively intervenes therapeutic resistance in KEAP1-deficient NSCLC tumors. ACS Chem Biol. 2016;11:3214–25. 27552339 10.1021/acschembio.6b00651 PMC5367156

[500] Yan L, Hu H, Feng L, Li Z, Zheng C, Zhang J, Yin X, Li B. ML385 promotes ferroptosis and radiotherapy sensitivity by inhibiting the NRF2-SLC7A11 pathway in esophageal squamous cell carcinoma. Med Oncol. 2024;41:309. 39511054 10.1007/s12032-024-02483-6 PMC11543766

[501] Juszczak M, Tokarz P, Woźniak K. Potential of NRF2 inhibitors-retinoic acid, K67, and ML-385-in overcoming doxorubicin resistance in promyelocytic leukemia cells. Int J Mol Sci. 2024;25:10257. 39408587 10.3390/ijms251910257 PMC11476837

[502] Baker AF, Adab KN, Raghunand N, Chow H, Stratton SP, Squire SW, Boice M, Pestano LA, Kirkpatrick DL, Dragovich T. A phase IB trial of 24-hour intravenous PX-12, a thioredoxin-1 inhibitor, in patients with advanced gastrointestinal cancers. Invest New Drugs. 2013;31:631–41. 22711542 10.1007/s10637-012-9846-2 PMC3988981

[503] Kirkpatrick DL, Kuperus M, Dowdeswell M, Potier N, Donald LJ, Kunkel M, Berggren M, Angulo M, Powis G. Mechanisms of inhibition of the thioredoxin growth factor system by antitumor 2-imidazolyl disulfides. Biochem Pharmacol. 1998;55:987–94. 9605422 10.1016/s0006-2952(97)00597-2

[504] Akhlaq R, Khan T, Ahmed T, Musharraf SG, Ali A. PX-12 synergistically enhances the therapeutic efficacy of vorinostat under hypoxic tumor microenvironment in oral squamous cell carcinoma in vitro. Drug Dev Res. 2023;84:556–60. 36808757 10.1002/ddr.22045

[505] Li GZ, Liang HF, Liao B, Zhang L, Ni YA, Zhou HH, Zhang EL, Zhang BX, Chen XP. PX-12 inhibits the growth of hepatocelluar carcinoma by inducing S-phase arrest, ROS-dependent apoptosis and enhances 5-FU cytotoxicity. Am J Transl Res. 2015;7:1528–40. 26550453 PMC4626415

[506] You BR, Shin HR, Park WH. PX-12 inhibits the growth of A549 lung cancer cells via G2/M phase arrest and ROS-dependent apoptosis. Int J Oncol. 2014;44:301–8. 24172913 10.3892/ijo.2013.2152

[507] Ramanathan RK, Kirkpatrick DL, Belani CP, Friedland D, Green SB, Chow HH, Cordova CA, Stratton SP, Sharlow ER, Baker A, Dragovich T. A phase I pharmacokinetic and pharmacodynamic study of PX-12, a novel inhibitor of thioredoxin-1, in patients with advanced solid tumors. Clin Cancer Res. 2007;13:2109–14. 17404093 10.1158/1078-0432.CCR-06-2250

[508] Ramanathan RK, Abbruzzese J, Dragovich T, Kirkpatrick L, Guillen JM, Baker AF, Pestano LA, Green S, Von Hoff DD. A randomized phase II study of PX-12, an inhibitor of thioredoxin in patients with advanced cancer of the pancreas following progression after a gemcitabine-containing combination. Cancer Chemother Pharmacol. 2011;67:503–9. 20461382 10.1007/s00280-010-1343-8

[509] Ramanathan RK, Stephenson JJ, Weiss GJ, Pestano LA, Lowe A, Hiscox A, Leos RA, Martin JC, Kirkpatrick L, Richards DA. A phase I trial of PX-12, a small-molecule inhibitor of thioredoxin-1, administered as a 72-hour infusion every 21 days in patients with advanced cancers refractory to standard therapy. Invest New Drugs. 2012;30:1591–6. 21863237 10.1007/s10637-011-9739-9

[510] Hashemy SI, Ungerstedt JS, Zahedi Avval F, Holmgren A. Motexafin gadolinium, a tumor-selective drug targeting thioredoxin reductase and ribonucleotide reductase. J Biol Chem. 2006;281:10691–7. 16481328 10.1074/jbc.M511373200

[511] Cao Y, Zhou X, Nie Q, Zhang J. Inhibition of the thioredoxin system for radiosensitization therapy of cancer. Eur J Med Chem. 2024;268:116218. 38387331 10.1016/j.ejmech.2024.116218

[512] Rosenthal DI, Nurenberg P, Becerra CR, Frenkel EP, Carbone DP, Lum BL, Miller R, Engel J, Young S, Miles D, Renschler MF. A phase I single-dose trial of gadolinium texaphyrin (Gd-Tex), a tumor selective radiation sensitizer detectable by magnetic resonance imaging. Clin Cancer Res. 1999;5:739–45. 10213207

[513] Viala J, Vanel D, Meingan P, Lartigau E, Carde P, Renschler M. Phases IB and II multidose trial of gadolinium texaphyrin, a radiation sensitizer detectable at MR imaging: preliminary results in brain metastases. Radiology. 1999;212:755–9. 10478243 10.1148/radiology.212.3.r99se10755

[514] Carde P, Timmerman R, Mehta MP, Koprowski CD, Ford J, Tishler RB, Miles D, Miller RA, Renschler MF. Multicenter phase Ib/II trial of the radiation enhancer motexafin gadolinium in patients with brain metastases. J Clin Oncol. 2001;19:2074–83. 11283141 10.1200/JCO.2001.19.7.2074

[515] Mehta MP, Shapiro WR, Glantz MJ, Patchell RA, Weitzner MA, Meyers CA, Schultz CJ, Roa WH, Leibenhaut M, Ford J, et al. Lead-in phase to randomized trial of motexafin gadolinium and whole-brain radiation for patients with brain metastases: centralized assessment of magnetic resonance imaging, neurocognitive, and neurologic end points. J Clin Oncol. 2002;20:3445–53. 12177105 10.1200/JCO.2002.07.500

[516] Mehta MP, Rodrigus P, Terhaard CH, Rao A, Suh J, Roa W, Souhami L, Bezjak A, Leibenhaut M, Komaki R, et al. Survival and neurologic outcomes in a randomized trial of motexafin gadolinium and whole-brain radiation therapy in brain metastases. J Clin Oncol. 2003;21:2529–36. 12829672 10.1200/JCO.2003.12.122

[517] Meyers CA, Smith JA, Bezjak A, Mehta MP, Liebmann J, Illidge T, Kunkler I, Caudrelier JM, Eisenberg PD, Meerwaldt J, et al. Neurocognitive function and progression in patients with brain metastases treated with whole-brain radiation and motexafin gadolinium: results of a randomized phase III trial. J Clin Oncol. 2004;22:157–65. 14701778 10.1200/JCO.2004.05.128

[518] Brachman DG, Pugh SL, Ashby LS, Thomas TA, Dunbar EM, Narayan S, Robins HI, Bovi JA, Rockhill JK, Won M, Curran WP. Phase 1/2 trials of temozolomide, motexafin gadolinium, and 60-Gy fractionated radiation for newly diagnosed supratentorial glioblastoma multiforme: final results of RTOG 0513. Int J Radiat Oncol Biol Phys. 2015;91:961–7. 25832688 10.1016/j.ijrobp.2014.12.050 PMC4706375

[519] Bradley KA, Zhou T, McNall-Knapp RY, Jakacki RI, Levy AS, Vezina G, Pollack IF. Motexafin-gadolinium and involved field radiation therapy for intrinsic pontine glioma of childhood: a children’s oncology group phase 2 study. Int J Radiat Oncol Biol Phys. 2013;85:e55-60. 23092726 10.1016/j.ijrobp.2012.09.004 PMC3529324

[520] Traynor AM, Thomas JP, Ramanathan RK, Mody TD, Alberti D, Wilding G, Bailey HH. Phase I trial of motexafin gadolinium and doxorubicin in the treatment of advanced malignancies. Invest New Drugs. 2011;29:316–22. 19997959 10.1007/s10637-009-9364-z PMC3038176

[521] Edelman MJ, Otterson G, Leach J, Malpass T, Salgia R, Jones D, Mody TD, Govindan R. Multicenter phase II trial of motexafin gadolinium and pemetrexed for second-line treatment in patients with non-small cell lung cancer. J Thorac Oncol. 2011;6:786–9. 21289521 10.1097/JTO.0b013e31820a443f

[522] Mertens RT, Gukathasan S, Arojojoye AS, Olelewe C, Awuah SG. Next generation gold drugs and probes: chemistry and biomedical applications. Chem Rev. 2023;123:6612–67. 37071737 10.1021/acs.chemrev.2c00649 PMC10317554

[523] Nobili S, Mini E, Landini I, Gabbiani C, Casini A, Messori L. Gold compounds as anticancer agents: chemistry, cellular pharmacology, and preclinical studies. Med Res Rev. 2010;30:550–80. 19634148 10.1002/med.20168

[524] Mármol I, Quero J, Rodríguez-Yoldi MJ, Cerrada E. Gold as a possible alternative to platinum-based chemotherapy for colon cancer treatment. Cancers (Basel). 2019;11:780. 31195711 10.3390/cancers11060780 PMC6628079

[525] Gamberi T, Chiappetta G, Fiaschi T, Modesti A, Sorbi F, Magherini F. Upgrade of an old drug: auranofin in innovative cancer therapies to overcome drug resistance and to increase drug effectiveness. Med Res Rev. 2022;42:1111–46. 34850406 10.1002/med.21872 PMC9299597

[526] Rousselle B, Massot A, Privat M, Dondaine L, Trommenschlager A, Bouyer F, Bayardon J, Ghiringhelli F, Bettaieb A, Goze C, et al. Conception and evaluation of fluorescent phosphine-gold complexes: from synthesis to in vivo investigations. ChemMedChem. 2022;17:e202100773. 35254001 10.1002/cmdc.202100773

[527] Xia Y, Chen J, Yu Y, Wu F, Shen X, Qiu C, Zhang T, Hong L, Zheng P, Shao R, et al. Compensatory combination of mTOR and TrxR inhibitors to cause oxidative stress and regression of tumors. Theranostics. 2021;11:4335–50. 33754064 10.7150/thno.52077 PMC7977446

[528] Gao M, Song Y, Liang J, Chen T, Luo J, Du P, Wang H, Leng H, Wang Z, Ma X, et al. Sensitizing ferroptotic and apoptotic cancer therapy via tailored micelles-mediated coenzyme and ATP depletion under hypoxia. J Control Release. 2025;381:113572. 40024339 10.1016/j.jconrel.2025.02.068

[529] Jamali F, Lan K, Daniel P, Petrecca K, Sabri S, Abdulkarim B. Synergistic dual targeting of thioredoxin and glutathione systems irrespective of p53 in glioblastoma stem cells. Antioxidants (Basel). 2024;13:1201. 39456455 10.3390/antiox13101201 PMC11504866

[530] Yang M, Liu J, Li J, Wen S, Hu Y, Lu W, Liu J, Huang P, Liu P. The rheumatoid arthritis drug auranofin exerts potent anti-lymphoma effect by stimulating TXNRD-mediated ROS generation and inhibition of energy metabolism. Redox Biol. 2024;75:103245. 38909408 10.1016/j.redox.2024.103245 PMC11254835

[531] Wang L, Yang Z, Fu J, Yin H, Xiong K, Tan Q, Jin H, Li J, Wang T, Tang W, et al. Ethaselen: a potent mammalian thioredoxin reductase 1 inhibitor and novel organoselenium anticancer agent. Free Radic Biol Med. 2012;52:898–908. 22210352 10.1016/j.freeradbiomed.2011.11.034

[532] Woo HA, Jeong W, Chang TS, Park KJ, Park SJ, Yang JS, Rhee SG. Reduction of cysteine sulfinic acid by sulfiredoxin is specific to 2-cys peroxiredoxins. J Biol Chem. 2005;280:3125–8. 15590625 10.1074/jbc.C400496200

[533] Ran H, Liu H, Wu P. Echinatin mitigates H2O2-induced oxidative damage and apoptosis in lens epithelial cells via the Nrf2/HO-1 pathway. Adv Clin Exp Med. 2021;30:1195–203. 34510844 10.17219/acem/139130

[534] Zheng X, Zhang Y, Zhang L, Xu W, Ma W, Sun R, Zeng H. Synergistic inhibition of sunitinib and ethaselen against human colorectal cancer cells proliferation. Biomed Pharmacother. 2016;83:212–20. 27372405 10.1016/j.biopha.2016.06.040

[535] Zheng X, Xu W, Sun R, Yin H, Dong C, Zeng H. Synergism between thioredoxin reductase inhibitor ethaselen and sodium selenite in inhibiting proliferation and inducing death of human non-small cell lung cancer cells. Chem Biol Interact. 2017;275:74–85. 28757135 10.1016/j.cbi.2017.07.020

[536] Zheng X, Ma W, Sun R, Yin H, Lin F, Liu Y, Xu W, Zeng H. Butaselen prevents hepatocarcinogenesis and progression through inhibiting thioredoxin reductase activity. Redox Biol. 2018;14:237–49. 28965082 10.1016/j.redox.2017.09.014 PMC5633849

[537] Zou Q, Chen YF, Zheng XQ, Ye SF, Xu BY, Liu YX, Zeng HH. Novel thioredoxin reductase inhibitor butaselen inhibits tumorigenesis by down-regulating programmed death-ligand 1 expression. J Zhejiang Univ Sci B. 2018;19:689–98. 30178635 10.1631/jzus.B1700219 PMC6137417

[538] Su X, Yin H, Bai M, Liu J, Liu R, Zeng H, Wen J. A novel TrxR1 inhibitor regulates NK and CD8+ T cell infiltration and cytotoxicity, enhancing the efficacy of anti-PD-1 immunotherapy against hepatocarcinoma. J Immunol. 2023;210:681–95. 36602827 10.4049/jimmunol.2200389

[539] Li Y, Wang N, Li H, Zhang X, Meng L, Yu Y, Wang S, Deng L. Biomineralization of copper-celastrol nanohybrids for synergistic antitumor therapy. Small. 2025;21:e2412802. 40095444 10.1002/smll.202412802

[540] Liu Y, Song J, Guo Y, Li S, Yuan M, Tang J, Wang Y, Li M, Guo Y, Guo L. Synergistic therapy with celastrol-curcumin multifunctional nanomedicine: anti-hepatocellular carcinoma and reduced hepatotoxicity. Int J Pharm. 2025;671:125289. 39880142 10.1016/j.ijpharm.2025.125289

[541] Ma C, Wang F, Wang Y, Wu F, Zhang X, Ding C, Zhao J, Ma Y, Li W, Liu W. Discovery of the novel celastrol-based PROTACs for the treatment of non-small cell lung cancer. Mol Divers. 2025. 10.1007/s11030-025-11140-7 39964654

[542] Zhang Z, Wang J, Li X, Zhao L, Zhao J, Su M, Wu X, Zeng H. Synergistic effect of pH-sensitive PEGylated RG3-chitosan prodrug nanoparticles encapsulated celastrol on pancreatic cancer. Drug Deliv. 2025;32:2464189. 39957204 10.1080/10717544.2025.2464189 PMC11834771

[543] Huang J, Shi J, Ma N, Li Y, Jin W, Zhang H, Zhang X, Luo N, Ding Y, Xie Q, et al. Celastrol-loaded ginsenoside Rg3 liposomes enhance anti-programmed death ligand 1 immunotherapy by inducing immunogenic cell death in triple-negative breast cancer. Phytomedicine. 2025;139:156514. 39986227 10.1016/j.phymed.2025.156514

[544] Liu Y, Zhang J, Lai C, Wang W, Huang Y, Bao X, Yan H, Sun X, Liu Q, Chen D, et al. Injectable celastrol-loading emulsion hydrogel for immunotherapy of low-immunogenic cancer. J Nanobiotechnology. 2025;23:183. 40050985 10.1186/s12951-025-03154-y PMC11887069

[545] Huang P, Feng L, Oldham EA, Keating MJ, Plunkett W. Superoxide dismutase as a target for the selective killing of cancer c ells. Nature. 2000;407:390–5. 11014196 10.1038/35030140

[546] Kachadourian R, Liochev SI, Cabelli DE, Patel MN, Fridovich I, Day BJ. 2-methoxyestradiol does not inhibit superoxide dismutase. Arch Biochem Biophys. 2001;392:349–53. 11488612 10.1006/abbi.2001.2455

[547] Kesarwani P, Al-Khami AA, Scurti G, Thyagarajan K, Kaur N, Husain S, Fang Q, Naga OS, Simms P, Beeson G, et al. Promoting thiol expression increases the durability of antitumor T-cell functions. Cancer Res. 2014;74:6036–47. 25164014 10.1158/0008-5472.CAN-14-1084 PMC4216764

[548] Lee JB, Khan DH, Hurren R, Xu M, Na Y, Kang H, Mirali S, Wang X, Gronda M, Jitkova Y, et al. Venetoclax enhances T cell-mediated antileukemic activity by increasing ROS production. Blood. 2021;138:234–45. 34292323 10.1182/blood.2020009081 PMC8310428

[549] Zheng Y, Wang Y, Lu Z, Wan J, Jiang L, Song D, Wei C, Gao C, Shi G, Zhou J, et al. PGAM1 inhibition promotes HCC ferroptosis and synergizes with anti-PD-1 immunotherapy. Adv Sci (Weinh). 2023;10:e2301928. 37705495 10.1002/advs.202301928 PMC10582428

[550] Yang Y, Neo SY, Chen Z, Cui W, Chen Y, Guo M, Wang Y, Xu H, Kurzay A, Alici E, et al. Thioredoxin activity confers resistance against oxidative stress in tumor-infiltrating NK cells. J Clin Invest. 2020;130:5508–22. 32673292 10.1172/JCI137585 PMC7524507

[551] Kalafati L, Kourtzelis I, Schulte-Schrepping J, Li X, Hatzioannou A, Grinenko T, Hagag E, Sinha A, Has C, Dietz S, et al. Innate immune training of granulopoiesis promotes anti-tumor activity. Cell. 2020;183:771-785.e712. 33125892 10.1016/j.cell.2020.09.058 PMC7599076

[552] Sampson N, Brunner E, Weber A, Puhr M, Schäfer G, Szyndralewiez C, Klocker H. Inhibition of Nox4-dependent ROS signaling attenuates prostate fibroblast activation and abrogates stromal-mediated protumorigenic interactions. Int J Cancer. 2018;143:383–95. 29441570 10.1002/ijc.31316 PMC6067067

[553] Ford K, Hanley CJ, Mellone M, Szyndralewiez C, Heitz F, Wiesel P, Wood O, Machado M, Lopez MA, Ganesan AP, et al. NOX4 inhibition potentiates immunotherapy by overcoming cancer-associated fibroblast-mediated CD8 T-cell exclusion from tumors. Cancer Res. 2020;80:1846–60. 32122909 10.1158/0008-5472.CAN-19-3158 PMC7611230

[554] Ding D, Zhong H, Liang R, Lan T, Zhu X, Huang S, Wang Y, Shao J, Shuai X, Wei B. Multifunctional nanodrug mediates synergistic photodynamic therapy and MDSCs-targeting immunotherapy of colon cancer. Adv Sci (Weinh). 2021;8:e2100712. 34021727 10.1002/advs.202100712 PMC8292876

[555] Jiang M, Li X, Zhang J, Lu Y, Shi Y, Zhu C, Liu Y, Qin B, Luo Z, Du Y, et al. Dual inhibition of endoplasmic reticulum stress and oxidation stress manipulates the polarization of macrophages under hypoxia to sensitize immunotherapy. ACS Nano. 2021;15:14522–34. 34414762 10.1021/acsnano.1c04068

[556] Cao Y, Qiao B, Chen Q, Xie Z, Dou X, Xu L, Ran H, Zhang L, Wang Z. Tumor microenvironment remodeling via targeted depletion of M2-like tumor-associated macrophages for cancer immunotherapy. Acta Biomater. 2023;160:239–51. 36774974 10.1016/j.actbio.2023.02.006

[557] Kroemer G, Galluzzi L, Kepp O, Zitvogel L. Immunogenic cell death in cancer therapy. Annu Rev Immunol. 2013;31:51–72. 23157435 10.1146/annurev-immunol-032712-100008

[558] Krysko DV, Garg AD, Kaczmarek A, Krysko O, Agostinis P, Vandenabeele P. Immunogenic cell death and DAMPs in cancer therapy. Nat Rev Cancer. 2012;12:860–75. 23151605 10.1038/nrc3380

[559] Radogna F, Diederich M. Stress-induced cellular responses in immunogenic cell death: implications for cancer immunotherapy. Biochem Pharmacol. 2018;153:12–23. 29438676 10.1016/j.bcp.2018.02.006

[560] Shi C, Liu T, Guo Z, Zhuang R, Zhang X, Chen X. Reprogramming tumor-associated macrophages by nanoparticle-based reactive oxygen species photogeneration. Nano Lett. 2018;18:7330–42. 30339753 10.1021/acs.nanolett.8b03568

[561] Wen M, Ouyang J, Wei C, Li H, Chen W, Liu YN. Artificial enzyme catalyzed cascade reactions: antitumor immunotherapy reinforced by NIR-II light. Angew Chem Int Ed Engl. 2019;58:17425–32. 31552695 10.1002/anie.201909729

[562] Teng Y, Yang Z, Peng Y, Yang Y, Chen S, Li J, Gao D, Sun W, Wu Z, Zhou Y, et al. Endoplasmic reticulum stress nano-orchestrators for precisely regulated immunogenic cell death as potent cancer vaccines. Adv Healthc Mater. 2025;14:e2401851. 39449212 10.1002/adhm.202401851

[563] Vander Heiden MG, Cantley LC, Thompson CB. Understanding the Warburg effect: the metabolic requirements of cell proliferation. Science. 2009;324:1029–33. 19460998 10.1126/science.1160809 PMC2849637

[564] He R, Zang J, Zhao Y, Liu Y, Ruan S, Zheng X, Chong G, Xu D, Yang Y, Yang Y, et al. Nanofactory for metabolic and chemodynamic therapy: pro-tumor lactate trapping and anti-tumor ROS transition. J Nanobiotechnol. 2021;19:426. 10.1186/s12951-021-01169-9 PMC8684183 34922541

[565] Li Z, Chu Z, Yang J, Qian H, Xu J, Chen B, Tian T, Chen H, Xu Y, Wang F. Immunogenic cell death augmented by manganese zinc sulfide nanoparticles for metastatic melanoma immunotherapy. ACS Nano. 2022;16:15471–83. 35981098 10.1021/acsnano.2c08013

[566] Menger L, Vacchelli E, Adjemian S, Martins I, Ma Y, Shen S, Yamazaki T, Sukkurwala AQ, Michaud M, Mignot G, et al. Cardiac glycosides exert anticancer effects by inducing immunogenic cell death. Sci Transl Med. 2012;4:143ra199. 10.1126/scitranslmed.3003807 22814852

[567] Xu Z, Xu J, Sun S, Lin W, Li Y, Lu Q, Li F, Yang Z, Lu Y, Liu W. Mecheliolide elicits ROS-mediated ERS driven immunogenic cell death in hepatocellular carcinoma. Redox Biol. 2022;54:102351. 35671636 10.1016/j.redox.2022.102351 PMC9168183

[568] Wan J, Zhang X, Li Z, Mo F, Tang D, Xiao H, Wang J, Rong G, Liu T. Oxidative stress amplifiers as immunogenic cell death nanoinducers disrupting mitochondrial redox homeostasis for cancer immunotherapy. Adv Healthc Mater. 2023;12:e2202710. 36527737 10.1002/adhm.202202710

[569] Mossakowska BJ, Shahmoradi Ghahe S, Cysewski D, Fabisiewicz A, Tudek B, Siedlecki JA. Mechanisms of resistance to photodynamic therapy (PDT) in vulvar cancer. Int J Mol Sci. 2022;23:4117. 35456936 10.3390/ijms23084117 PMC9028356

[570] Wei MF, Chen MW, Chen KC, Lou PJ, Lin SYF, Hung SC, Hsiao M, Yao CJ, Shieh MJ. Autophagy promotes resistance to photodynamic therapy-induced apoptosis selectively in colorectal cancer stem-like cells. Autophagy. 2014;10:1179–92. 24905352 10.4161/auto.28679 PMC4203546

[571] Hui KF, Yeung PL, Chiang AK. Induction of MAPK- and ROS-dependent autophagy and apoptosis in gastric carcinoma by combination of romidepsin and bortezomib. Oncotarget. 2016;7:4454–67. 26683357 10.18632/oncotarget.6601 PMC4826218

[572] Li X, Zhang W, Xing Z, Hu S, Zhang G, Wang T, Wang T, Fan Q, Chen G, Cheng J, et al. Targeting SIRT3 sensitizes glioblastoma to ferroptosis by promoting mitophagy and inhibiting SLC7A11. Cell Death Dis. 2024;15:168. 38395990 10.1038/s41419-024-06558-0 PMC10891132

[573] Fu LH, Wan Y, Qi C, He J, Li C, Yang C, Xu H, Lin J, Huang P. Nanocatalytic theranostics with glutathione depletion and enhanced reactive oxygen species generation for efficient cancer therapy. Adv Mater. 2021;33:e2006892. 33394515 10.1002/adma.202006892

[574] Xiao H, Li X, Li B, Yang S, Qin J, Han S, Ren J, Shuai X. Nanodrug inducing autophagy inhibition and mitochondria dysfunction for potentiating tumor photo-immunotherapy. Small. 2023;19:e2300280. 37060227 10.1002/smll.202300280

[575] Chen M, Yang J, Zhou L, Hu X, Wang C, Chai K, Li R, Feng L, Sun Y, Dong C, Shi S. Dual-responsive and ROS-augmented nanoplatform for chemo/photodynamic/chemodynamic combination therapy of triple negative breast cancer. ACS Appl Mater Interfaces. 2022;14:57–68. 34935343 10.1021/acsami.1c14135

[576] Xiong Y, Xiao C, Li Z, Yang X. Engineering nanomedicine for glutathione depletion-augmented cancer therapy. Chem Soc Rev. 2021;50:6013–41. 34027953 10.1039/d0cs00718h

[577] Xiao Y, Zhang T, Ma X, Yang QC, Yang LL, Yang SC, Liang M, Xu Z, Sun ZJ. Microenvironment-responsive prodrug-induced pyroptosis boosts cancer immunotherapy. Adv Sci (Weinh). 2021;8:e2101840. 34705343 10.1002/advs.202101840 PMC8693073

[578] Wang X, Ding H, Li Z, Peng Y, Tan H, Wang C, Huang G, Li W, Ma G, Wei W. Exploration and functionalization of M1-macrophage extracellular vesicles for effective accumulation in glioblastoma and strong synergistic therapeutic effects. Signal Transduct Target Ther. 2022;7:74. 35292619 10.1038/s41392-022-00894-3 PMC8924195

[579] Ji C, Si J, Xu Y, Zhang W, Yang Y, He X, Xu H, Mou X, Ren H, Guo H. Mitochondria-targeted and ultrasound-responsive nanoparticles for oxygen and nitric oxide codelivery to reverse immunosuppression and enhance sonodynamic therapy for immune activation. Theranostics. 2021;11:8587–604. 34373760 10.7150/thno.62572 PMC8344010

[580] Wang D, Wang T, Yu H, Feng B, Zhou L, Zhou F, Hou B, Zhang H, Luo M, Li Y. Engineering nanoparticles to locally activate T cells in the tumor microenvironment. Sci Immunol. 2019;4:eaau6584. 31300478 10.1126/sciimmunol.aau6584

[581] Wang M, Chang M, Li C, Chen Q, Hou Z, Xing B, Lin J. Tumor-microenvironment-activated reactive oxygen species amplifier for enzymatic cascade cancer starvation/chemodynamic /immunotherapy. Adv Mater. 2022;34:e2106010. 34699627 10.1002/adma.202106010

[582] Li J, Wang S, Lin X, Cao Y, Cai Z, Wang J, Zhang Z, Liu X, Wu M, Yao C. Red blood cell-mimic nanocatalyst triggering radical storm to augment cancer immunotherapy. Nanomicro Lett. 2022;14:57. 35122163 10.1007/s40820-022-00801-z PMC8817004

[583] Gong M, Huang Y, Feng H, Lin J, Huang A, Hu J, Tang Q, Zhu X, Han S, Lu J, Wang J. A nanodrug combining CD47 and sonodynamic therapy efficiently inhibits osteosarcoma deterioration. J Control Release. 2023;355:68–84. 36682726 10.1016/j.jconrel.2023.01.038

[584] He M, Wang M, Xu T, Zhang M, Dai H, Wang C, Ding D, Zhong Z. Reactive oxygen species-powered cancer immunotherapy: current status and challenges. J Control Release. 2023;356:623–48. 36868519 10.1016/j.jconrel.2023.02.040

[585] Zhao C, Zheng T, Wang R, Lin X, Hu Z, Zhao Z, Dai Z, Sun D. Synergistically augmenting cancer immunotherapy by physical manipulation of pyroptosis induction. Phenomics. 2024;4:298–312. 39398428 10.1007/s43657-023-00140-y PMC11466912

[586] Pantic I, Paunovic J, Pejic S, Drakulic D, Todorovic A, Stankovic S, Vucevic D, Cumic J, Radosavljevic T. Artificial intelligence approaches to the biochemistry of oxidative stress: current state of the art. Chem Biol Interact. 2022;358:109888. 35296431 10.1016/j.cbi.2022.109888

[587] Xu M, Zhou H, Hu P, Pan Y, Wang S, Liu L, Liu X. Identification and validation of immune and oxidative stress-related diagnostic markers for diabetic nephropathy by WGCNA and machine learning. Front Immunol. 2023;14:1084531. 36911691 10.3389/fimmu.2023.1084531 PMC9992203

[588] Ho Thanh Lam L, Le NH, Van Tuan L, Tran Ban H, Nguyen Khanh Hung T, Nguyen NTK, Huu Dang L, Le NQK. Machine learning model for identifying antioxidant proteins using features calculated from primary sequences. Biology (Basel). 2020;9:325. 33036150 10.3390/biology9100325 PMC7599600

[589] Idowu SO, Fatokun AA. Artificial Intelligence (AI) to the rescue: deploying machine learning to bridge the biorelevance gap in antioxidant assays. SLAS Technol. 2021;26:16–25. 33054529 10.1177/2472630320962716 PMC7838339

[590] Mirzaei M, Furxhi I, Murphy F, Mullins M. Employing supervised algorithms for the prediction of nanomaterial’s antioxidant efficiency. Int J Mol Sci. 2023;24:2792. 36769135 10.3390/ijms24032792 PMC9918003

[591] Dong L, Li W, Sun L, Yu L, Chen Y, Hong G. Energy-converting biomaterials for cancer therapy: category, efficiency, and biosafety. Wiley Interdiscip Rev Nanomed Nanobiotechnol. 2021;13:e1663. 32808464 10.1002/wnan.1663

[592] Chen J, Feng C, Lan Y, Chen X, Peng Z, Huang Z, Wang R, Zhang W, Ye Y, Mao Z, et al. Bidirectional regulation of reactive oxygen species for radiosensitization in nasopharyngeal carcinoma. J Nanobiotechnol. 2025;23:96. 10.1186/s12951-025-03177-5 PMC11806541 39923065

[593] Zhu S, Huo L, Zeng J, Chen R, Sun Y, Tan M, Fan M, Liu M, Zhao J, Huang G, et al. Differentiated management of ROS level in tumor and kidney to alleviate Cis-platinum induced acute kidney injury with improved efficacy. J Nanobiotechnol. 2024;22:436. 10.1186/s12951-024-02710-2 PMC11267679 39044240

[594] Nie R, Zhang J, Jia Q, Li Y, Tao W, Qin G, Liu X, Tao Y, Zhang Y, Li P. Structurally oriented carbon dots as ROS nanomodulators for dynamic chronic inflammation and infection elimination. ACS Nano. 2024;18:22055–70. 39116283 10.1021/acsnano.4c05266

[595] Martin JR, Gupta MK, Page JM, Yu F, Davidson JM, Guelcher SA, Duvall CL. A porous tissue engineering scaffold selectively degraded by cell-generated reactive oxygen species. Biomaterials. 2014;35:3766–76. 24491510 10.1016/j.biomaterials.2014.01.026 PMC3975079

[596] Chao Y, Chen Q, Liu Z. Smart injectable hydrogels for cancer immunotherapy. Adv Func Mater. 2020;30:1902785.

[597] Robby AI, Yang JH, Jin EJ, Park SY. Tumor microenvironment-selective sol-gel mineralization of ROS-responsive stretchable and conductive hydrogel. Adv Funct Mater. 2024;34:2402367.

[598] Liu J, Han X, Zhang T, Tian K, Li Z, Luo F. Reactive oxygen species (ROS) scavenging biomaterials for anti-inflammatory diseases: from mechanism to therapy. J Hematol Oncol. 2023;16:116. 38037103 10.1186/s13045-023-01512-7 PMC10687997

[599] Ivanova DG, Yaneva ZL. Antioxidant properties and redox-modulating activity of chitosan and its derivatives: biomaterials with application in cancer therapy. Biores Open Access. 2020;9:64–72. 32219012 10.1089/biores.2019.0028 PMC7097683

[600] Greenwood HE, Witney TH. Latest advances in imaging oxidative stress in cancer. J Nucl Med. 2021;62:1506–10. 34353871 10.2967/jnumed.120.256974 PMC7611938

[601] Geng Y, Wang Z, Zhou J, Zhu M, Liu J, James TD. Recent progress in the development of fluorescent probes for imaging pathological oxidative stress. Chem Soc Rev. 2023;52:3873–926. 37190785 10.1039/d2cs00172a

[602] Yang G, Wang W, Song J, Zhang J. Bioresponsive self-illuminating nanoparticles for luminescence imaging of inflammation and oxidative stress. Nanomedicine (Lond). 2021;16:1737–40. 34196221 10.2217/nnm-2021-0129

[603] Xu X, An H, Zhang D, Tao H, Dou Y, Li X, Huang J, Zhang J. A self-illuminating nanoparticle for inflammation imaging and cancer therapy. Sci Adv. 2019;5:eaat2953. 30662940 10.1126/sciadv.aat2953 PMC6326751

[604] Yan X, Lin W, Liu H, Pu W, Li J, Wu P, Ding J, Luo G, Zhang J. Wavelength-tunable, long lifetime, and biocompatible luminescent nanoparticles based on a vitamin E-derived material for inflammation and tumor imaging. Small. 2021;17:e2100045. 34031977 10.1002/smll.202100045

[605] Cui Y, Wang X, Jiang Z, Zhang C, Liang Z, Chen Y, Liu Z, Guo Z. A photoacoustic probe with blood-brain barrier crossing ability for imaging oxidative stress dynamics in the mouse brain. Angew Chem Int Ed Engl. 2023;62:e202214505. 36597890 10.1002/anie.202214505

[606] Lazarova D, Semkova S, Zlateva G, Tatsuya H, Aoki I, Bakalova R. Quantum sensors to track total redox-status and oxidative stress in cells and tissues using electron-paramagnetic resonance, magnetic resonance imaging, and optical imaging. Anal Chem. 2021;93:2828–37. 33508934 10.1021/acs.analchem.0c04116

[607] Balke J, Volz P, Neumann F, Brodwolf R, Wolf A, Pischon H, Radbruch M, Mundhenk L, Gruber AD, Ma N, Alexiev U. Visualizing oxidative cellular stress induced by nanoparticles in the subcytotoxic range using fluorescence lifetime imaging. Small. 2018;14:e1800310. 29726099 10.1002/smll.201800310

[608] Webster JM, Morton CA, Johnson BF, Yang H, Rishel MJ, Lee BD, Miao Q, Pabba C, Yapp DT, Schaffer P. Functional imaging of oxidative stress with a novel PET imaging agent, 18F-5-fluoro-L-aminosuberic acid. J Nucl Med. 2014;55:657–64. 24578242 10.2967/jnumed.113.126664 PMC4009729

[609] Li J, Pan L, Pan W, Li N, Tang B. Recent progress of oxidative stress associated biomarker detection. Chem Commun (Camb). 2023;59:7361–74. 37194341 10.1039/d3cc00878a

[610] Liu J, Liu M, Zhang H, Guo W. High-contrast fluorescence diagnosis of cancer cells/tissues based on β-lapachone-triggered ROS amplification specific in cancer cells. Angew Chem Int Ed Engl. 2021;60:12992–8. 33772992 10.1002/anie.202102377

[611] Liu F, Wang Z, Wang W, Luo JG, Kong L. Red-emitting fluorescent probe for detection of γ-glutamyltranspeptidase and its application of real-time imaging under oxidative stress in cells and in vivo. Anal Chem. 2018;90:7467–73. 29785851 10.1021/acs.analchem.8b00994

[612] Cheung EC, DeNicola GM, Nixon C, Blyth K, Labuschagne CF, Tuveson DA, Vousden KH. Dynamic ROS control by TIGAR regulates the initiation and progression of pancreatic cancer. Cancer Cell. 2020;37:168-182.e164. 31983610 10.1016/j.ccell.2019.12.012 PMC7008247

[613] Chen Z, Tian R, She Z, Cai J, Li H. Role of oxidative stress in the pathogenesis of nonalcoholic fatty liver disease. Free Radic Biol Med. 2020;152:116–41. 32156524 10.1016/j.freeradbiomed.2020.02.025

[614] Wen J, Li A, Wang Z, Guo X, Zhang G, Litzow MR, Liu Q. Hepatotoxicity induced by arsenic trioxide: clinical features, mechanisms, preventive and potential therapeutic strategies. Front Pharmacol. 2025;16:1536388. 40051569 10.3389/fphar.2025.1536388 PMC11882591

[615] Songbo M, Lang H, Xinyong C, Bin X, Ping Z, Liang S. Oxidative stress injury in doxorubicin-induced cardiotoxicity. Toxicol Lett. 2019;307:41–8. 30817977 10.1016/j.toxlet.2019.02.013

[616] Chiu GS, Maj MA, Rizvi S, Dantzer R, Vichaya EG, Laumet G, Kavelaars A, Heijnen CJ. Pifithrin-μ prevents cisplatin-induced chemobrain by preserving neuronal mitochondrial function. Cancer Res. 2017;77:742–52. 27879267 10.1158/0008-5472.CAN-16-1817 PMC5290207

[617] Chen Z, Han F, Du Y, Shi H, Zhou W. Hypoxic microenvironment in cancer: molecular mechanisms and therapeutic interventions. Signal Transduct Target Ther. 2023;8:70. 36797231 10.1038/s41392-023-01332-8 PMC9935926

[618] Zhao Z, Ukidve A, Kim J, Mitragotri S. Targeting strategies for tissue-specific drug delivery. Cell. 2020;181:151–67. 32243788 10.1016/j.cell.2020.02.001

[619] Modak M, Bobbala S, Lescott C, Liu YG, Nandwana V, Dravid VP, Scott EA. Magnetic nanostructure-loaded bicontinuous nanospheres support multicargo intracellular delivery and oxidation-responsive morphological transitions. ACS Appl Mater Interfaces. 2020;12:55584–95. 33259182 10.1021/acsami.0c15920

[620] Inukai N, Yamaguchi Y, Kuraoka I, Yamada T, Kamijo S, Kato J, Tanaka K, Handa H. A novel hydrogen peroxide-induced phosphorylation and ubiquitination pathway leading to RNA polymerase II proteolysis. J Biol Chem. 2004;279:8190–5. 14662762 10.1074/jbc.M311412200

[621] Petsouki E, Cabrera SNS, Heiss EH. AMPK and NRF2: interactive players in the same team for cellular homeostasis? Free Radic Biol Med. 2022;190:75–93. 35918013 10.1016/j.freeradbiomed.2022.07.014

[622] Elhanani O, Ben-Uri R, Keren L. Spatial profiling technologies illuminate the tumor microenvironment. Cancer Cell. 2023;41:404–20. 36800999 10.1016/j.ccell.2023.01.010

[623] Wang Y, Jia J, Wang F, Fang Y, Yang Y, Zhou Q, Yuan W, Gu X, Hu J, Yang S. Pre-metastatic niche: formation, characteristics and therapeutic implication. Signal Transduct Target Ther. 2024;9:236. 39317708 10.1038/s41392-024-01937-7 PMC11422510

[624] Nakamura J, La DK, Swenberg JA. 5’-nicked apurinic/apyrimidinic sites are resistant to beta-elimination by beta-polymerase and are persistent in human cultured cells after oxidative stress. J Biol Chem. 2000;275:5323–8. 10681505 10.1074/jbc.275.8.5323

[625] Chen X, Zhao Y, Luo W, Chen S, Lin F, Zhang X, Fan S, Shen X, Wang Y, Liang G. Celastrol induces ROS-mediated apoptosis via directly targeting peroxi redoxin-2 in gastric cancer cells. Theranostics. 2020;10:10290–308. 32929349 10.7150/thno.46728 PMC7481428

[626] Lei K, Gu X, Alvarado AG, Du Y, Luo S, Ahn EH, Kang SS, Ji B, Liu X, Mao H, et al. Discovery of a dual inhibitor of NQO1 and GSTP1 for treating glioblastoma. J Hematol Oncol. 2020;13:141. 33087132 10.1186/s13045-020-00979-y PMC7579906

[627] Assi M. The differential role of reactive oxygen species in early and late stages of cancer. Am J Physiol Regul Integr Comp Physiol. 2017;313:R646-r653. 28835450 10.1152/ajpregu.00247.2017

[628] Liu M, Fan Y, Li D, Han B, Meng Y, Chen F, Liu T, Song Z, Han Y, Huang L, et al. Ferroptosis inducer erastin sensitizes NSCLC cells to celastrol through activation of the ROS-mitochondrial fission-mitophagy axis. Mol Oncol. 2021;15:2084–105. 33675143 10.1002/1878-0261.12936 PMC8334255

[629] Suzuki T, Takahashi J, Yamamoto M. Molecular basis of the KEAP1-NRF2 signaling pathway. Mol Cells. 2023;46:133–41. 36994473 10.14348/molcells.2023.0028 PMC10070164

[630] Selvaggio G, Coelho P, Salvador A. Mapping the phenotypic repertoire of the cytoplasmic 2-Cys peroxiredoxin - thioredoxin system. 1. Understanding commonalities and differences among cell types. Redox Biol. 2018;15:297–315. 29304480 10.1016/j.redox.2017.12.008 PMC5975082

[631] Petermann E, Lan L, Zou L. Sources, resolution and physiological relevance of R-loops and RNA-DNA hybrids. Nat Rev Mol Cell Biol. 2022;23:521–40. 35459910 10.1038/s41580-022-00474-x

[632] Suzuki T, Muramatsu A, Saito R, Iso T, Shibata T, Kuwata K, Kawaguchi SI, Iwawaki T, Adachi S, Suda H, et al. Molecular mechanism of cellular oxidative stress sensing by Keap1. Cell Rep. 2019;28:746-758.e744. 31315052 10.1016/j.celrep.2019.06.047

